# Effectidor II: a pan-genomic AI-based algorithm for the prediction of type III secretion system effectors

**DOI:** 10.1093/bioinformatics/btaf272

**Published:** 2025-04-29

**Authors:** Naama Wagner, Ella Baumer, Iris Lyubman, Yair Shimony, Noam Bracha, Leonor Martins, Neha Potnis, Jeff H Chang, Doron Teper, Ralf Koebnik, Tal Pupko

**Affiliations:** The Shmunis School of Biomedicine and Cancer Research, George S. Wise Faculty of Life Sciences, Tel Aviv University, Chaim Levanon St 30, Tel Aviv, 69978, Israel; The Shmunis School of Biomedicine and Cancer Research, George S. Wise Faculty of Life Sciences, Tel Aviv University, Chaim Levanon St 30, Tel Aviv, 69978, Israel; The Shmunis School of Biomedicine and Cancer Research, George S. Wise Faculty of Life Sciences, Tel Aviv University, Chaim Levanon St 30, Tel Aviv, 69978, Israel; The Shmunis School of Biomedicine and Cancer Research, George S. Wise Faculty of Life Sciences, Tel Aviv University, Chaim Levanon St 30, Tel Aviv, 69978, Israel; The Shmunis School of Biomedicine and Cancer Research, George S. Wise Faculty of Life Sciences, Tel Aviv University, Chaim Levanon St 30, Tel Aviv, 69978, Israel; CIBIO—Centro de Investigação em Biodiversidade e Recursos Genéticos, InBIO—Laboratório Associado, Universidade do Porto, Vairão, 4485-661, Portugal; FCUP—Faculdade de Ciências, Departamento de Biologia, Universidade do Porto, Porto, 4169-007, Portugal; Department of Entomology and Plant Pathology, Auburn University, Auburn, AL, 36849, United States of America; Department of Botany and Plant Pathology, Oregon State University, Corvallis, OR, 97331, United States of America; Department of Plant Pathology and Weed Research, Institute of Plant Protection Agricultural Research Organization (ARO), Volcani Center, Rishon LeZion, 7505101, Israel; Plant Health Institute of Montpellier, University of Montpellier, CIRAD, INRAe, Institut Agro, IRD, Montpellier, 34394, France; The Shmunis School of Biomedicine and Cancer Research, George S. Wise Faculty of Life Sciences, Tel Aviv University, Chaim Levanon St 30, Tel Aviv, 69978, Israel

## Abstract

**Motivation:**

Type III secretion systems are used by many Gram-negative bacteria to inject type 3 effectors (T3Es) directly into eukaryotic cells, promoting disease or provoking immune response. Because of these opposing evolutionary forces, T3E repertoires often vary within taxonomic groups. Identifying the full effector gene repertoire in genomes of related individuals is crucial for determining core and specialized effectors, understanding the disease dynamics, and developing appropriate management strategies against pathogens. It can also help uncover novel T3Es that have recently emerged in a population. Our previously published Effectidor web server successfully addressed the challenge of identifying T3Es in a single bacterial genome. Here, we enriched the web server with various novel capabilities, including the identification of T3Es from multiple genome sequences simultaneously.

**Results:**

We present Effectidor II, a web server that relies on machine learning to predict T3E-encoding genes within bacterial pan-genomes. We demonstrate the benefit of learning based on features extracted from the entire sequences comprising the pan-genome and report a novel T3E discovered by it in *Xanthomonas euroxanthea*.

**Availability and implementation:**

Effectidor II is available at: https://effectidor.tau.ac.il and the source code is available at: https://github.com/naamawagner/Effectidor. A stand-alone version of Effectidor II is available at: https://github.com/naamawagner/Effectidor/tree/StandAlone. The source code for the standalone version and the data used in this work are also provided in https://doi.org/10.5281/zenodo.15081636.

## 1 Introduction

Gram-negative pathogenic bacteria use type III secretion systems (T3SSs) to translocate effector proteins (T3Es) directly into eukaryotic hosts, leading to disease or immune response in susceptible or resistant species, respectively (Lindgren *et al.* 1986). These effectors exhibit diversity in sequences, structures, and functions within a given genome and among different strains within taxonomic groups (Jiménez-Guerrero *et al.* 2020). A large number of methodologies were previously used to identify T3Es (e.g. Lovelace *et al.* 2023, Zhao *et al.* 2023). We have previously developed Effectidor, a web server that relies on machine learning (ML) for the prediction of T3E genes within an input bacterial genome sequence (Wagner *et al.* 2022b). Unlike other tools, Effectidor retrains the ML models to fit every input genome anew. This way, predictions are based on a genome-specific model. Since its publication, it has successfully been used to identify novel T3Es of diverse bacterial pathogens (Ranković *et al.* 2023, Wagner *et al.* 2023). However, it did not support the analysis of multiple genomes simultaneously, which is of high interest for comparative genomics, as was done in the works of Ranković *et al.* (2023) and Monnens *et al.* (2024). Similarly, it is unsuitable for genomes with too few known T3Es, as they are insufficient for training ML models.

Here, we present Effectidor II, which addresses these limitations, among others. First, it enables pan-genomic analyses by clustering open reading frames (ORFs) from various genomes into orthologous gene groups (OGs), thus allowing a pan-genomic search for T3Es. This enables the inclusion of genomes with insufficient known T3E data in a given study, provided that other genomes in the analysis have T3E data to compensate. Second, Effectidor II offers a more precise classification of T3SS components into distinct subtypes and homologous flagella, as well as associated chaperones. Third, an improved protein language model (pLM) of the secretion signal is provided. Fourth, we improved the search for T3E regulatory elements, and introduced a novel informative feature, which is the distance to the closest mobile genetic element component. Finally, we also provide a stand-alone version of Effectidor II.

## 2 Materials and methods

### 2.1 Input

The main input for Effectidor II is a set of files in FASTA format, one file for each genome. Each file should include all the ORF sequences in that genome. The following additional files are optional inputs, one file for each genome analyzed: (i) the full genomic sequence in FASTA format. This file may include plasmids and several contigs; (ii) the GFF annotation file. For the entire pan-genome analysis, the following inputs may be provided for homology searches: (i) files for the host proteome(s); (ii) proteomes of related bacteria that do not encode a T3SS.

### 2.2 OG detection

The ML training and prediction of Effectidor II are conducted on the OGs rather than the ORF level, and therefore its first step is OG detection. To find the OGs across the set of genomes, we use Microbializer (Avram *et al.* 2019), in which the ORFs of the different genomes are clustered into OGs, including paralogs. Orphan genes, i.e. ORFs without orthologs, are OGs with a single member. The default parameters used for OG detection are 50% identity and 60% coverage, as used for effector family classification (Costa *et al.* 2024); these parameters can be modified by the user.

### 2.3 Establishing the positive and negative OG samples

We first define the ORFs that encode T3Es and non-T3Es in each genome, similar to the way this was done in Effectidor (Wagner *et al.* 2022b). Briefly, the lists of known T3Es for each genome can be supplied as input by the user. Alternatively, they are automatically inferred by the program, based on sequence similarity (60% identity and *E*-value < 10^–10^) to a dataset of T3Es originally developed for Effectidor. This database is updated regularly and is provided as part of the web server (https://effectidor.tau.ac.il/data.html). Non-T3Es are defined among the remaining ORFs, based on sequence similarity (*E*-value < 10^–6^) to proteins of *Escherichia coli* K12, which does not encode for a T3SS. In Effectidor II, sequence similarity searches are conducted using MMseq2 (Steinegger and Söding 2017) to reduce running time compared to the previous searches that were based on BLASTp.

In previous ML models for identifying T3Es, the labeled data were single ORFs, that were T3Es (a positive label) or not (negative label). Here, instead, the ML models are trained on labeled OGs, i.e. each OG either represents T3E or non-T3E. To classify OGs as positive or negative, we apply the following rules: (i) if one or more of the ORFs within an OG are labeled as T3Es, the OG is labeled as positive; (ii) among the remaining OGs, if one or more of the ORFs are labeled as non-T3Es, the OG is labeled as negative; (iii) all other OGs are unlabeled, i.e. the trained algorithm will determine whether or not they are positive.

### 2.4 Identifying the T3SS components and associated chaperone genes

Detection and classification of T3SS components, which mediate the secretion of T3Es, is of high interest. Nevertheless, these components are excluded from the ML pipeline as they do not represent T3Es while still sharing some of their traits. These traits include regulation of their expression and—for a few of them—even type III-dependent secretion (e.g. subunits of the Hrp pilus and the translocon). Thus, we report these components separately from the T3Es and the non-T3Es. In Effectidor II we report the subtypes of the T3SS cluster as well as the flagella. To do so, we established datasets for every T3SS subtype, later used for homology searches. In addition, we established a list of chaperone proteins that were previously shown to be associated with effector translocation. Similar to the T3SS components, we report the chaperones detected within the analyzed genomes. Full details regarding these datasets and the conducted homology searches are available in the Supplementary Data.

### 2.5 Feature extraction per OG and ML model

In Effectidor II, features are computed for each OG. For example, we compute the average protein length over all ORFs comprising the OG. Similarly, we compute features that characterize the G+C content of each OG, the amino acid composition, the proximity in the genome to known effector genes, and the presence of regulatory elements in the promoters. We also incorporated the distance (in base pairs) to the closest mobile genetic element component as a feature. The full list of features, how they are computed, and which inputs are required for their extraction are detailed in Supplementary Table S5. Model training including feature selection in cross-validation and model selection is done similarly to Effectidor I, except here, the final prediction scores reflect the likelihood of an OG, rather than of an individual ORF, to be positive, i.e. a T3E.

### 2.6 Regulatory motifs upstream to T3E genes

Effectidor II has five optional features representing the presence of different regulatory motifs that were previously shown to be associated with T3E regulation, e.g. the PIP-box in *Xanthomonas* (Koebnik *et al.* 2006), the hrp-box in *Pseudomonas syringae* (Shen and Keen 1993, Zwiesler-Vollick *et al.* 2002), and the tts-box in Rhizobia (Krause *et al.* 2002). Some modifications were introduced to the way regulatory motifs are searched for. For example, the length of the promoter region considered was increased from 350 bp to 700–1000 bp upstream of the predicted start codon, and the exact definitions of some of the motifs were also modified. The resulting modifications to the search for regulatory motifs and analyses that led to these modifications are detailed in the Supplementary Data.

### 2.7 Outputs

The main output is a downloadable Excel file with the OGs’ information, sorted in descending order by their likelihood to represent T3Es according to the ML predictions, their annotations derived from the input files, and the ORFs’ locus tags from each genome. ORFs indicated as pseudogenes in the input GFF files are indicated as such in this output file. The ten best-scoring unlabeled OGs and the positive samples, together with their prediction scores, are visually provided as output in two tables. For each OG in these tables, we provide the identity of their closest known effector homolog(s) within the T3Es database of Effectidor, when available. A separate CSV file listing T3SS or flagella components and a CSV file listing chaperones found in the input genomes are also available for download.

Phyletic patterns (the presence–absence matrix of OGs across the analyzed genomes) of the T3SS components and known and predicted T3Es are supplied as both textual and graphical outputs. Additional graphical outputs regarding the features include a bar plot presenting the top ten features and their contribution to the learning, and comparisons of each of these features between T3Es and non-T3Es samples in violin plots (these outputs were already provided in the first version of Effectidor).

### 2.8 Secretion signal

We have previously applied a pLM to characterize the secretion signal of T3Es (Wagner *et al.* 2022a). Here, we used an updated and curated data for training and evaluation, and tested several alternative pre-trained pLMs for their ability to accurately classify ORFs as effectors versus non-effectors based on their 100 N-terminal amino acids. The following models were tested: ESM-2 (Lin *et al.* 2023), ProtT5 (Elnaggar *et al.* 2022), and ProteinBert (Brandes *et al.* 2022). For each pre-trained model, its embeddings for the train and test data were extracted, and a classifier was trained on the train data to differentiate the effectors from the non-effectors. The positive train and test examples were known T3Es from the curated Effectidor database, and the negative train and test examples were all protein sequences of *E. coli* K12, which do not show sequence similarity to T3Es from the curated database. Our analysis showed that ProtT5 embeddings combined with a multi-layer perceptron classifier achieved the best results (see Supplementary Data), hence in Effectidor II we replaced the secretion signal feature with the output of this new improved model.

### 2.9 Experimental validation of a putative T3E

To test whether the predicted T3E is indeed secreted by the T3SS, we used a reporter fusion approach as described before (Zhao *et al.* 2013), except here we used a plasmid of the pBBR1MCS series instead of the original pLAFR plasmid backbone (Kovach *et al.* 1995). Briefly, a DNA fragment containing 34 bp upstream of the translational start codon and the first 71 codons of the candidate T3E gene was synthesized and cloned in front of a truncated avrBs1 gene lacking the first 58 codons encoding its endogenous type III secretion signal (GenScript Biotech B.V., Rijswijk, Netherlands). Notably, the corresponding AvrBs1 domain is still able to trigger a hypersensitive response in plants carrying the corresponding resistance gene *Bs1*. This plasmid was then introduced into two derivatives of the *Xanthomonas campestris* pv. campestris strain 8004; one containing a knockout mutation of its endogenous *avrBs1* gene and the other a knockout mutation of the T3SS gene *hrcV*, thus preventing the secretion of T3Es. Derived strains and appropriate controls were inoculated into six to eight weeks old pepper plants of the cultivar ECW-10R and symptom formation was followed over a period of seven days.

## 3 Results

To demonstrate the capabilities of Effectidor II, we searched for novel T3E genes in *Xanthomonas euroxanthea* CPBF 424. This pathogenic bacterium was first isolated from infected walnut trees in Portugal in 2016 (Martins *et al.* 2020). Based on sequence similarity with previously proven T3Es, eleven genes encoding for T3Es were found by Effectidor I, i.e. the version that does not consider pan-genomes. All other ORFs received scores <0.5, meaning that Effectidor I did not predict any novel T3Es in this genome with the exception of a pseudogene. We next ran Effectidor II on an *X. euroxanthea* pan-genome that included eight genome sequences: seven *X. euroxanthea* genomes and an outgroup, *Xanthomonas hortorum* pv. pelargonii 305 (Xhp305) (Wagner *et al.* 2023). A total of 47 T3E-OGs were discovered in the pan-genome. The majority, 35, were considered true T3E-OGs by Effectidor II based on sequence similarity of at least one member of the OG to a known T3E. Effectidor II also predicted twelve putative novel T3E-OGs. Of the 47 T3E-OGs, ten are core (i.e. found in all genomes), and 37 are accessory (Supplementary Fig. S6). Projecting the results onto the genome sequence of CPBF 424, no additional T3E was identified based on sequence similarity to the database of known effectors. Nevertheless, four OGs with members in CPBF 424 were predicted with a score >0.4 (Table 1). Of note, these predictions with the exception of a pseudogene, were not observed when the outgroup genome was not included, emphasizing the importance of extracting relevant information from genomic sequences of related, yet diverse, bacterial strains (see Supplementary Information for details).

**Table 1.** Novel predicted T3Es in CPBF 424.^a^

Locus in CPBF 424	Annotation	Single genome	Pan-genome
XTG_RS16970 (pseudogene)	Pectate lyase	0.593	0.608
XTG_RS02340	Hypothetical protein	0.286	0.465
XTG_RS10080	Hypothetical protein	0.438	0.428
XTG_RS02345	Hpa3 family type III secretion system protein	0.35	0.403

a Single genome: scores were computed based on a single genome. Pan-genome: scores were computed based on 7 genomes of *X. euroxanthea* and the outgroup genome, Xhp305.

The putative novel T3E XTG_RS02340 has no clear sequence similarity to any known T3E in our effector database. When searched against NIH’s nr database, only hypothetical proteins were found as homologs. The secretion signal score of the OG harboring this T3E was 0.98, substantially higher than the score for non-effector OGs, with a mean of 0.075 and a standard deviation of 0.163. The PIP-box regulatory element was found in the upstream region of some, but not all, members of this OG. Specifically, the PIP-box motif was not recognized in CPBF 424. Inspection of the alignment of this gene, including its regulatory region across the multiple genomes revealed that the reason this PIP-box motif was not recognized is likely due to a wrong annotation of the start codon (see Supplementary Information). Of note, the high score of the secretion signal, despite this mis-annotation, stems from the fact that the score is the maximum over all members comprising the OG (Supplementary Table S5). This result further demonstrates the benefit of combining data from multiple genomes within a single run. In addition, this OG presented a very low G + C content compared to other ORFs within the analyzed genomes (average Z-score of −2.8). We next experimentally validated this putative T3E using the AvrBs1 reporter fusion approach (Supplementary Fig. S7). When the first 71 amino acids of XTG_RS02340 were fused to the hypersensitive response (HR)-inducing domain of the T3E AvrBs1, an HR was observed in *Bs1*-expressing pepper leaves when the chimeric protein was expressed in a *Xanthomonas* strain with an intact secretion system. In contrast, an HR was not observed when the secretion system was defective due to a knockout mutation in its conserved component *hrcV*. This demonstrates that the T3SS was responsible for the export of the chimeric protein and that the N-terminal region of XTG_RS02340 was sufficient for secretion of the AvrBs1 reporter moiety.

## 4 Discussion

We present Effectidor II, a web server for predicting and analyzing T3E arsenals in pan-genomes. To our knowledge, it is the first tool that allows the identification and prediction of T3Es at a pan-genomic level. It opens the door for broader analyses that include among others, the identification of core and specialized effectors among a group of bacteria, and the study of gain and loss dynamics across their phylogeny. Using the newly developed web server, we predicted and experimentally validated a novel T3E in *Xanthomonas*, named XopBH, which could not be identified using the previous version of the web server. Finally, Effectidor II provides means to explore the evolution of T3SS components (and flagellar genes) across pan-genomes.

## Supplementary Material

btaf272_Supplementary_Data
