# Supplementary material for: Effectidor II: a pan-genomic AI-based algorithm for the prediction of type III secretion system effectors
Source: Bioinformatics. 2025 Apr 29;41(5):btaf272. doi: 10.1093/bioinformatics/btaf272 (PMC12085239; doi:10.1093/bioinformatics/btaf272)
Supplement: btaf272_Supplementary_Data [file btaf272_supplementary_data.docx]

**Effectidor II: A pan-genomic AI-based algorithm for the prediction of type III secretion system effectors**

**SUPPLEMENTARY DATA**

Naama Wagner^1^, Ella Baumer^1^, Iris Lyubman^1^, Yair Shimony^1^, Noam Bracha^1^, Leonor Martins^2,3^, Neha Potnis^4^, Jeff H. Chang^5^, Doron Teper^6^, Ralf Koebnik^7^, and Tal Pupko^1^ †

1. The Shmunis School of Biomedicine and Cancer Research, George S. Wise Faculty of Life Sciences, Tel Aviv University, Tel Aviv 69978, Israel

2. CIBIO - Centro de Investigação em Biodiversidade e Recursos Genéticos, InBIO - Laboratório Associado, Universidade do Porto, Vairão, Portugal

3. FCUP - Faculdade de Ciências, Departamento de Biologia, Universidade do Porto, Porto, Portugal

4. Department of Entomology and Plant Pathology, Auburn University, Auburn, AL, USA

5. Department of Botany and Plant Pathology, Oregon State University, Corvallis, Oregon, USA

6. Department of Plant Pathology and Weed Research, Institute of Plant Protection Agricultural Research Organization (ARO), Volcani Center, Rishon LeZion, Israel

7. Plant Health Institute of Montpellier, University of Montpellier, CIRAD, INRAe, Institut Agro, IRD, Montpellier, France

† To whom correspondence should be addressed:

Tal Pupko, Tel: +972 3 6407693; E-mail: talp@tauex.tau.ac.il

Keywords: Type III secretion system, Effector proteins, Machine learning, Effectidor, Pan-genome analysis, *Xanthomonas euroxanthea*

**Identifying the T3SS components**

In Effectidor II we report the subtypes of the T3SS cluster along with the flagella, separately from the T3E prediction. To achieve this, we created datasets for each T3SS subtype and an additional dataset for the flagella, to be used for homology searches. To establish the datasets of the different T3SS subtypes we relied on Table 1 from Gazi *et al.* (2012). We used one or two representative bacterial genomes that possess each T3SS subtype. The flagella dataset was created using the genome of *Salmonella enterica* and includes the following components: flgB, flgC, flgE, flgF, flgG, flgH, flgI, flgK, flgL, flhA, flhB, motB, motA, fliY, fliC, fliD, fliE, fliF, fliG, fliH, fliI, fliJ, fliM, fliN, fliO, fliP, fliQ, and fliR. The T3SS subtypes include Chlamydiales, Hrp1, Hrp2, Rhc, SPI-1, SPI-2, and Ysc. The genomes used as representatives for each subtype and their included components are specified in Table S1.

The T3SS genes are searched for in the input bacterial genome(s) using MMseqs2 (Steinegger and Söding, 2017). We use the input bacterial genome as the target, conducting MMseqs2 searches using each of the T3SS subtypes as queries. The command we use is "mmseqs easy-search {query} {target} {output} {tmp_mmseqs}". For each query T3SS component, the best hit in the bacterial genome is considered if it has an E-value lower than 10^−10^ with query coverage higher than 30%. In case a certain protein in the input genome is found to be a hit matching our criteria for components of more than one T3SS subtype, for example, SctC of Hrp1 and SctC of Hrp2, the hit with the maximal bit score is kept.

| Chlamydiales | Hrp1 | | Hrp2 | Rhc | SPI-1 | | SPI-2 | | Ysc | **T3SS subtype** |
| --- | --- | --- | --- | --- | --- | --- | --- | --- | --- | --- |
| *C .trachomatis* | *P .syringae* | *P .agglomerans* | *X .campestris* | *B .elkanii* | *S .flexneri* | *S .enterica* | *S. enterica* | *E. coli* O111:H- | *P .aeruginosa* | **Bacteria** |
| V | V | X | V | V | V | V | V | V | V | **SctV SctQ** |
| V | X | V | V | X | V | V | V | V | V | **SctW** |
| V | V | X | V | V | V | V | V | V | V | **SctN**  **SctO** |
| V | X | V | V | X | V | V | V | X | V | **SctP** |
| V | V | X | V | V | V | V | V | V | V | **SctRSctS** |
| V | V | X | V | V | V | V | V | V | V | **SctT**  **SctU** |
| V | V | X | V | V | V | V | V | V | V | **SctC SctD** |
| V | V | X | V | V | V | V | V | V | V | **SctFSctJ** |
| X | V | X | V | V | V | V | V | V | V | **SctI** |
| X | V | X | X | X | V | V | X | X | V | **SctK** |
| V | V | X | V | V | V | X | V | V | V | **SctL** |
| V | X | X | X | X | V | V | V | V | V | **SctA** |
| V | V | X | X | X | V | V | V | V | V | **SctB** |
| V | X | X | V | V | V | V | V | V | V | **SctE** |

*Table S1: the T3SS components, listed using the unified nomenclature Sct (SeCreTion) prefix, included in each dataset, where ’V’ indicates the presence of a component in the dataset and 'X' indicates its absence.*

**Identifying chaperones associated with type III effectors**

In Effectidor II we also report the presence of chaperones associated with type III effectors. To this end, we first established a list of 55 chaperone proteins based on a literature survey. The complete list of these chaperone sequences is publicly available within the Effectidor-II webserver. The chaperones are searched for in the input bacterial genome(s) using MMseqs2 (Steinegger and Söding, 2017), with the same parameters used for searching the T3SS components.

**Regulatory elements analysis**

As one of Effectidor's (optional) features, we search for known regulatory elements associated with T3Es. Specifically, the regulatory elements included in Effectidor II are:

- **PIP-box** (Koebnik *et al.*, 2006; Mukaihara *et al.*, 2004) – found in bacterial genomes harboring Hrp2 T3SS such as *Xanthomonas*, *Ralstonia*, and *Acidovorax*. Its consensus sequence is TTCGBN_15_TTCGB.
- **Hrp-box** (Zwiesler-Vollick *et al.*, 2002) – found mainly in *Pseudomonas syringae.* Its consensus sequence is KGGARCYN_15-16_CCACN_2_A.
- **mxiE-box** (Mavris *et al.*, 2002; Bongrand *et al.*, 2012) – found in *Shigella.* Its consensus sequence is GTATCGT_7_ANAG.
- **Exs-box** (Brutinel *et al.*, 2009) – found in *Pseudomonas aeruginosa.* Its consensus sequence is A_5_NWNMYN_3_MYTGYA_2_K.
- **TTS-box** (Krause *et al.*, 2002) – found in *Rhizobia.* Its consensus sequence is GTCAGBTN_4_GWMAGBHN_3_B_2_N_4_A.

In the previous version of Effectidor, we searched for each regulatory element the exact match (as specified above) or a match with up to one mismatch in the promoter of each gene. The promoter considered was in the range of 350 nucleotides upstream of the start codon. In Effectidor II, we aimed to determine the optimal promoter length to be considered. To this end, we searched for the length that maximizes classification accuracy. We analyzed it separately for each regulatory element, computing the Matthews Correlation Coefficient (MCC) and F1 scores as a function of the promoter lengths. The genomes used for these analyses are listed in Table S2 and the results are shown in Figure S1 below.

Table S2: Regulatory elements and bacterial genomes used for the promoter analysis

| **Element** | **Bacterial genomes used for the analysis** |
| --- | --- |
| PIP-box | *Xanthomonas hortorum* pv. pelargonii str. 305*, X. fragariae* str. Fap21*, Ralstonia pseudosolanacearum* str. GMI1000, and *Acidovorax citrulli* str. M6 |
| Hrp-box | *Pseudomonas syringae* str. Susan2139 |
| mxiE-box | *Shigella flexneri* |
| Exs-box | *Pseudomonas aeruginosa*, *Aeromonas salmonicida*, and *Yersinia enterocolitica* |
| TTS-box | *Bradyrhizobium japonicum* and *B. arachidis* |

Overall these results show the promoter considered should be longer than the 350 bp used in Effectidor I. Following these findings, we have increased the considered length to 700 bp


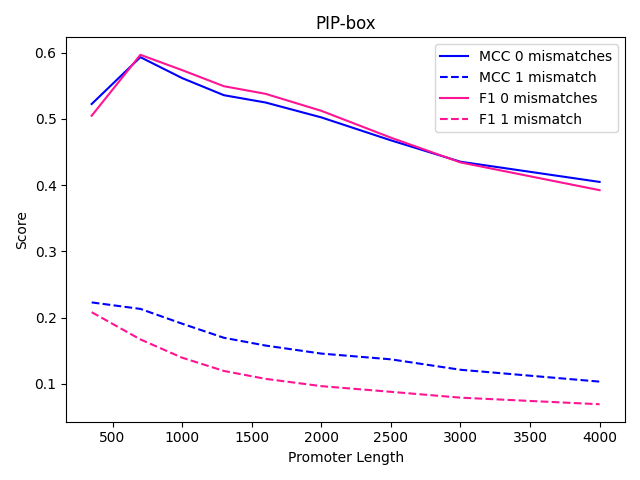

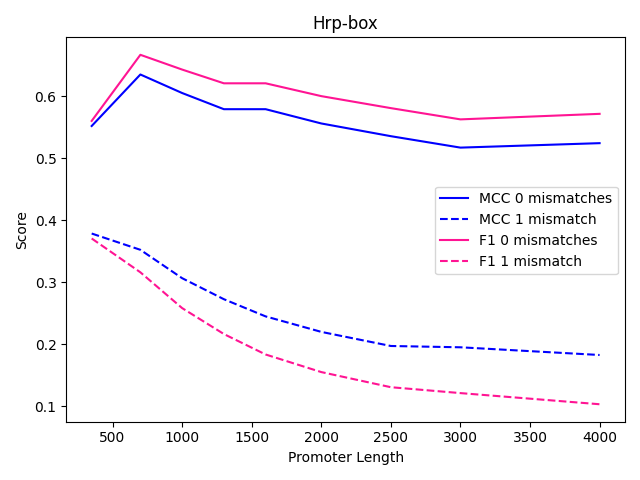

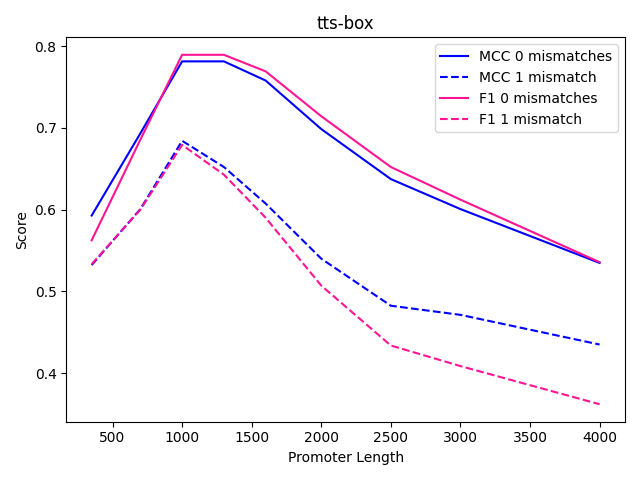

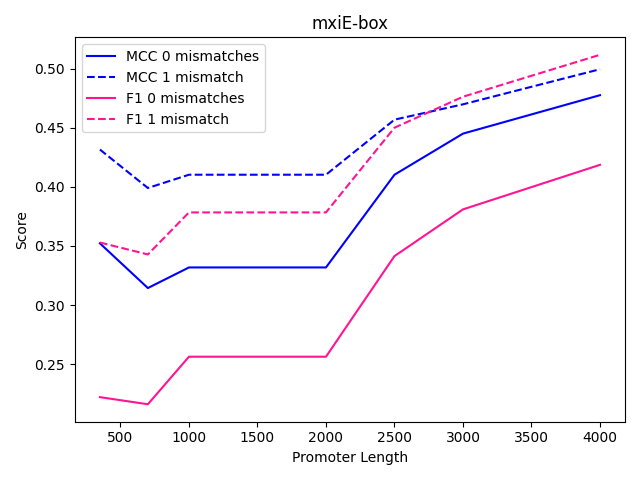


Figure S1: MCC and F1 classification scores based on the existence of regulatory elements in the promoter, as a function of the promoter length.

upstream of the start codon, with the exemption of the TTS-box, for which we consider 1,000 bp upstream of the start codon. In addition, the classification scores achieved using the exact match were generally higher than the ones achieved considering up to one mismatch (Figure S1), and we thus decided to only consider exact matches.

An exception to this rule was the mxiE-box, for which the motif with up to one mismatch showed better accuracy and kept increasing with the increase in promoter length, and the Exs-box that we could not identify in the reported genomes using the abovementioned motif. We therefore further studied the consensus sequences of these elements. For the reconstruction of the Exs-box motif, we relied on the results presented in Figure S4 of Burstein *et al*. (2015) and for the reconstruction of the mxiE-box motif, we relied on the results presented in Figure 2 of *Mavris et al*. (2002). We repeated the search for both motifs. The resulting consensus sequences, computed using MEME (Bailey *et al*., 2006) are available in Figures S2.


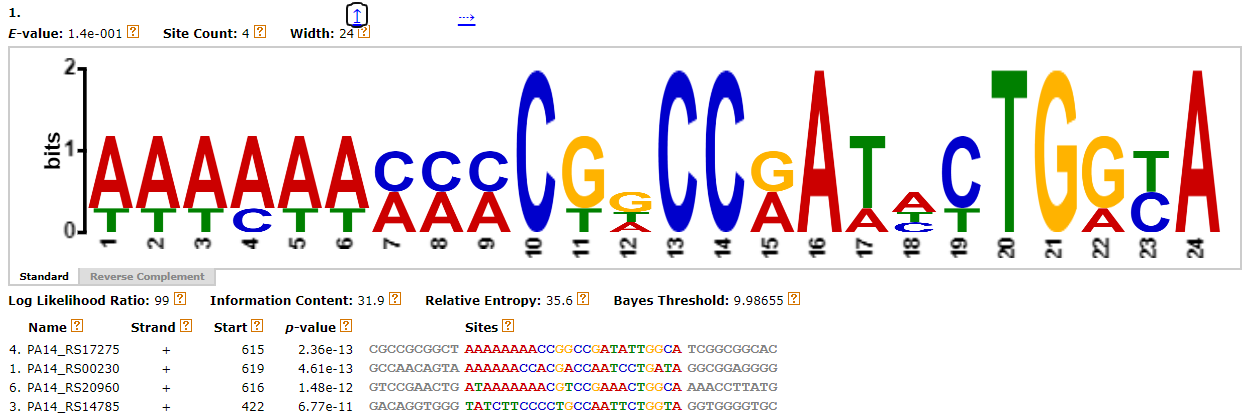


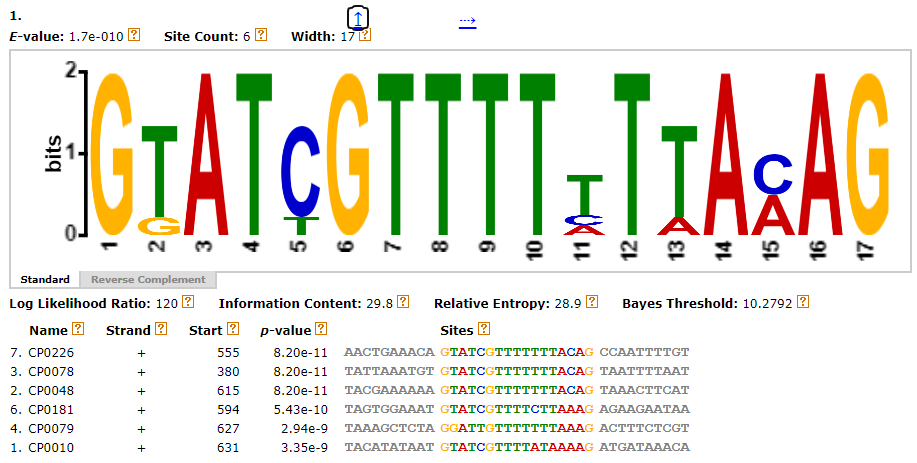


Figure S2: New Exs-box (above) and mxiE-box (below) consensus sequence.

We then repeated the analysis of the promoter length for both regulatory elements, and the results are presented in Figure S3.

Figure S3: MCC and F1 classification scores based on the existence of updated Exs and mxiE regulatory elements in the promoter, as a function of the promoter length.


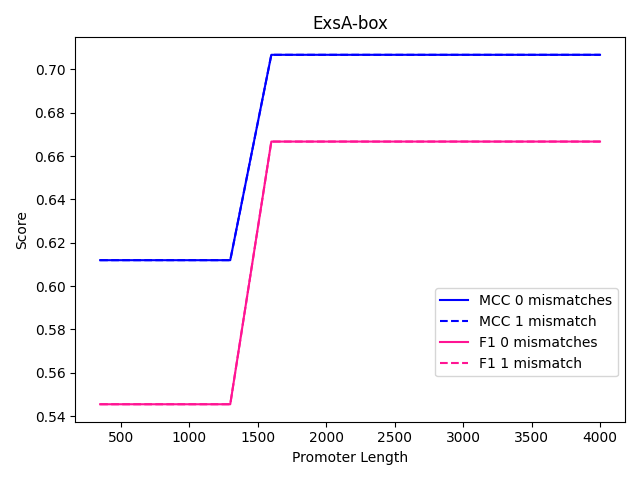

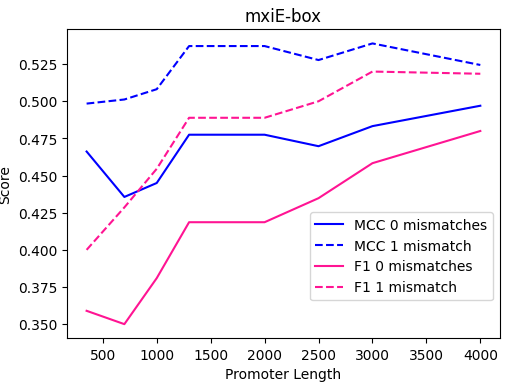


While these results show an increase in classification scores with the increase in the promoter length, we have limited it to 700 for both motifs. The final consensus sequences and promoter length considered for each element in Effectidor II are summarized in Table S3.

Table S3: Regulatory elements implemented in Effectidor II

| **Element name** | **Consensus sequence** | **Considered promoter length** |
| --- | --- | --- |
| **PIP-box** | TTCGBN_15_TTCGB | 700 |
| **Hrp-box** | KGGARCYN_15-16_CCACN_2_A | 700 |
| **mxiE-box** | GKATYGT_4_HTWANAG | 700 |
| **Exs-box** | W_3_MW_2_M_3_CKDCCRAWHYTGRYA | 700 |
| **TTS-box** | GTCAGBTN_4_GWMAGBHN_3_B_2_N_4_A | 1,000 |

**Distance to closest mobile element component**

Effectidor II calculates the distance, in base pairs, of a given ORF from the closest mobile genetic element (MGE) component. This addition is based on the observation that some T3Es are located within genomic regions associated with MGEs, such as bacteriophages or transposons (McCann and Guttman, 2008; Orfei *et al*., 2023). The location of the MGE components, which include transposases, integrases, and non-bacterial recombinases, are extracted from the annotated GFF3 file. We denote this feature as MGE proximity.

**Secretion signal**

The N-terminal region of T3Es was previously shown to harbor a secretion signal, which is recognized by the T3SS. This signal has high sequence variability among different T3Es and hence cannot be detected with classic sequence-similarity search methods such as Blast. We previously modeled this secretion signal using a protein language model (pLM) and added a feature to the machine learning pipeline of Effectidor, which quantifies the strength of this secretion signal (Wagner *et al.*, 2022). In this work, we revised and improved this feature, which is based on the 100 N-terminal amino acids. The positive data we used for training and testing are the updated and curated protein sequences from the Effectidor database, and the negative data are all protein sequences of *E.coli* K12, except for those that showed sequence homology to positive sequences. As a pre-processing step, we clustered all positive sequences and all negative sequences using Mmseqs2 easy-cluster command with an E-value threshold of 1e-4. Thus, we removed redundancy from our dataset such that any two sequences in the final datasets do not show sequence similarity. We then split our positive and negative samples (with stratification with respect to the label) to 80% train and 20% test sets. We examined the following pre-trained pLMs: ProtT5 (Elnaggar *et al.*, 2022), ProteinBert (Brandes *et al.*, 2022), and several sizes of ESM-2 (Lin *et al.*, 2023) – 8M, 35M, 150M, 650M and 3B parameters. For each pre-trained model, we extracted the embeddings of the train data and used them to train binary classifiers that differentiate effectors from non-effectors. The following classifiers were examined: k-nearest neighbors, logistic regression, random forest, gradient boosting and multi-layer perceptron. The hyper-parameters for each classifier were fine-tuned using 5-fold cross validation, and the best classifier model and configuration was chosen as the one that maximized the mean MCC (Matthews correlation coefficient) on the held-out sets. The hyperparameters grid search was as detailed in Table S4.

Table S4: Hyperparameters grid search for the binary classifiers used on the pLM embeddings

| **Model** | **Hyperparameter** | **Values** |
| --- | --- | --- |
| K-nearest neighbors | n_neighbors | [5, 10] |
|  | weights | ['uniform', 'distance'] |
|  | algorithm | ['ball_tree', 'kd_tree', 'brute'] |
|  | leaf_size | [15, 30] |
|  | p | [1, 2] |
| Logisitic regression | solver | ['liblinear'] |
|  | penalty | ['l2'] |
|  | C | [0.001, 0.01, 0.1, 1, 10, 100, 1000] |
|  | max_iter | [500] |
|  | class_weight | ['balanced', None] |
| Random forest | n_estimators | [20] |
|  | criterion | ['gini', 'entropy'] |
|  | max_features | ['sqrt', 'log2'] |
|  | min_samples_split | [2, 5, 10] |
|  | min_samples_leaf | [1, 4] |
|  | class_weight | ['balanced', None] |
| Gradient boosting | learning_rate | [0.05, 0.1] |
|  | n_estimators | [10, 50, 200] |
|  | max_depth | [3, 5, 10] |
|  | subsample | [0.6, 1] |
| Multi-layer perceptron | hidden_layer_sizes | [(10,3),(30,5),(50,10), (100,)] |
|  | activation | ['tanh', 'relu'] |
|  | solver | ['adam'] |
|  | alpha | [0.0001, 0.05] |
|  | learning_rate | ['constant', 'adaptive'] |
|  | max_iter | [400] |
|  | early_stopping | [True] |

We also fine-tuned the pre-trained models to our data. Specifically, we randomly split the train set to 75% train and 25% validation. We trained for ten epochs and recorded the model weights after each epoch, which is termed a checkpoint. Finally, among the 10 checkpoints, we selected the one that maximizes the MCC metric on the validation set.

The code of the secretion signal analysis is available at <https://github.com/shimooper/T3Es_secretion_signal_prediction>. The following packages were used for the ESM-2 and ProtT5 models: python 3.9.19, pytorch 2.3.1, pytorch-cuda 11.8, biopython 1.84, scikit-learn 1.5.1, wandb 0.17.5, transformers 4.43.3, sentencepiece 0.2.0. The following packages were used for the ProteinBert model: python 3.8.19, tensorflow 2.4.0, tensorflow_addons 0.21.0, biopython 1.83, scikit-learn 1.3.2.

Figure S4 shows the performance of all the trained models on our test set. The pre-trained ProtT5 embedding with fine-tuning was the most accurate according to MCC and the area under the precision-recall curve (AUPRC) metrics on the test set. Nevertheless, the inference time of the fine-tuned model was significantly higher than the inference time of the original model, and the gain in accuracy was small. Hence, we chose to use the original pre-trained model with a classifier head, which was chosen to be a multi-layer perceptron.

Note that in the previous version of Effectidor, we combined pre-trained embeddings of a pLM with Hobbs features (Hobbs *et al.*, 2016) to represent a sequence, and trained a classifier on this combined set of features. Here, using more powerful pLMs, we achieved better results on our test sets using the pre-trained embeddings alone without including Hobbs's features.


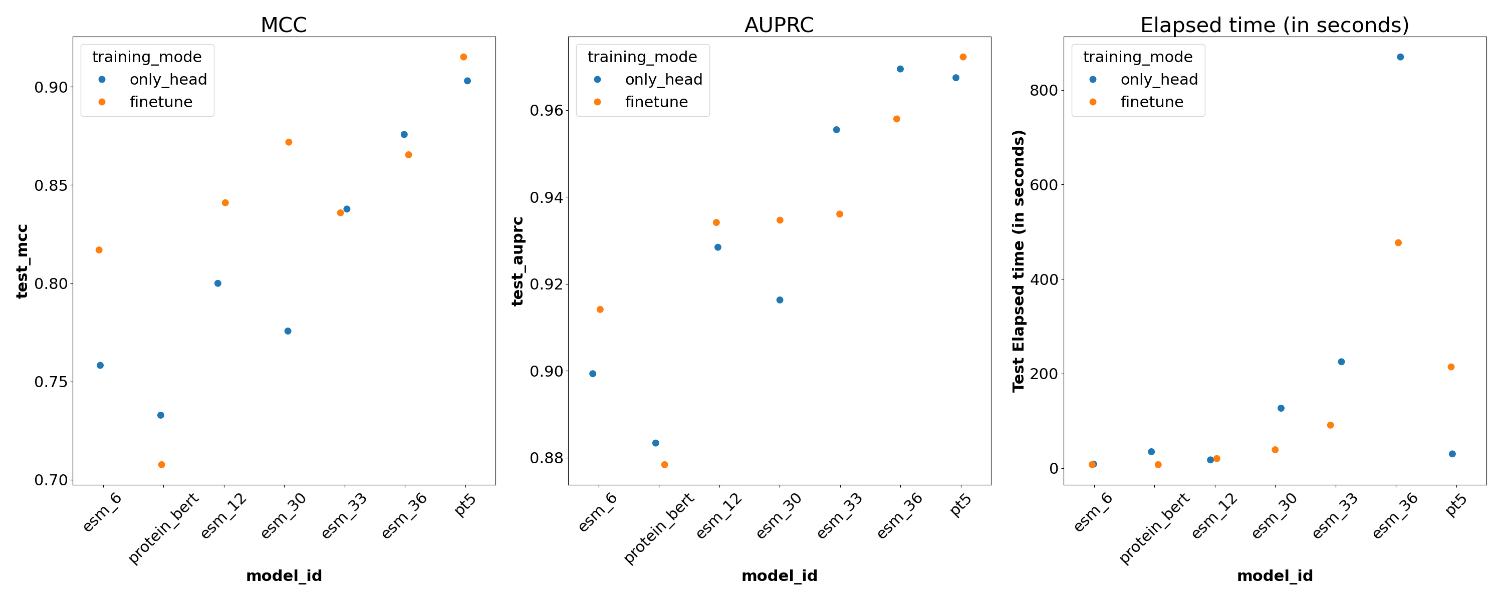


Figure S4: Performance (MCC and AUPRC) of the tested pLMs on the test dataset.

**Features used in Effectidor II**

The full list of features available in Effectidor II, along with their required input, is available in Table S5.

Table S5: Features used in Effectidor II and their adaptation to the transition to OGs-oriented learning.
The Input column specifies the required input for optional features.

| **Feature** | **Description** | **Adaptation to OG** | **Input** |
| --- | --- | --- | --- |
| Amino acid frequencies | The relative frequency of each amino acid across the full protein sequence, and in the N-terminal region (40 features). | Mean per OG |  |
| Amino acid properties | Properties such as hydrophobicity, hydrophilicity, and amphiphilicity of the N-terminal region using AA-index (Kawashima *et al.*, 2008) and amino acid profile in this region (4 features). | Mean per OG |  |
| Sequence similarity | Sequence similarity search using MMseqs2 (Steinegger and Söding, 2017) against various databases (i.e., T3Es, eukaryotic host proteome if given, and related bacterial proteomes lacking the T3SS if given). The feature taken for each database is the best bit-score. | Mean per OG | Datasets for searching (host, closely related bacteria lacking the T3SS) |
| GC content | The relative abundance of Guanine and Cytosine. | First, this feature is normalized per genome. On the normalized values, the mean per OG is computed |  |
| Protein length |  | Mean per OG |  |
| Type III secretion signal (optional) | A prediction of the presence of a type III secretion signal in the N-terminal region (Wagner *et al.*, 2022). A novel pLM was used to characterize the signal in Effectidor II. | Maximum per OG |  |
| Distance from closest effector | Number of ORFs between a given ORF to a known effector on the genome. | Median per OG | GFF3 file(s) |
| # of effectors in K nearest neighbors | Number of known effectors in proximity to the ORF on the genome.  Computed for $k\in\{5,10,15,20,25,30\}$. | Mean per OG | GFF3 file(s) |
| Regulatory elements | A binary feature representing the presence of regulatory elements in the promoter of the ORF. | Maximum per OG | GFF3 and full genome sequences files, and choosing the desired regulatory elements |
| Similarity to effectors vs. non-effectors | The mean amino acid profile of the known effectors is computed, and a similar mean profile is calculated for the known non-effectors. The Euclidean distance between the amino acid profile of a given ORF and the mean profile of effectors is computed, and a similar distance is calculated to the mean profile of non-effectors. The difference between these two distances is taken for this feature. Values lower than 0 represent amino-acid profiles closer to that of T3Es than non-T3Es, and vice versa. | First, the mean amino acid profile is computed per OG, and then the process is conducted as in the description, on OGs instead of ORFs |  |
| MGE proximity | The distance in bp between a given ORF to the closest annotated MGE component. | Median per OG | GFF3 file(s) |

**Example run**

For an example run we aimed to identify potential novel T3Es in *Xanthomonas euroxanthea* CPBF 424 (accession number GCF_905187425). We first ran Effectidor as in the old version, on this genome alone. In this run, eleven genes were identified as T3Es based on homology to Effectidor's T3Es dataset, and no additional genes were predicted as T3Es. We then repeated the analysis, this time including all 18 genomes of *X. euroxanthea*. Specifically, for this run, data of the following genomes were downloaded from NCBI: GCF_002940465, GCF_003992965, GCF_003993495, GCF_003993515, GCF_003993525, GCF_011761665, GCF_011761715, GCF_011761845, GCF_011927445, GCF_014195725, GCF_014195735, GCF_014199845, GCF_900537245, GCF_903989455, GCF_903989465, GCF_905187425, GCF_905367725, GCF_905367735.


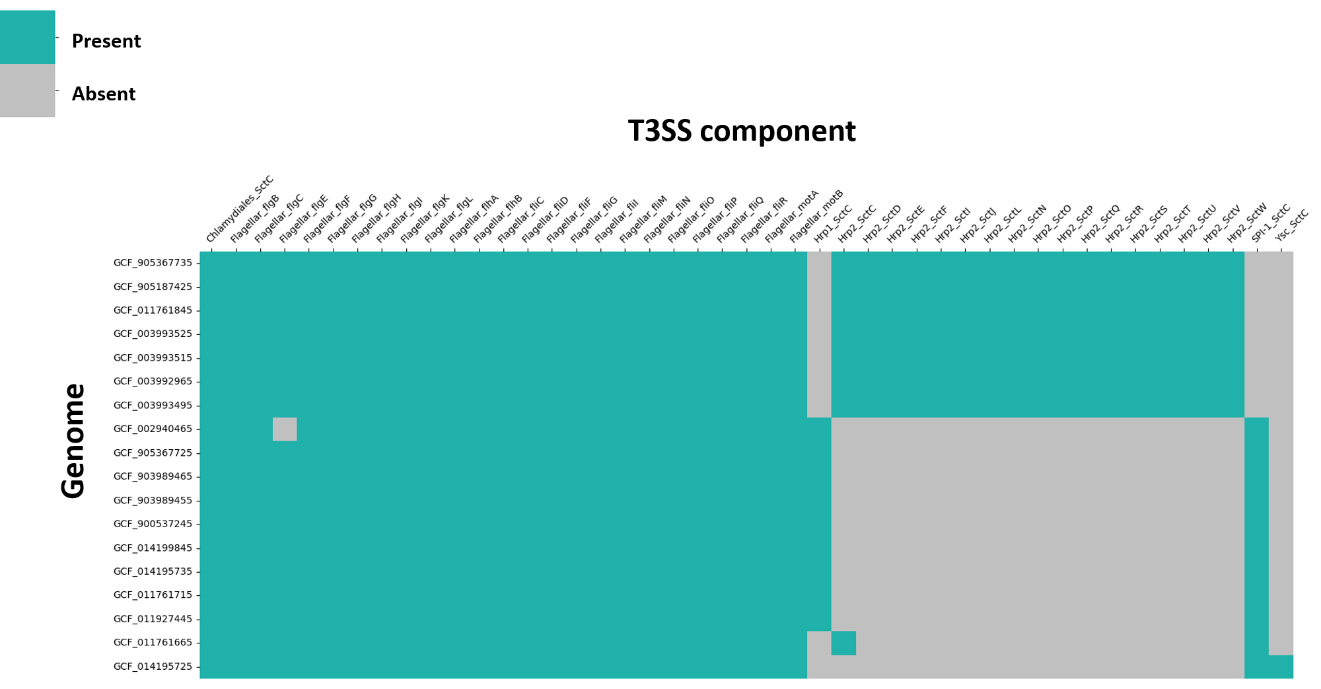
In this run, we noticed that many of the *X. euroxanthea* genomes do not encode a T3SS (Figure S5); thus, we re-ran it, including only the genomes that encode a full hrp2 T3SS. In this run, the lowest scoring identified T3E scored 0.447. However, no additional OG with a member from CPBF 424 scored above 0.4. We therefore repeated this analysis a third time, adding the genome of *X. hortorum* pv. pelargonii 305 (Wagner *et al.*, 2023). For this genome, we used our annotation, as available in the supplementary data of Wagner *et al.* (2023).

Figure S5: Presence/absence map of the T3SS and flagella components across all X. euroxanthea genomes, as outputted by Effectidor II.

In this final run, a total of 47 T3E OGs were discovered in the pan-genome. The majority, 35, were considered true T3E OGs by Effectidor II based on sequence similarity of at least one member of the OG to a known T3E. Effectidor II also predicted 12 putative novel T3E OGs. Of the 47 T3E OGs, ten are core (i.e., found in all genomes), and 37 are accessory (Figure S6).


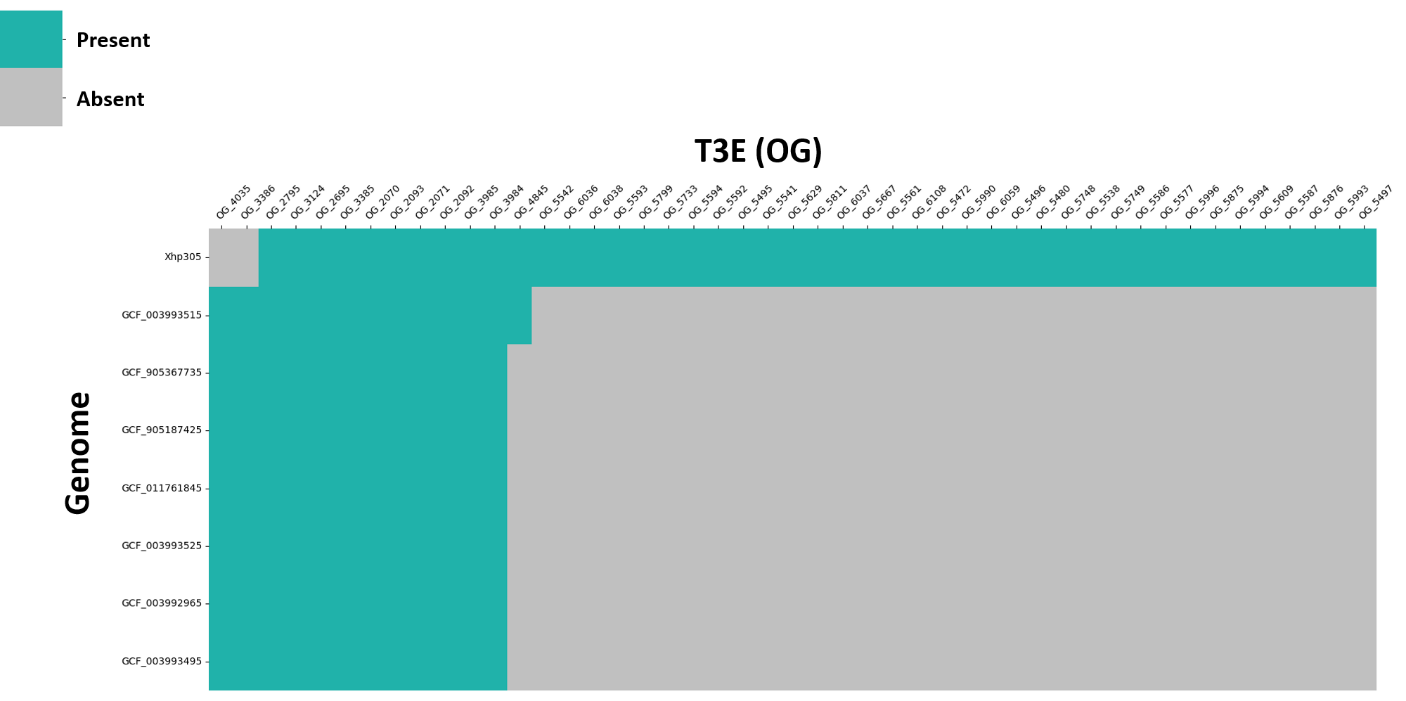


Figure S6: Presence/absence map of the T3Es identified by Effectidor II across all analyzed genomes.

Following the inclusion of Xhp305, the lowest scoring T3E scored 0.477, and four OGs with members in CPBF 424 scored higher than 0.4. Of note, 0.4 was chosen as a cutoff for T3E candidates instead of the natural 0.5 for two reasons: (1) a positive labeled sample scored below 0.5; (2) the only unlabeled OG with a member within CPBF 424 that scored above 0.5 represented a pseudogene (Table S6). The prediction comparison of these four T3E candidates between all three runs is available in Table S6.

Table S6: Predicted T3Es in X. euroxanthea CPBF 424 in three runs of Effectidor. Effectidor I: run with the genome of interest alone; Effectidor II without an outgroup: a pangenomic analysis with the seven X. euroxanthea genomes that encode a T3SS; Effectidor II with an outgroup: a pangenome analysis with the seven X. euroxanthea genomes that encode a T3SS and the genome of Xhp305.

| **Locus in** **CPBF 424** | **Annotation** | **Effectidor I** | **Effectidor II without an outgroup** | **Effectidor II with an outgroup** |
| --- | --- | --- | --- | --- |
| XTG_RS16970 (pseudogene) | pectate lyase | 0.593 | 0.262 | 0.608 |
| XTG_RS02340 | hypothetical protein | 0.286 | 0.234 | 0.465 |
| XTG_RS10080 | hypothetical protein | 0.438 | 0.307 | 0.428 |
| XTG_RS02345 | Hpa3 family type III secretion system protein | 0.35 | 0.22 | 0.403 |

Of note, to test the impact of the MGE proximity feature, we repeated the three runs presented in Table S6, excluding this feature, which resulted in mostly lower scores (Table S7).

Table S7: Predicted T3Es in X. euroxanthea CPBF 424 in three runs of Effectidor without the MGE proximity feature. Effectidor I: run with the genome of interest alone; Effectidor II without an outgroup: a pangenomic analysis with 18 X. euroxanthea genomes; Effectidor II with an outgroup: a pangenome analysis with 18 X. euroxanthea genomes and the genome of Xhp305.

| **Locus in CPBF 424** | **Effectidor I** | **Effectidor II without an outgroup** | **Effectidor II with an outgroup** |
| --- | --- | --- | --- |
| XTG_RS16970 (pseudogene) | 0.404 | 0.067 | 0.577 |
| XTG_RS02340 | 0.131 | 0.104 | 0.516 |
| XTG_RS10080 | 0.086 | 0.096 | 0.589 |
| XTG_RS02345 | 0.179 | 0.106 | 0.472 |

The top-scoring candidate, XTG_RS16970, is a pseudogene. We therefore examined the second-best candidate - XTG_RS02340. The PIP-box regulatory element was found in the upstream region of some, but not all, members of the OG harboring XTG_RS02340. Specifically, the PIP-box motif was not recognized in the predicted promoter of XTG_RS02340, as annotated in the genome sequence downloaded from NCBI that was used for this analysis. Inspection of the alignment of this gene area (see below), including its upstream and downstream regions, across multiple *Xanthomonas* genomes (including members of *X. euroxanthea*, *X. arboricola*, *X. hortorum*, and *X. hydrangea*), revealed that CPBF 424 does contain a perfect PIP-box, but 49 bp downstream of its predicted start codon. An alternative start codon was found downstream of this PIP-box, precisely 95 bp downstream of the -10 motif, and this alternative start codon was considered for the cloning of this gene for a translocation assay.

Examining additional features revealed that its GC content is very low compared to the rest of the ORFs in the genome (Z-score of -2.8 for the OG, and -2.7 for the specific gene), it resides in proximity to other effectors, its amino acid profile is closer to that of effectors versus non-effectors, and its predicted type III secretion signal is very strong (0.98 for the OG compared to the annotated gene, for which the predicted signal score was 0.91). In addition, it showed no sequence similarity to known effectors in our database (which lowered its prediction score, considering this is the most informative feature) nor to genes of closely related genomes without a T3SS (which increases the chances this may indeed be a T3E).

Following these predictions and PIP-box analysis, we validated that XTG_RS02340 is indeed translocated via the T3SS using a translocation assay. Specifically, the XTG_RS02340 N-terminal secretion signal-coding segment, considering the alternative start codon (see above) was cloned into pBBR1MCS-3 vector containing an N-terminally truncated version of the avrBs1 gene and introduced into Xcc 8004∆avrBs1 and Xcc 8004∆hrcV strains. Pepper plants of ECW cultivar and its resistant derivative, ECW-10R, were grown for 6 weeks, and inoculated with the Xcc8004∆hrcV and Xcc8004∆avrBs1 complemented with the 6 constructs individually. Complementation with pBBR1MCS-3::2017-∆avrBs1, pBBR1MCS-3::2017-∆avrBs1_STOP, pBBR1MCS-3::2017-∆avrBs1_PIP and pBBR1MCS-3::0468-∆avrBs1 constructs triggers HR in 8004∆avrBs1 but not in 8004∆hrcV, suggesting that the candidate protein encoded by XTG_RS02340 is a T3E, as its secretion signal was sufficient to restore avrBs1 activity while it is still T3SS-dependent (Figure S7). Following these results, this validated T3E was named XopBH.

Figure S7: Translocation assay results


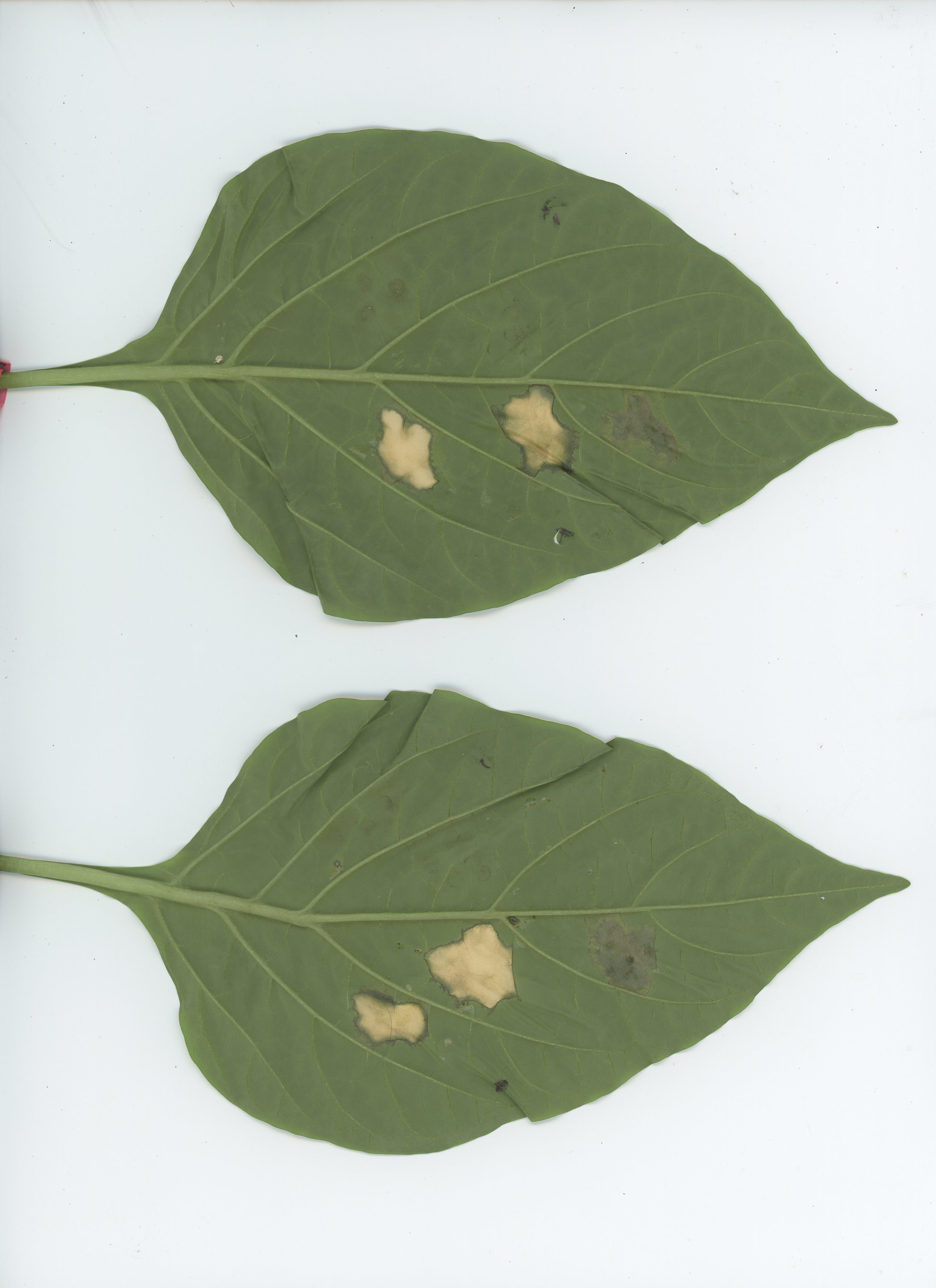


WT

Δ*avrBs1*

Δ*avrBs1*

XTG_RS02340

Leaf 1

Leaf 2

From Below


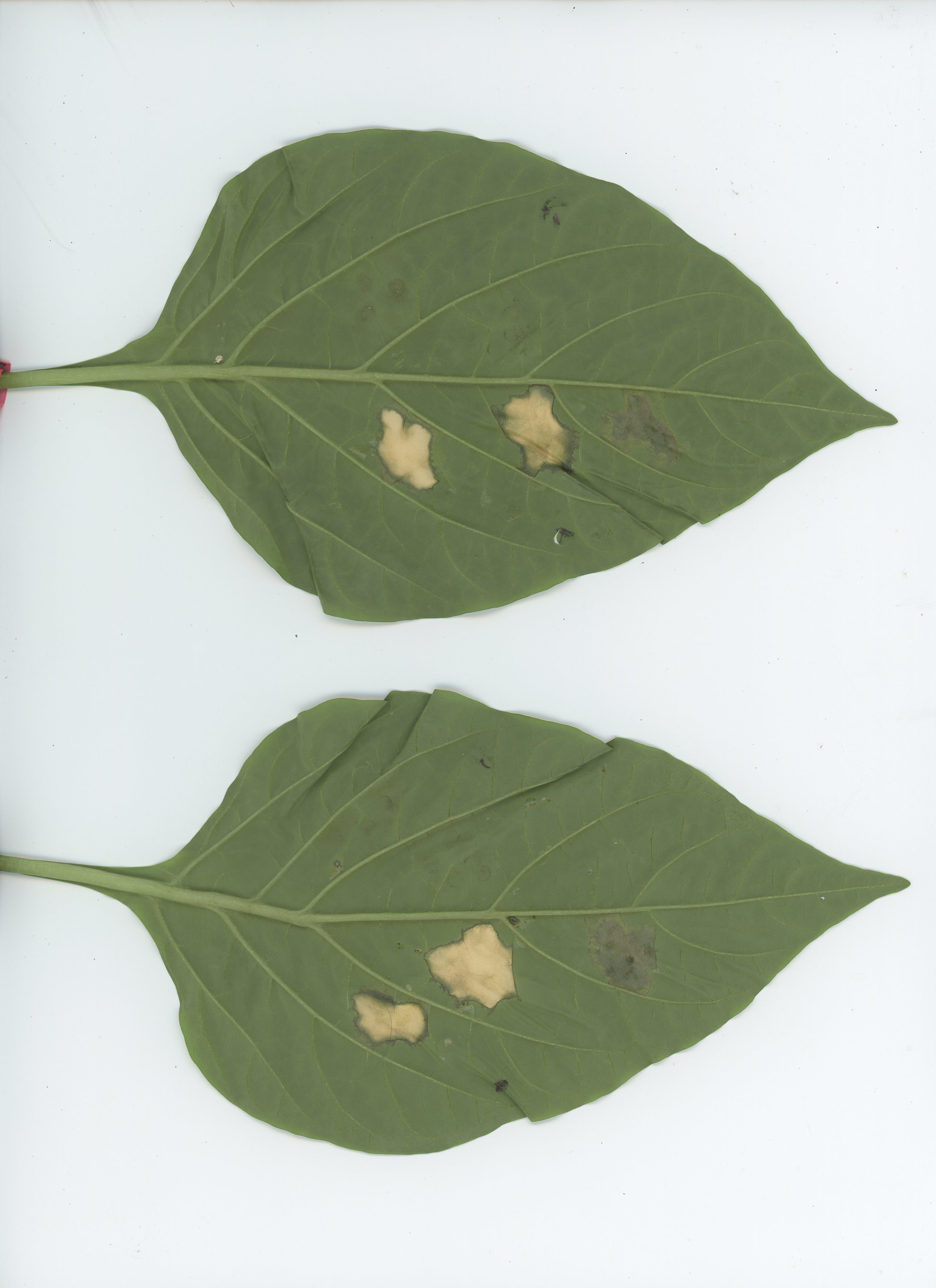


Δ*hrcV*

Δ*hrcV*

XTG_RS02340


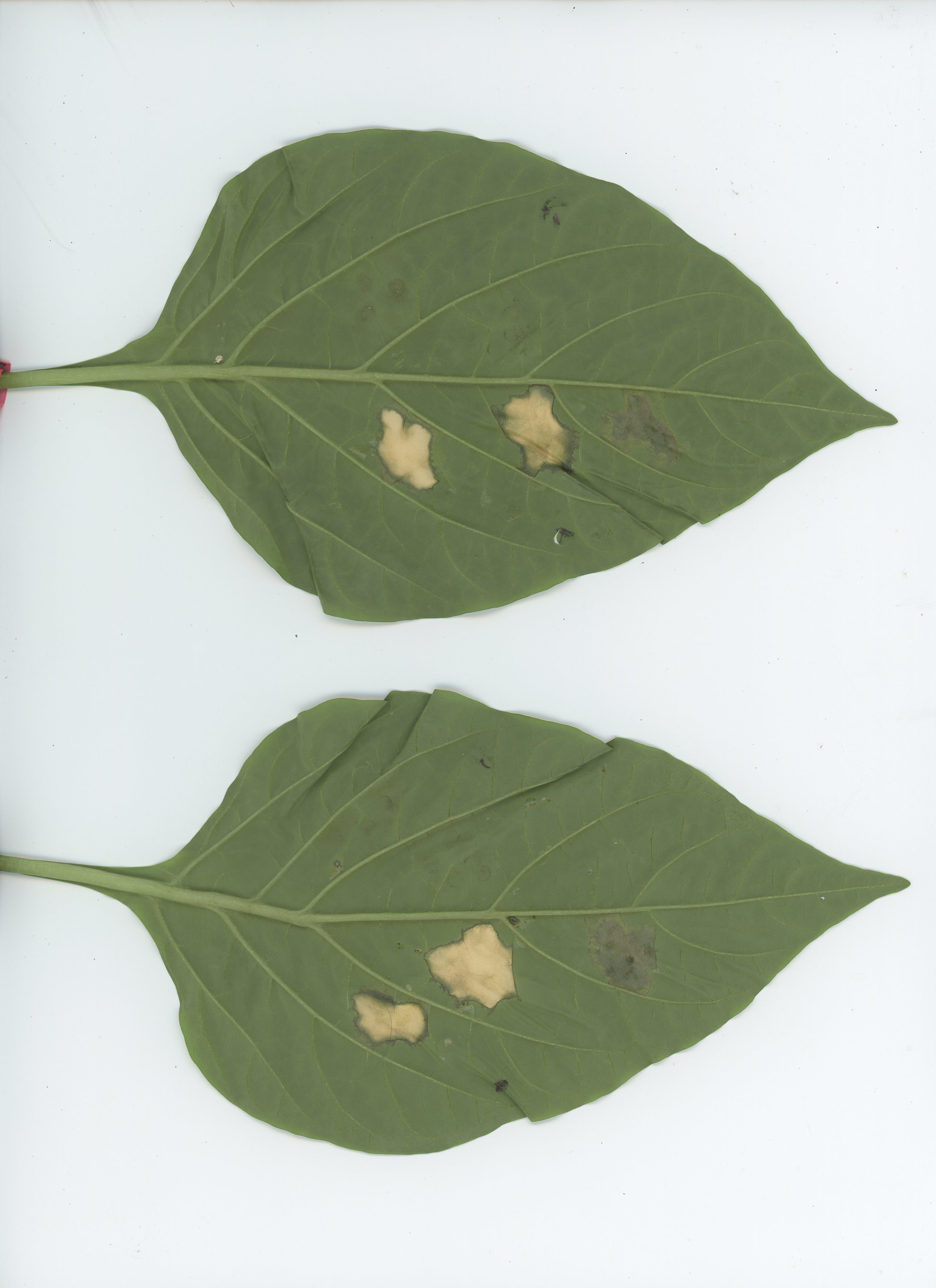

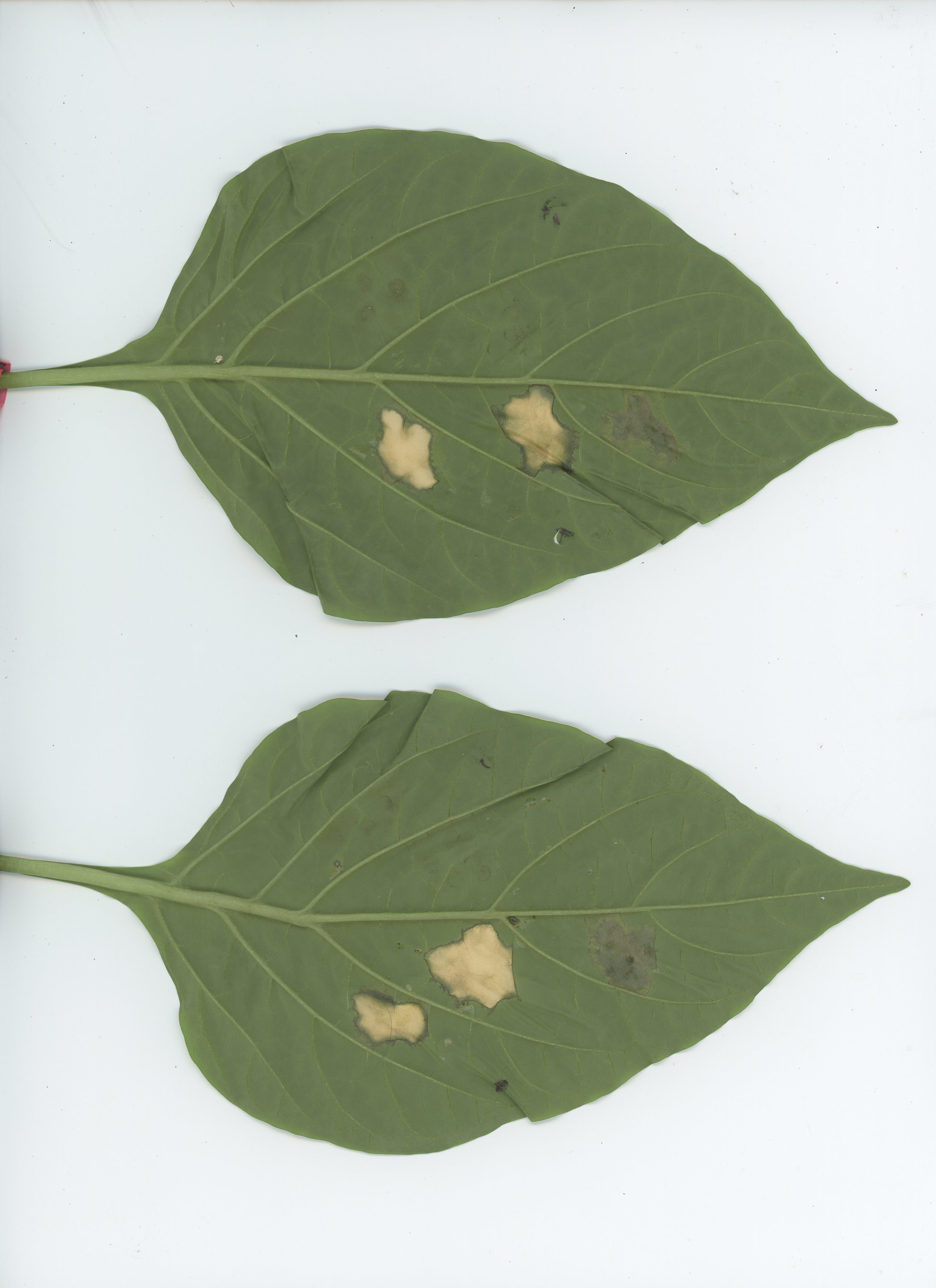

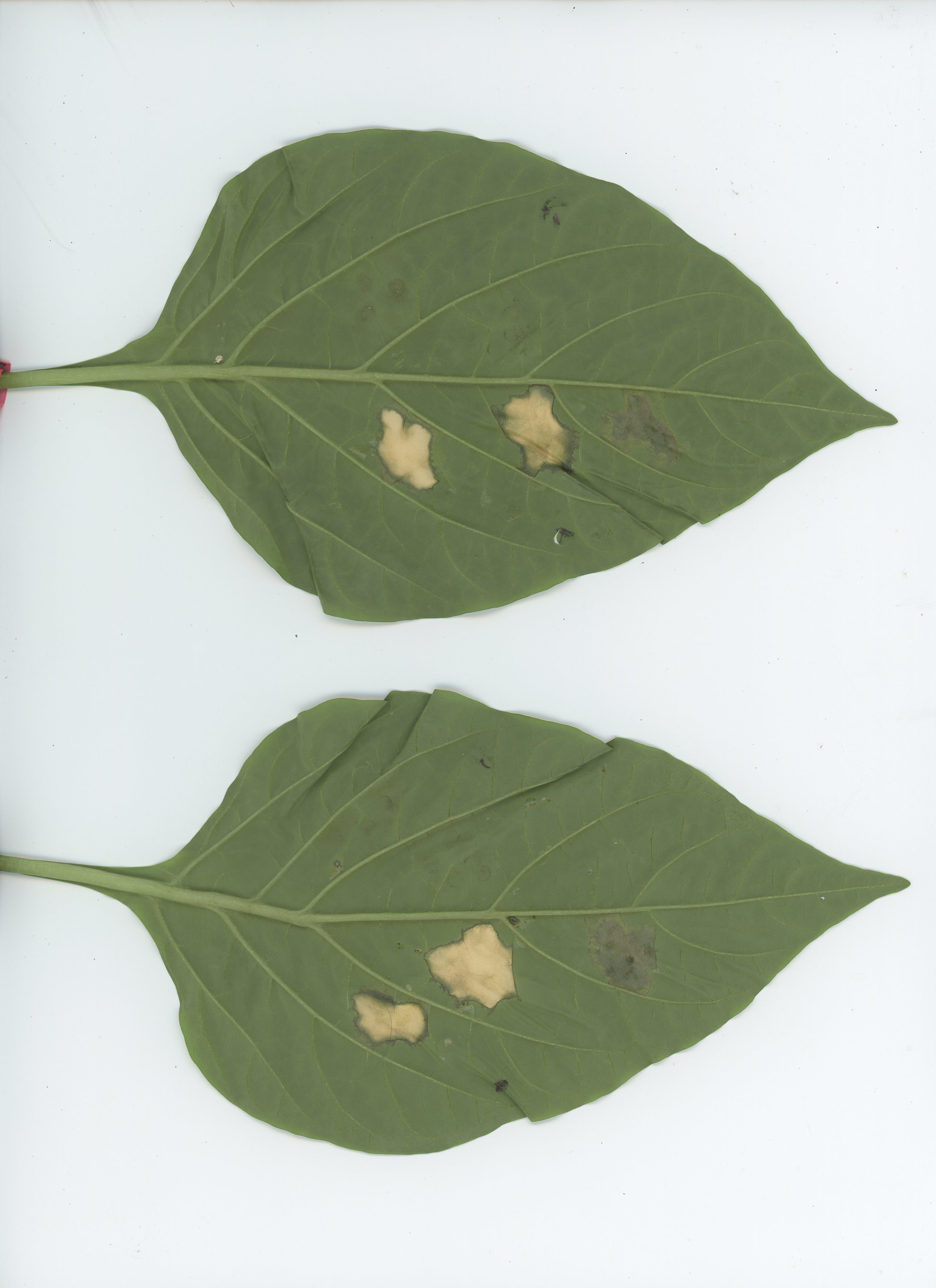

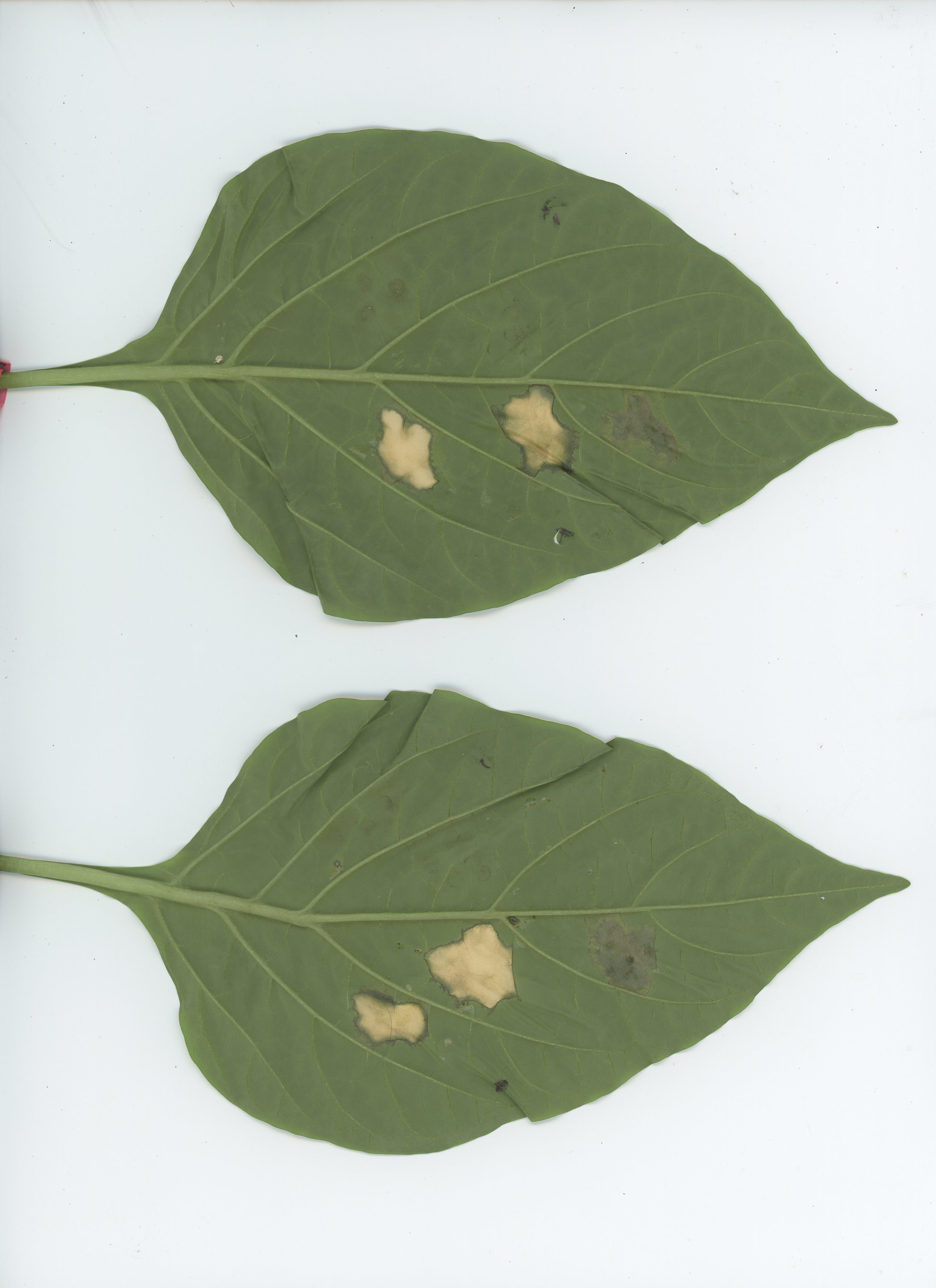

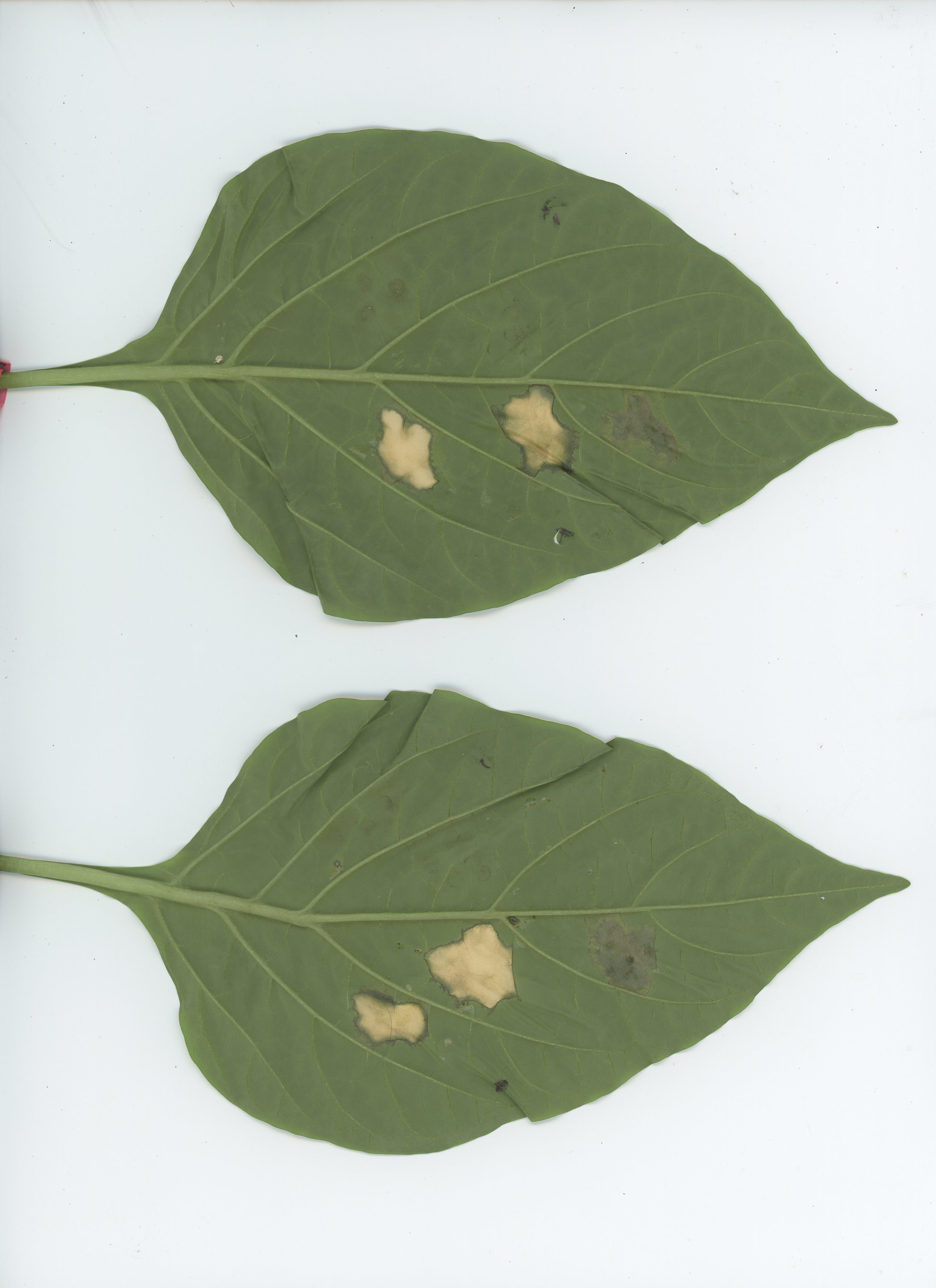

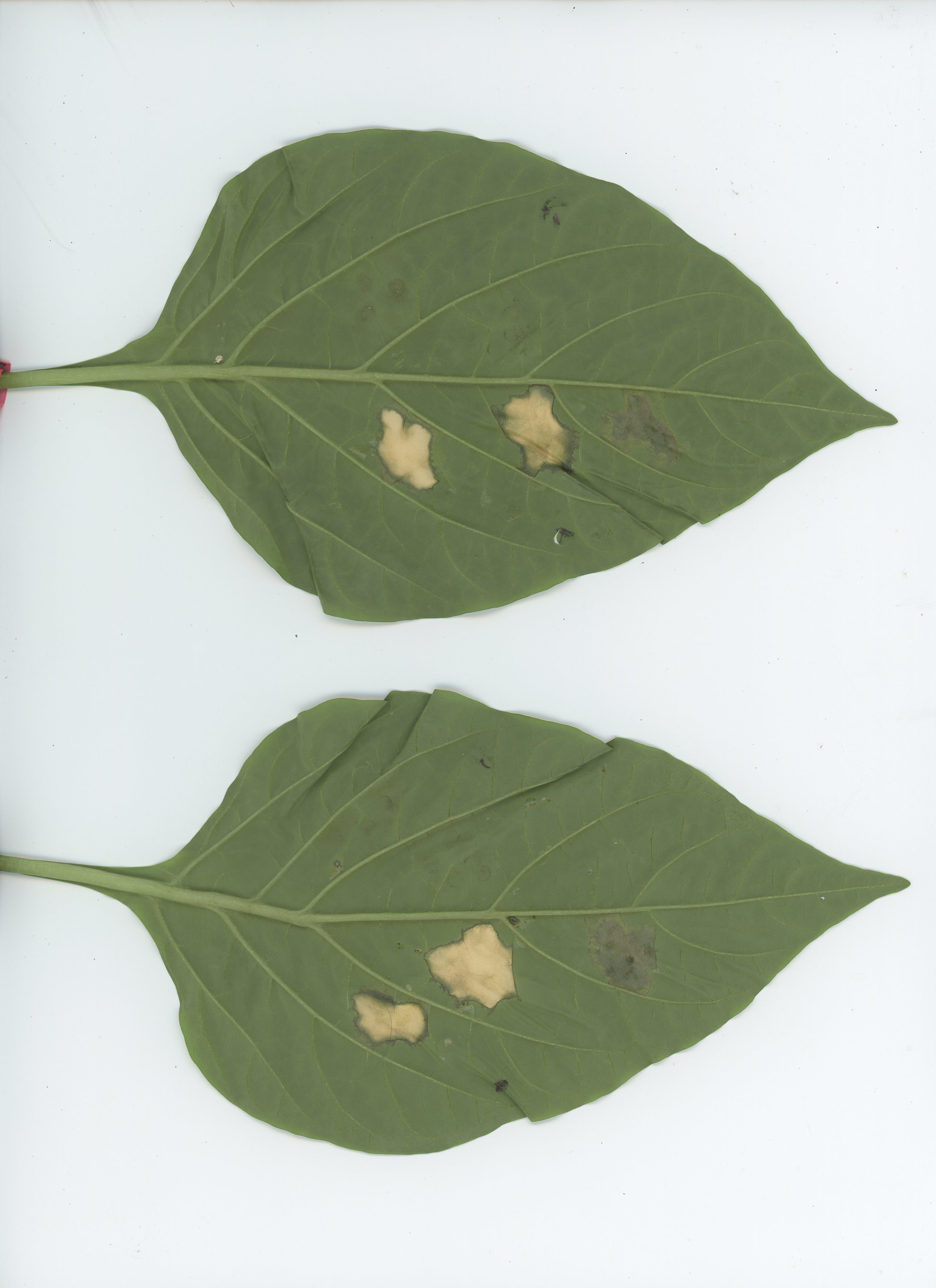

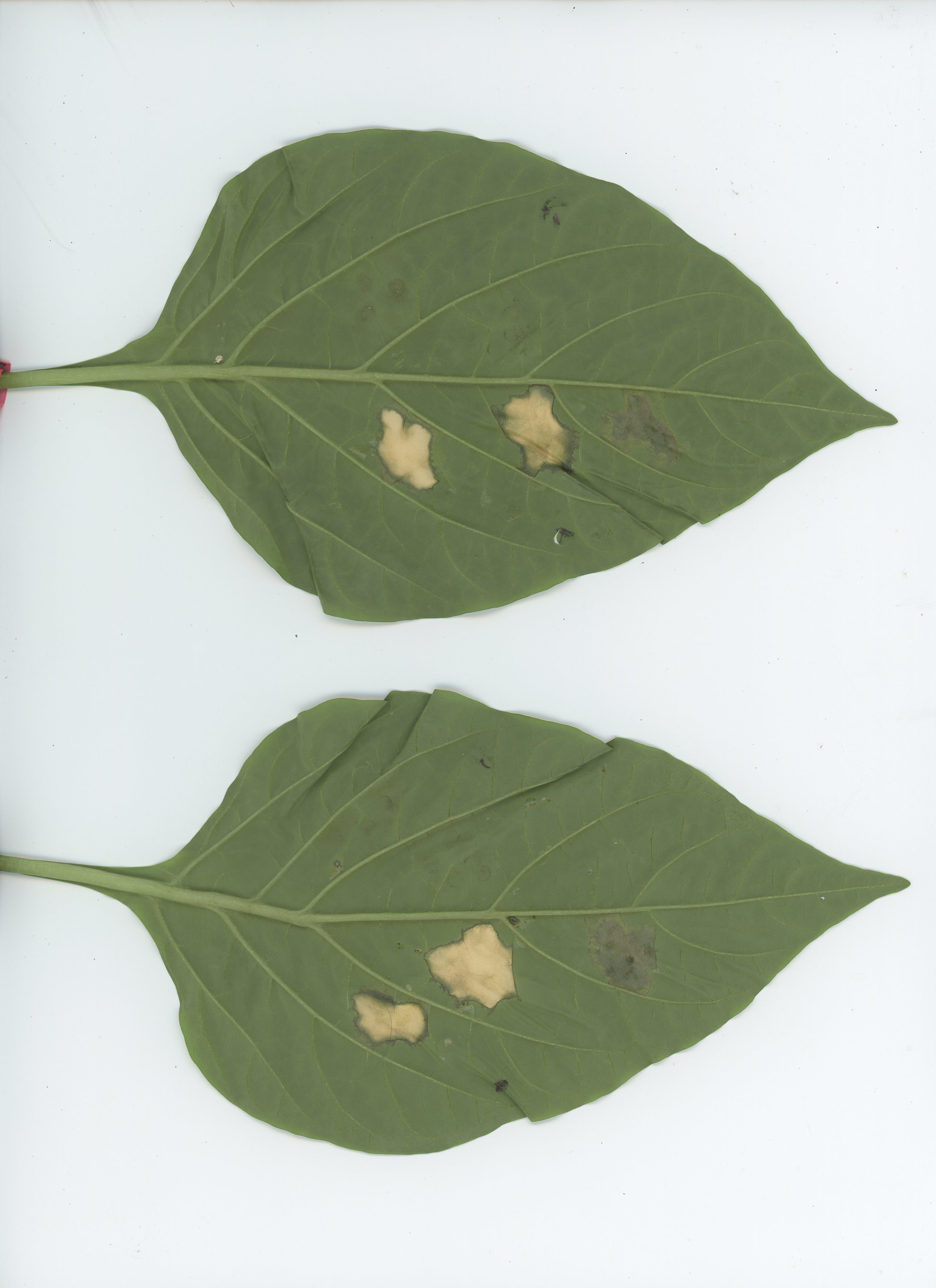

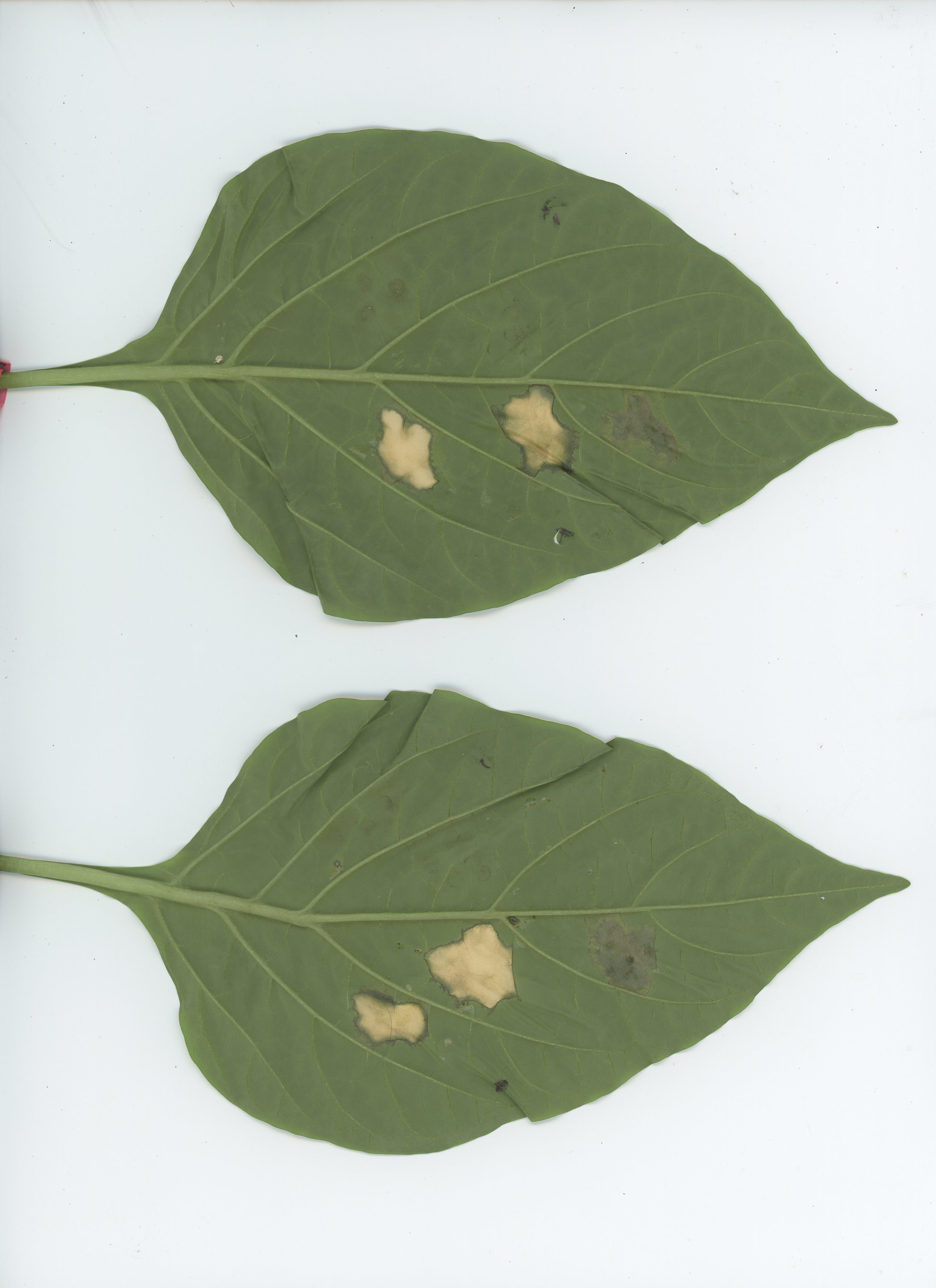

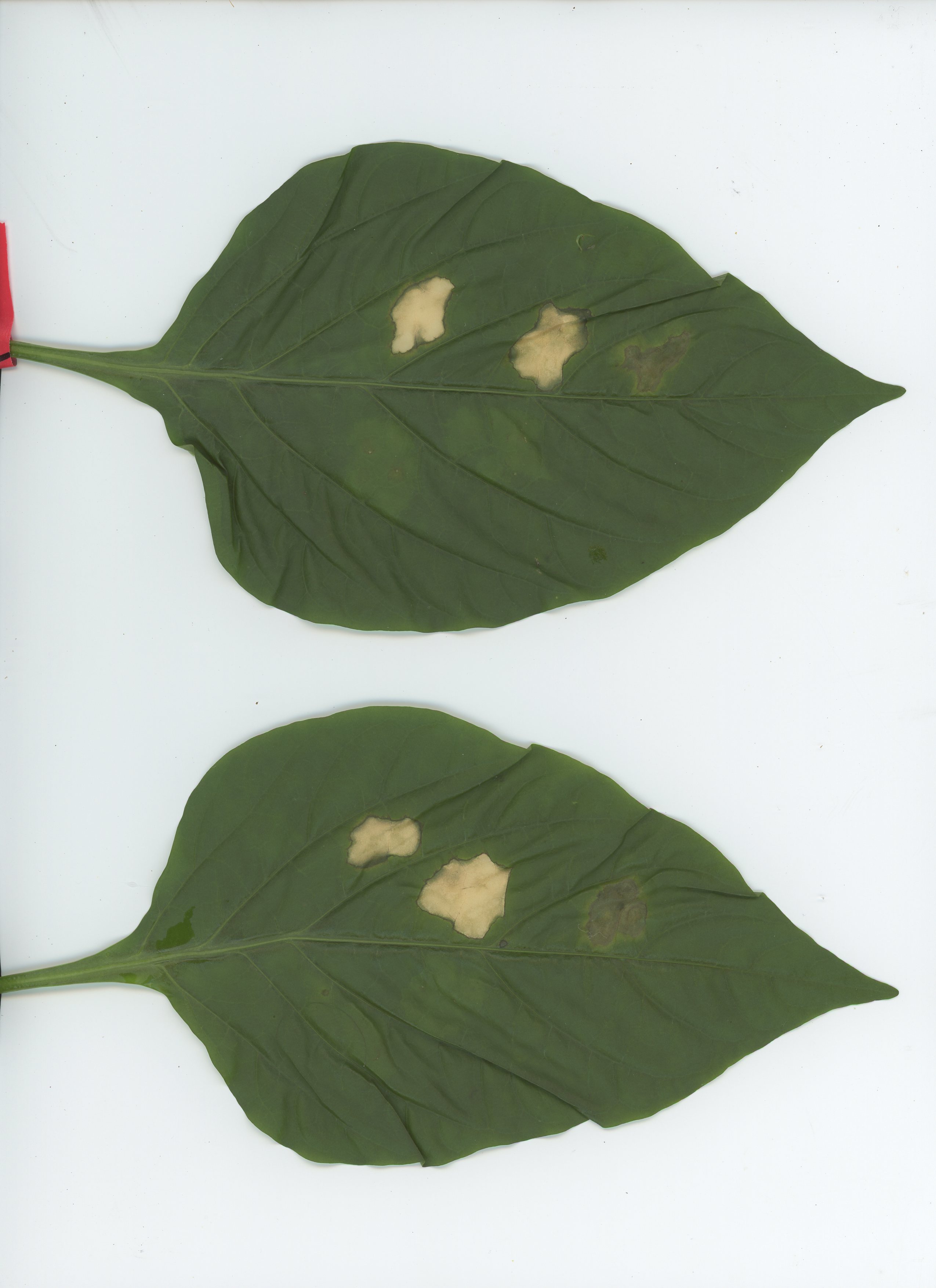

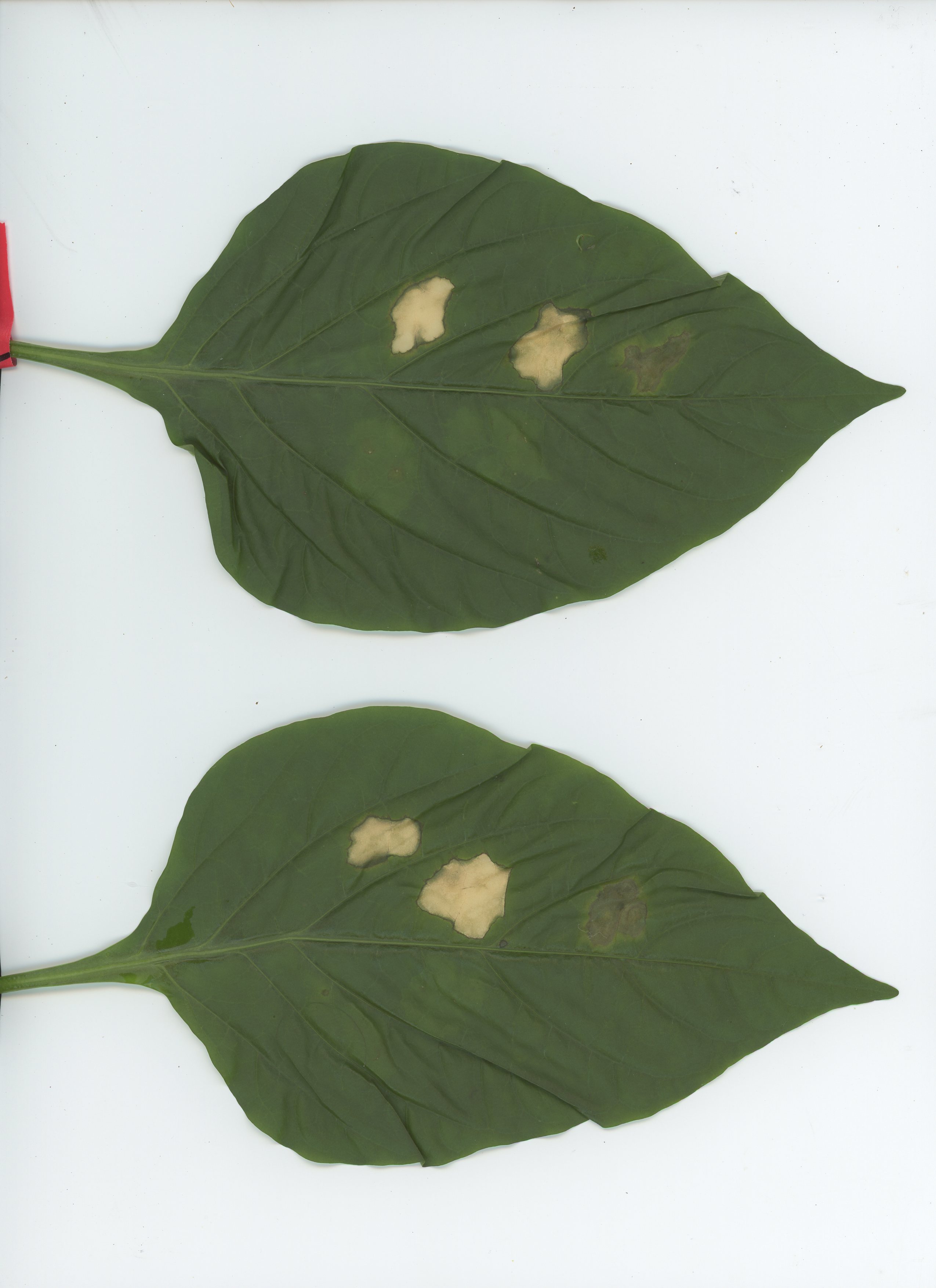

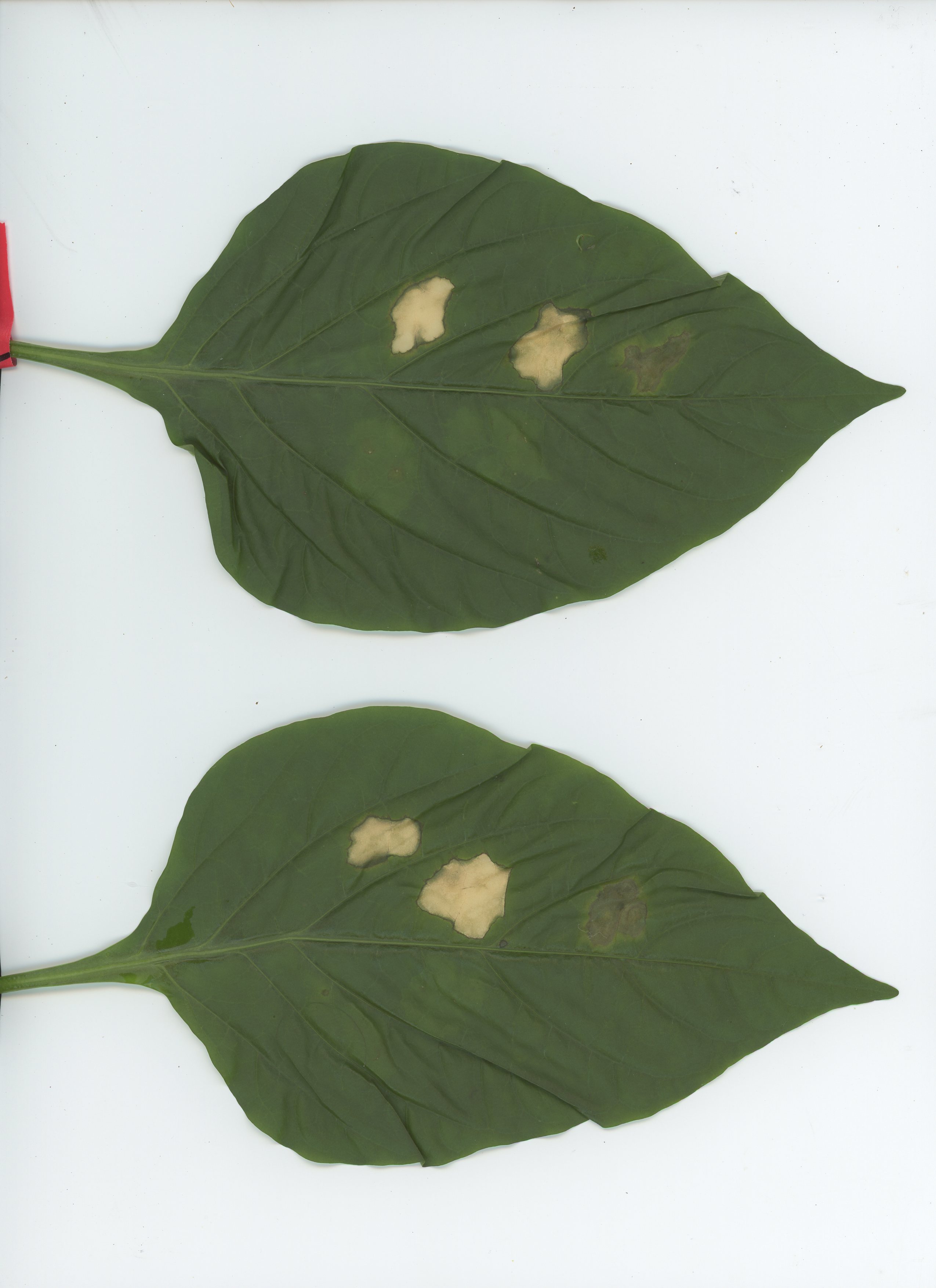

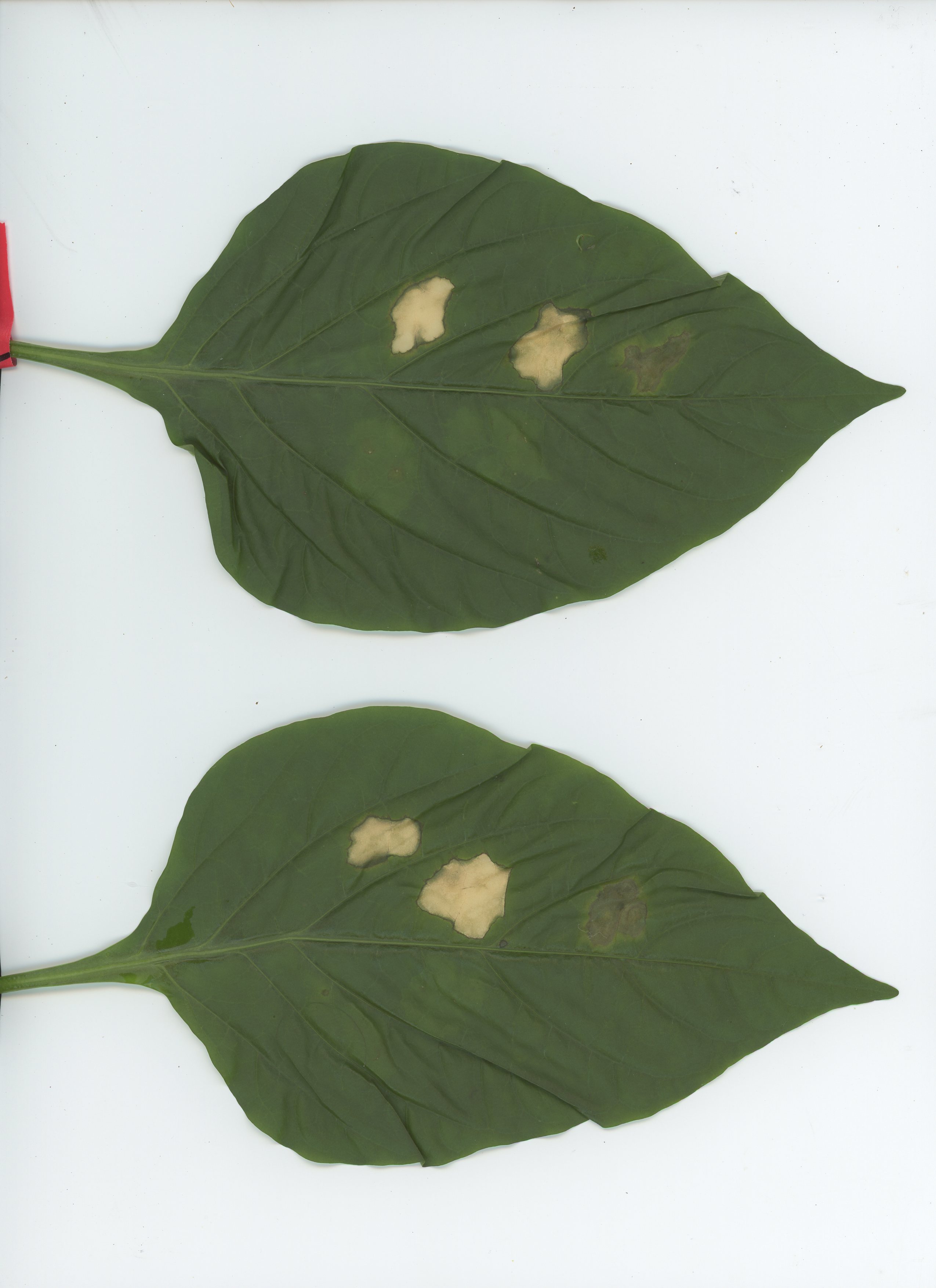

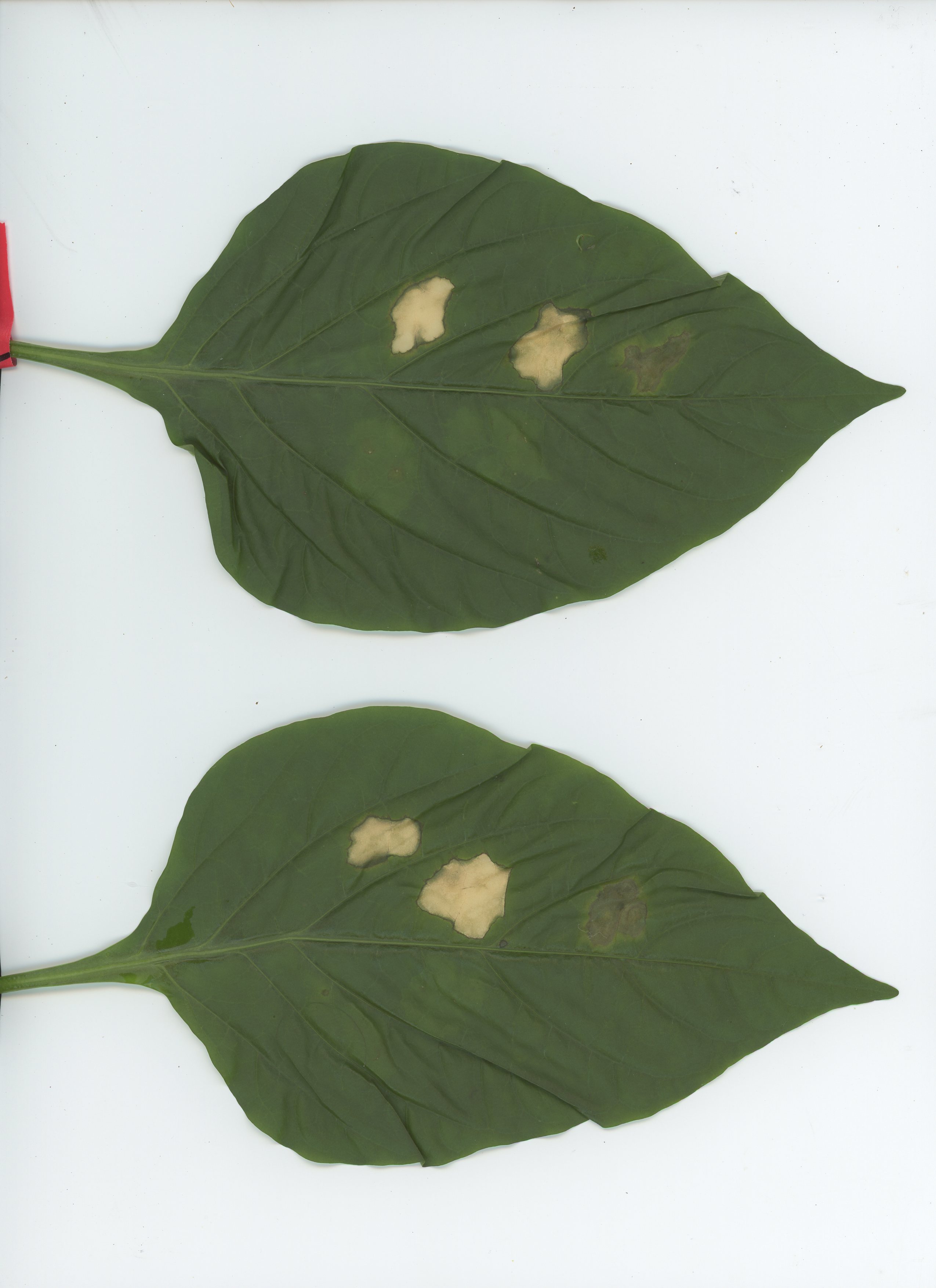

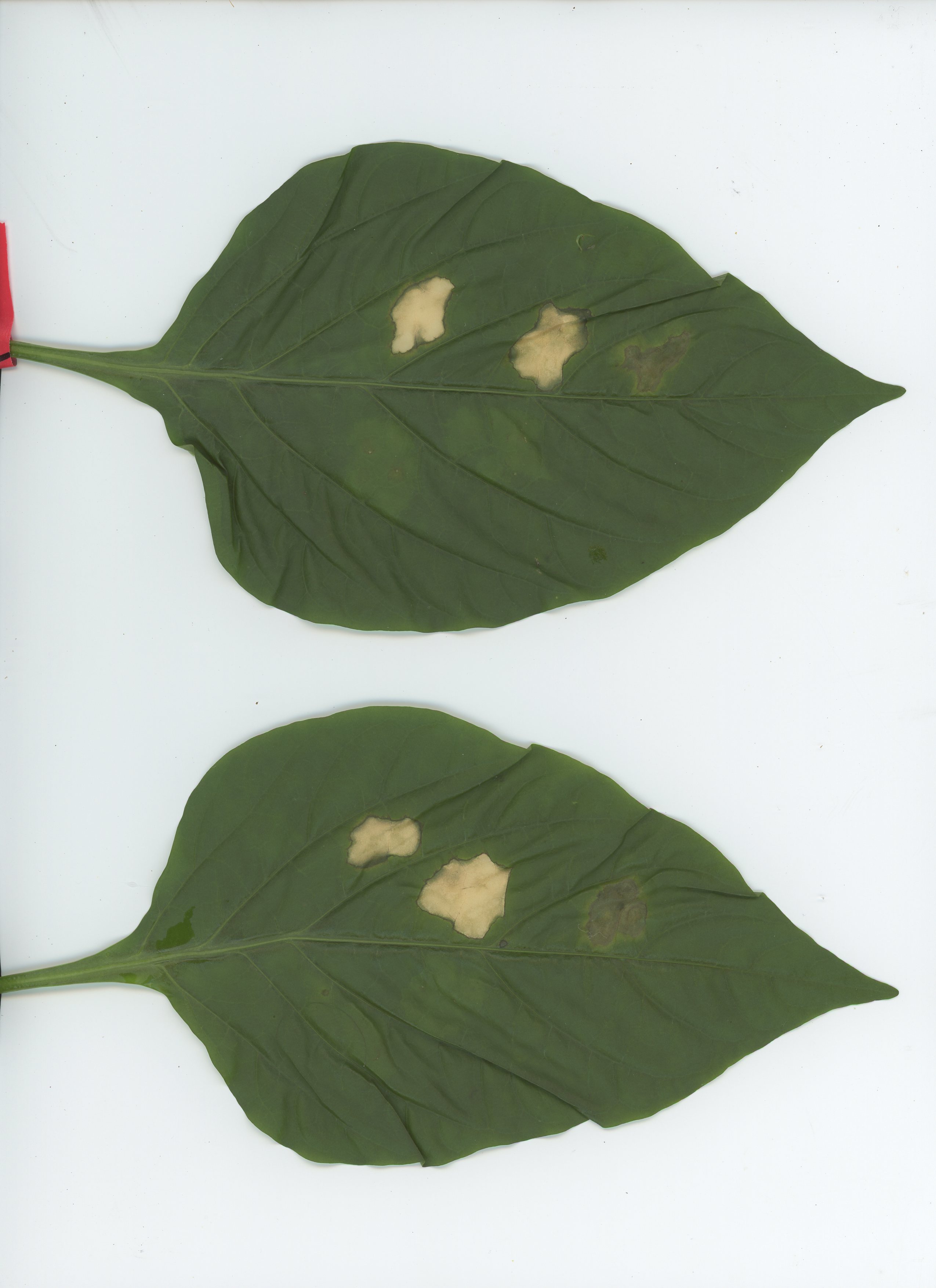

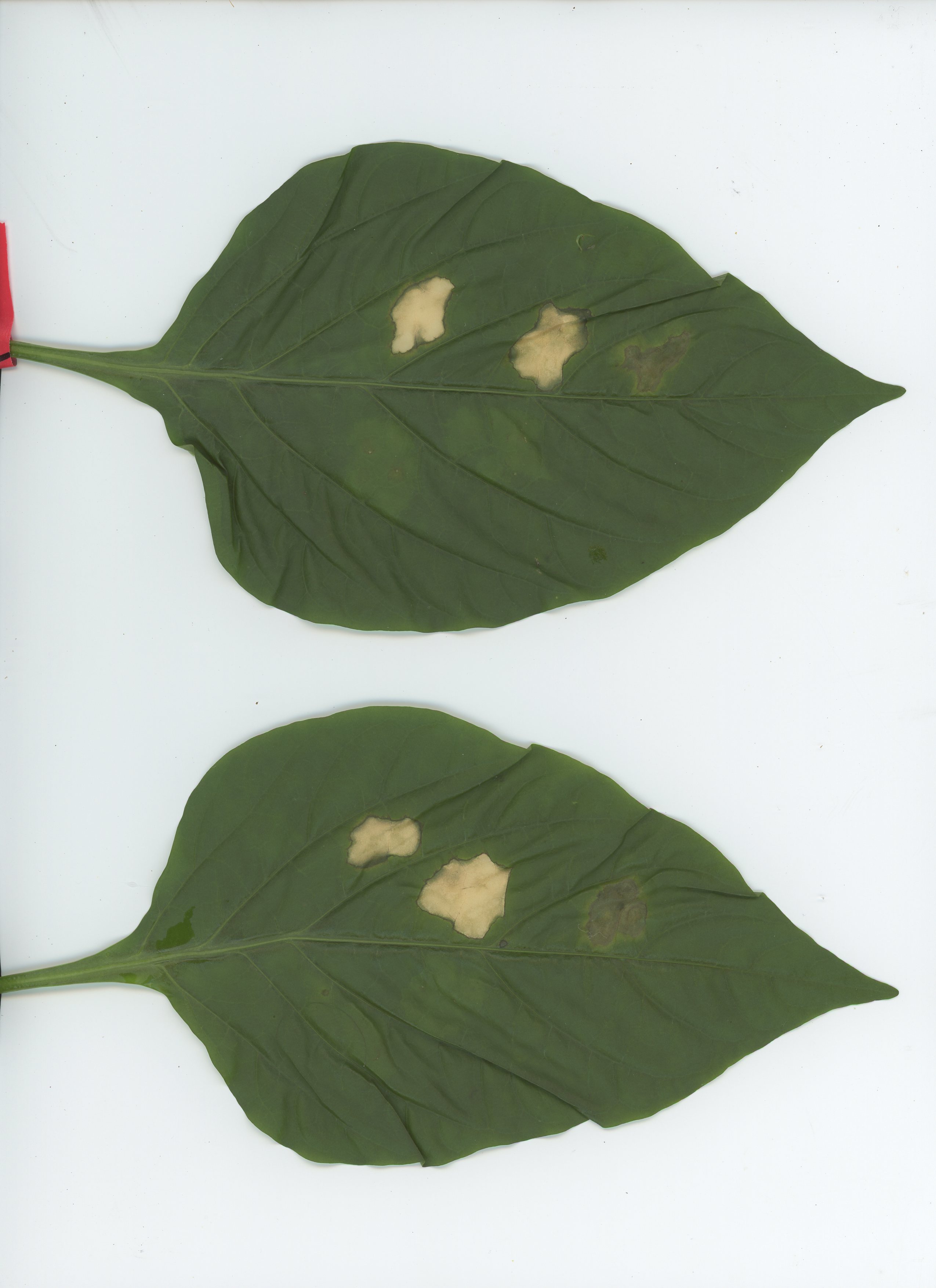

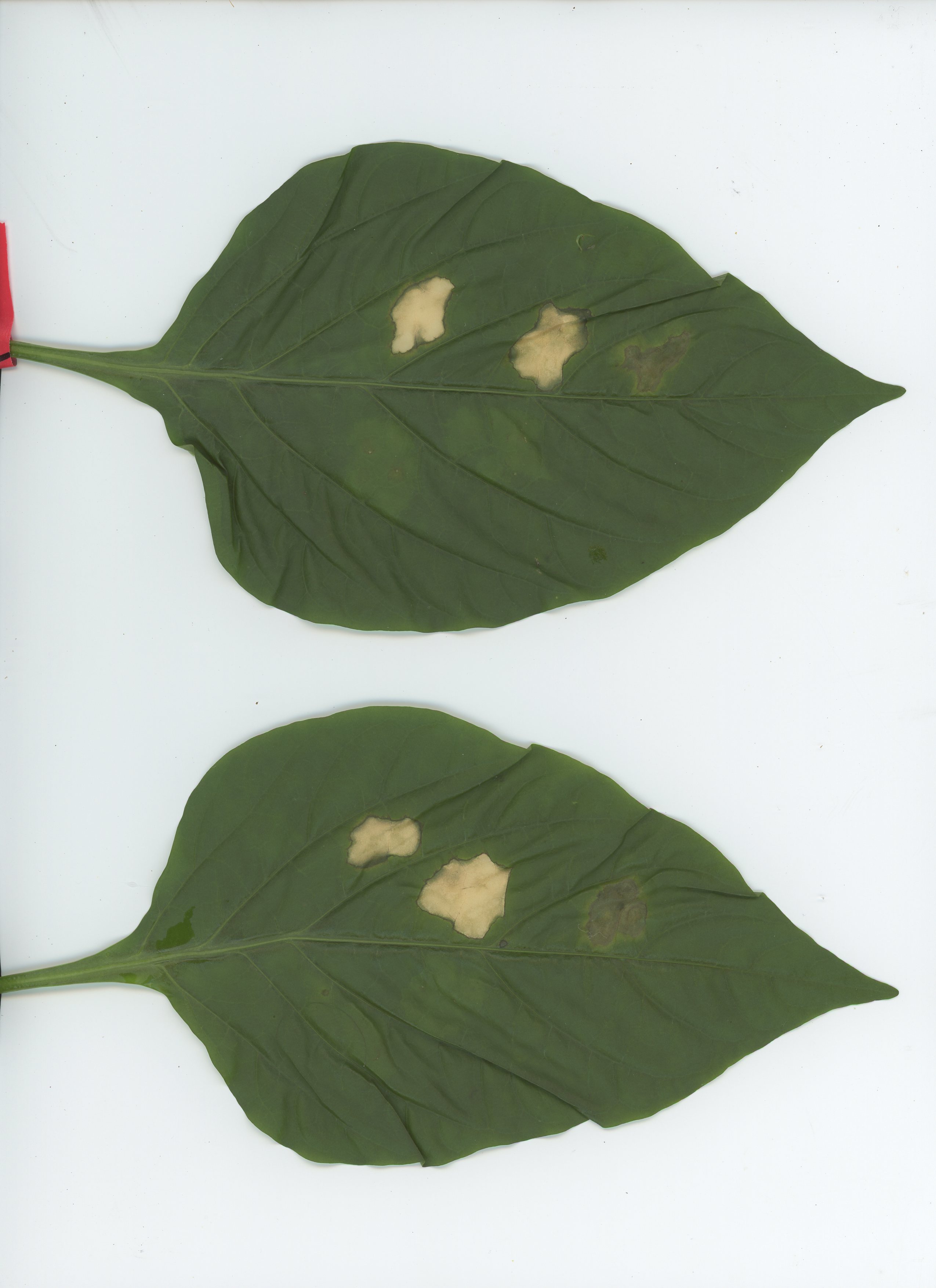

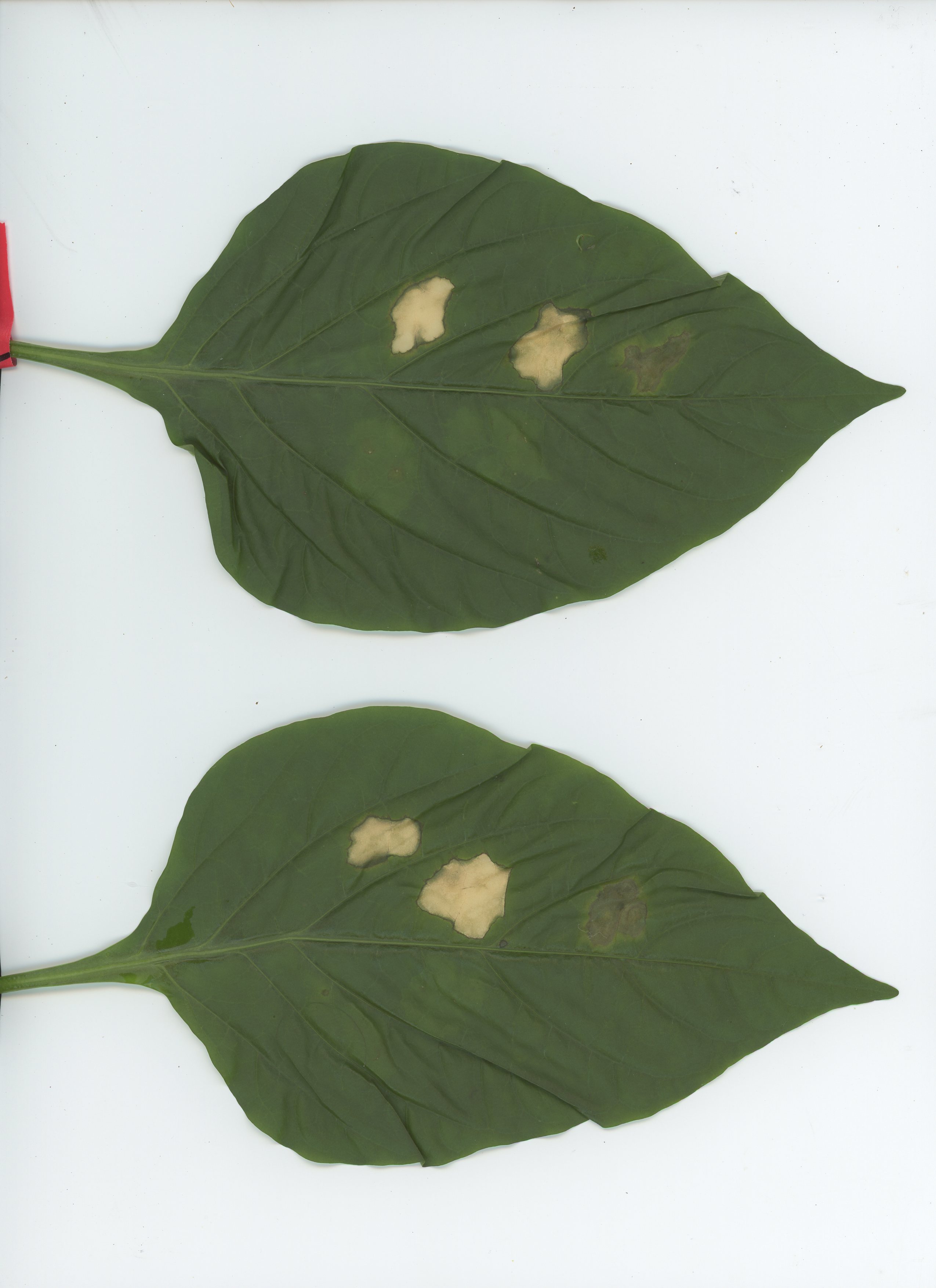

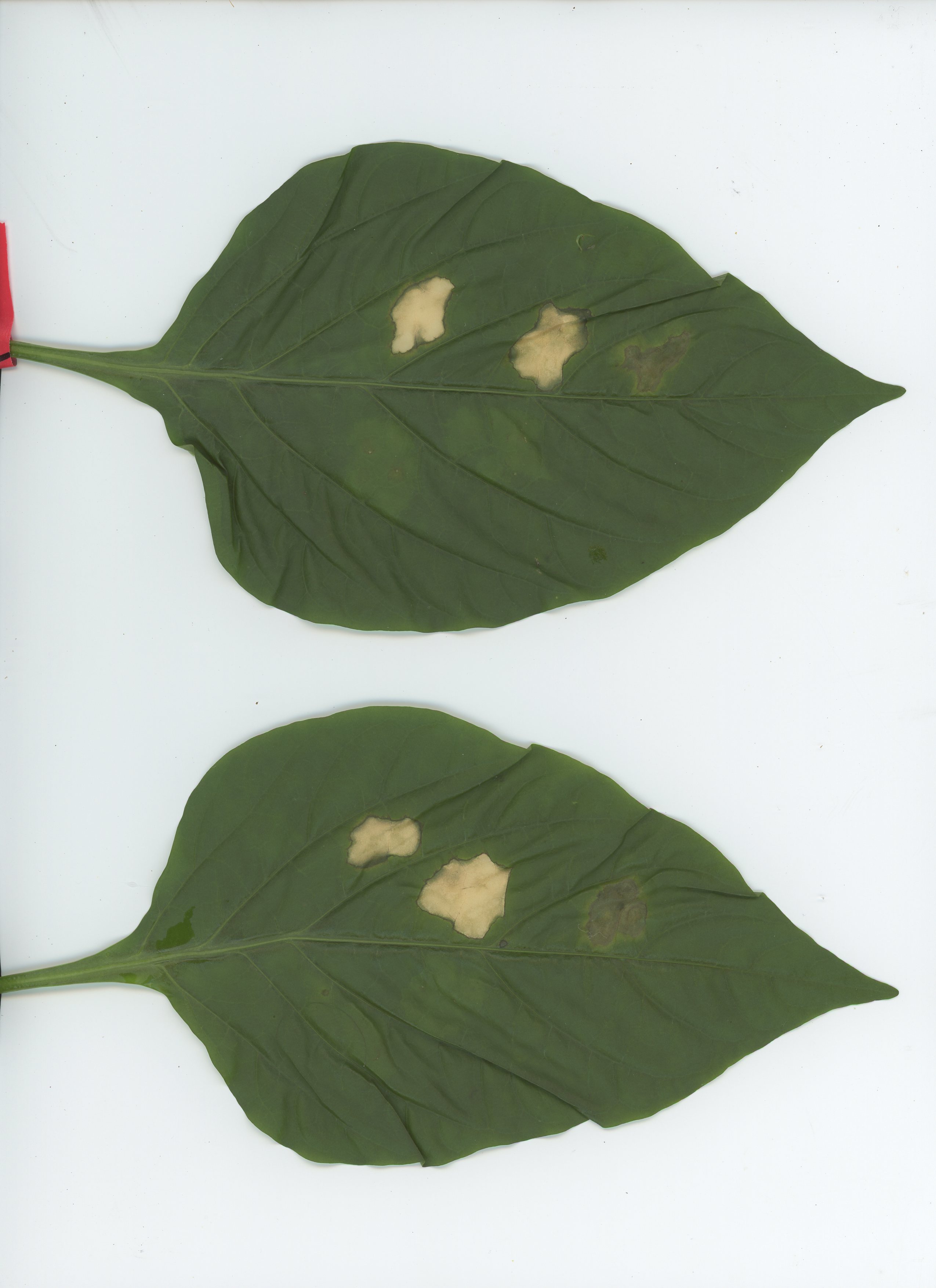


Leaf 1

Leaf 2

From Top

From Below

From Top

Running configuration

The data for all three example runs included the genome sequence and the coding sequences in FASTA format, and the annotation files in GFF format. We also included protein records of the following bacteria that do not encode T3SS: *Luteimonas* sp. MC1825 (accession NZ_CP061714.1), *Lysobacter capsici* 55 (accession NZ_CP011130.1), *Pseudoxanthomonas suwonensis* J1 (accession NZ_CP011144.1), *Stenotrophomonas maltophilia* K279a (accession NC_010943.1), and *Xylella fastidiosa* 9a5c (accessions NC_002488.3, NC_002489.3, and NC_002490.1). Additionally, we incorporated the prediction of the type III secretion signal using the improved pLM and searched for PIP-boxes in the promoters of the coding genes.

**Promoter analysis**

Multiple sequence alignment of XTG_RS02340 locus from CPBF 424 and its orthologs from various genomes. Highlighted in green and red are the annotated start and stop codons, respectively. In blue are the PIP-boxes, and in orange are the -10 motifs.

CFBP8132_MIGM01000012/PPT37905 ---------ACCATCTTAAGCAGTGCCAGACGGCGGCTAGTGCACTTCATACCAAGCAGC

LMG19146_OEQG01000033/SOU04629 ---------ACCATCTTAAGCAGTGCCAGACGGCGGCTAGTGCACTTCATACCAAGCAGC

NCPPB1630_JPHE01000005/KER88775 ---------ACCATCTTAAGCAGTGCCAGACGGCGGCTAGTGCACTTCATACCAAGCAGC

CFBP8147_MIGP01000013/PPT47098 ---------ACCATCTTAAGCAGTGCCAGACGGCGGCTAGTGCACTTCATACCAAGCAGC

3272_JAATIV010000002/NJC03069 ---------ACCATCTGAAGCAGTGCCAGACGGCGGCTAGTGCACTTCATACCAAGCAGC

CITA14_LXIB01000070/OBR72431 ---------ACCATCTGAAGCAGTGCCAGACGGCGGCTAGTGCACTTCATACCAAGCAGC

NCPPB1832_JPHC01000067/KER79886 ---------ACCATCTGAAGCAGTGCCAGACGGCGGCTAGTGGACTTCATACCAAGCAGC

1314c_HG992337/CAE6703782 ---------ACCATCTTAAGCAGTGCCAGACGGCGGCTAGTGGACTTCATACCAAGCAGC

WHRI7004_JBEGFG010000005/MEQ7893 ---------ACCATCTTAAGCAGTGCCAGACGGCGGCTAGTGGACTTCATACCAAGCAGC

WHRI5708_JBEGEU010000001/MEQ7970 ---------ACCATCTTAAGCAGTGCCAGACGGCGGCTAGTGGACTTCATACCAAGCAGC

CFBP8149_JACHNN010000001/MBB6572 ---------ACCATCTGAAGCAGTGCCAGACGGCGGCTAGTGGACTTCATACCAAGCAGC

CFBP8149_MIGQ01000003/PPT88030 ---------ACCATCTGAAGCAGTGCCAGACGGCGGCTAGTGGACTTCATACCAAGCAGC

1311a_HG992336/CAE6704102 ---------ACCATCTTAAGCAGTGCCAGACGGCGGCTAGTGGACTTCATACCAAGCAGC

3140_JAASRH010000001/NIK31525 ---------ACCATCTTAAGCAGTGCCAGACGGCGGCTAGTGGACTTCATACCAAGCAGC

WHRI7064_JBEGFI010000004/MEQ7937 ---------ACCATCTTAAGCAGTGCCAGACGGCGGCTAGTGGACTTCATACCAAGCAGC

CFBP8140_JACIIT010000002/MBB6257 ---------ACCATCTTAAGCAGTGCCAGACGGCGGCTAGTGGACTTCATACCAAGCAGC

CFBP1022_MDRU01000008/PPU10530 ----------CCATCTGAAGCAGTACCAGACGGCGGCTAGTGGACTTCATACCAAGCAGC

CFBP7697_MDDZ01000001/PPU47721 ----------CCATCTGAAGCAGTACCAGACGGCGGCTAGTGGACTTCATACCAAGCAGC

CFBP8142_MIGO01000012/PPT66874 AGCAAGCGGACCATCTGAAGCAGTGCCAGACGGCGGCTAGTGCACTTCATACCAAGCAGC

CFBP6762_MDRW01000018/PPU23895 ---------ACCATCTGAAGCAGTGCCAGACGGCGGCTAATGCACTTCATACCAAGCAGC

CFBP6762_OEQE01000006/SOT93765 ---------ACCATCTGAAGCAGTGCCAGACGGCGGCTAATGCACTTCATACCAAGCAGC

CPBF1586_LR962897/CAD7376296 ---------ACCATCTGAAGCAGTGCCAGACGGCGGCCAATGCACTTCATACCAAGCAGC

NBC5720_JAWIII010000009/MDV24505 --------AATACTCTT--GCCGCGCGCGAAGCTG-----------TCCTGGTGAAAAAT

NBC5721_JBEOLS010000004/MER30529 --------AATACTCTT--GCCGCGCGCGAAGCTG-----------TCCTGGTGAAAAAT

NCPPB2970_JAJFNJ020000003/MEC388 ---TGCGAAAGACTCTT--GCCGTGCGCGAAGATG-----------TCCTGCCGAGAAAC

NCPPB4692_CP166090/XDZ21896 ------GAAAGACTCTT--GCCGCGCGCGAAGATG-----------TCCTGCTGAAAAGT

CFBP7622_JAASRB010000002/NIJ9363 --------AAGACTCTT--GCCGCGCGCGAAGATG-----------TCCTGCTGAAAAAT

CPBF766_HG999364/CAG2083774 --------AAGACTCTT--TCCGCGCGCGAAGATG-----------TCCTGCTGAAAAAT

CPBF424_LR994544/CAE1133144 ---------AGACTCTT--GCCGCGCGCGAAGATG-----------TCCTGCTGAAAAAT

CPBF424_UIHB01000001/SUZ26707 ---------AGACTCTT--GCCGCGCGCGAAGATG-----------TCCTGCTGAAAAAT

*** * * * ** * * ** * * *

CFBP8132_MIGM01000012/PPT37905 -TACGAACGCGACACAGGTC---------GTTCTGCCGAGCA-TCCAGGACCAGGTTGTG

LMG19146_OEQG01000033/SOU04629 -TACGAACGCGACACAGGTC---------GTTCTGCCGAGCA-TCCAGGACCAGGTTGTG

NCPPB1630_JPHE01000005/KER88775 -TACGAACGCGACACAGGTC---------GTTCTGCCGAGCA-TCCAGGACCAGGTTGTG

CFBP8147_MIGP01000013/PPT47098 -TACGAACGCGACACAGGTC---------GTTCTGCCGAGCA-TCCAGGACCAGGTTGTG

3272_JAATIV010000002/NJC03069 -TACGAACGCGACACAGGTC---------GTTTTGCCGAGCA-TCCAGGAACAGGTTGCG

CITA14_LXIB01000070/OBR72431 -TACGAACGCGACACAGGTC---------GTTTTGCCGAGCA-TCCAGGAACAGGTTGCG

NCPPB1832_JPHC01000067/KER79886 -TACGAACGCGACACAGGTC---------GTTTTTCCGAGCA-TCCAGGACCAGGTTGCG

1314c_HG992337/CAE6703782 -TACGAACGCGACACAGGTC---------GTTTTTCCGAGCA-TCCAGGACCAGGTTGCG

WHRI7004_JBEGFG010000005/MEQ7893 -TACGAACGCGACACAGGTC---------GTTTTTCCGAGCA-TCCAGGACCAGGTTGCG

WHRI5708_JBEGEU010000001/MEQ7970 -TACGAACGCGACACAGGTC---------GTTCTGCCGAGCA-TCCAGGACCAGGTTGCG

CFBP8149_JACHNN010000001/MBB6572 -TACGAACGCGACACAGGTC---------GTTTTTCCGAGCA-TCCAGGACCAGGTTGCG

CFBP8149_MIGQ01000003/PPT88030 -TACGAACGCGACACAGGTC---------GTTTTTCCGAGCA-TCCAGGACCAGGTTGCG

1311a_HG992336/CAE6704102 -TACGAACGCGACACAGGTC---------GTTTTTCCGAGCA-TCCAGGACCAGGTTGCG

3140_JAASRH010000001/NIK31525 -TACGAACGCGACACAGGTC---------GTTTTTCCGAGCA-TCCAGGACCAGGTTGCG

WHRI7064_JBEGFI010000004/MEQ7937 -TACGAACGCGACACAGGTC---------GTTTTTCCGAGCA-TCCAGGACCAGGTTGCG

CFBP8140_JACIIT010000002/MBB6257 -TACGAACGCGACACAGGTC---------GTTTTTCCGAGCA-TCCAGGACCAGGTTGCG

CFBP1022_MDRU01000008/PPU10530 -TACGAACGCGACACAGGTC---------GTTTTGCCGAGCA-TCCAGAACCAGGTTGCG

CFBP7697_MDDZ01000001/PPU47721 -TACGAACGCGACACAGGTC---------GTTTTGCCGAGCA-TCCAGAACCAGGTTGCG

CFBP8142_MIGO01000012/PPT66874 -TACGAACGGGACACAGGTC---------GTTTTGCCGAGCA-TCCAGGACCAGGTTGCG

CFBP6762_MDRW01000018/PPU23895 -TACGAACGCGACACAGGTC---------GTTTGGCCGAGCA-TCCAGGACCAGGTTGCG

CFBP6762_OEQE01000006/SOT93765 -TACGAACGCGACACAGGTC---------GTTTGGCCGAGCA-TCCAGGACCAGGTTGCG

CPBF1586_LR962897/CAD7376296 -TACGAACGCGACACAGGTG---------GTTTGGCCGAGCA-TCCAGGACCAGGTTGCG

NBC5720_JAWIII010000009/MDV24505 GTGCAATCGAGACCTGCATCAAGTGCACCACGCCCCGGAGCAATGCAAGATCAAGTTGCA

NBC5721_JBEOLS010000004/MER30529 GTGCAATCGAGACCTGCATCAAGTGCACCACGCCCCGGAGCAATGCAAGATCAAGTTGCA

NCPPB2970_JAJFNJ020000003/MEC388 GTGCAACCGAGACCTGCATCAAGTGCACCGCACTCCGAAGCAATGCGAGATCACGTTGCA

NCPPB4692_CP166090/XDZ21896 GTGCAACCGAGACCTGGATCATGTGCGCCATAGTGCGGAGCACTACAAAATCA-GTTGCA

CFBP7622_JAASRB010000002/NIJ9363 GTGCAACCGAGACCTGGATCATGTGCCTTGCACTCCGGAGCACGACAGGATCATGTTGCA

CPBF766_HG999364/CAG2083774 GTGCAACCGAGACCTGGATCATGTGCCTTGCACTCCGGAACACGACAGGATCATGTTGCA

CPBF424_LR994544/CAE1133144 GTGCAACCGAGACCTGGATCATGTGCCTTGCACTCTGGAGCACGACAGGATCATGTTGCA

CPBF424_UIHB01000001/SUZ26707 GTGCAACCGAGACCTGGATCATGTGCCTTGCACTCTGGAGCACGACAGGATCATGTTGCA

* * * ** *** * * ** * * ** ****

CFBP8132_MIGM01000012/PPT37905 CAAAACCGATCTGAGATTAACGCGCTTACA-CACCCGGATATTCGATTCGTATCGATATG

LMG19146_OEQG01000033/SOU04629 CAAAACCGATCTGAGATTAACGCGCTTACA-CACCCGGATATTCGATTCGTATCGATATG

NCPPB1630_JPHE01000005/KER88775 CAAAACCGATCTGAGATTAACGCGCTTACA-CACCCGGATATTCGATTCGTATCGATATG

CFBP8147_MIGP01000013/PPT47098 CAAAACCGATCTGAGATTAACGCGCTTACA-CACCCGGATATTCGATTCGTATCGATATG

3272_JAATIV010000002/NJC03069 CAAAACCGATCTGAGATTAACGCGCTTACG-CCTCCGGATATTCGATTCGTATCGATATG

CITA14_LXIB01000070/OBR72431 CAAAACCGATCTGAGATTAACGCGCTTACG-CCTCCGGATATTCGATTCGTATCGATATG

NCPPB1832_JPHC01000067/KER79886 CAAAACCGATCTGGGATTAACGCGCTTGCG-CCCCCGGATATTCGATTCGTATCGATATG

1314c_HG992337/CAE6703782 CAAAACCGATCTGGGATTAACGCGCTTGCG-CCCCCGGATATTCGATTCGTATCGATATG

WHRI7004_JBEGFG010000005/MEQ7893 CAAAACCGATCTGGGATTAACGCGCTTGCG-CCCCCGGATATTCGATTCGTATCGATATG

WHRI5708_JBEGEU010000001/MEQ7970 CAAAACCGATCTGGGATTAACGCGCTTGCG-CCCCCGGATATTCGATTCGTATCGATATG

CFBP8149_JACHNN010000001/MBB6572 CAAAACCGATCTGGGATTAACGCGCTTGCG-CCCCCGGATATTCGATTCGTATCGATATG

CFBP8149_MIGQ01000003/PPT88030 CAAAACCGATCTGGGATTAACGCGCTTGCG-CCCCCGGATATTCGATTCGTATCGATATG

1311a_HG992336/CAE6704102 CAAAACCGATCTGGGATTAACGCGCTTGCG-CCCCCGGATATTCGATTCGTATCGATATG

3140_JAASRH010000001/NIK31525 CAAAACCGATCTGGGATTAACGCGCTTGCG-CCCCCGGATATTCGATTCGTATCGATATG

WHRI7064_JBEGFI010000004/MEQ7937 CAAAACCGATCTGGGATTAACGCGCTTGCG-CCCCCGGATATTCGATTCGTATCGATATG

CFBP8140_JACIIT010000002/MBB6257 CAAAACCGATCTGGGATTAACGCGCTTGCG-CCCCCGGATATTCGATTCGTATCGATATG

CFBP1022_MDRU01000008/PPU10530 CAAAACCGATCTGGGATTAACGGGCTTGTGCCCCCCGGATATTCGATTCGTATCGATATG

CFBP7697_MDDZ01000001/PPU47721 CAAAACCGATCTGGGATTAACGGGCTTGTGCCCCCCGGATATTCGATTCGTATCGATATG

CFBP8142_MIGO01000012/PPT66874 CAAAACCGATCTGAGA---------TTGCG-CCCCCGGATATTCAATTCGTATCGATATG

CFBP6762_MDRW01000018/PPU23895 CAAAACCGATCGGGGATTAACGCGCTTGCG-CCCCCGGATATTCGATTCGTATCGATATG

CFBP6762_OEQE01000006/SOT93765 CAAAACCGATCGGGGATTAACGCGCTTGCG-CCCCCGGATATTCGATTCGTATCGATATG

CPBF1586_LR962897/CAD7376296 CAAAACCGATCGGGGATTAACGCGCTTGCG-CCCCCGGATATTCGATTCGTATCGATATG

NBC5720_JAWIII010000009/MDV24505 CAAAATGGATCTGAGATTATCGCGCTTACA--CTCCGGACATTCGATTCGCATCGATATG

NBC5721_JBEOLS010000004/MER30529 CAAAATGGATCTGAGATTATCGCGCTTACA--CTCCGGACATTCGATTCGCATCGATATG

NCPPB2970_JAJFNJ020000003/MEC388 TACCACGGGTCGGGGATTAAGTCGCTTACA--CCCCGGAC-----ATTCGCATCGATATG

NCPPB4692_CP166090/XDZ21896 CAACGCGGACGCGGGATTAATGCGCTTGCA--CCACGGACATTCGATTCGCATCGATATG

CFBP7622_JAASRB010000002/NIJ9363 CAACCCGGATGCGGGATTAACGCGATTGCA--CCCCGGACATTCGATTCGCATCGCTATG

CPBF766_HG999364/CAG2083774 CAACCCGGATGCGGGATTAACGCGATTGCA--CCCCGGACATTCGATTCGCATCGCTATG

CPBF424_LR994544/CAE1133144 CAACCCGGATGCGGAATTAACGCCATTGCA--CCCCGGACATTCGATTCGCATCGCTATG

CPBF424_UIHB01000001/SUZ26707 CAACCCGGATGCGGAATTAACGCCATTGCA--CCCCGGACATTCGATTCGCATCGCTATG

* * * * ** **** ***** **** ****

CFBP8132_MIGM01000012/PPT37905 CCCCACTTCGCGATGCAGGTCGC-AGAAGTGGTCAGCGTGGCGCATTGTTGCCGCCCGTA

LMG19146_OEQG01000033/SOU04629 CCCCACTTCGCGATGCAGGTCGC-AGAAGTGGTCAGCGTGGCGCATTGTTGCCGCCCGTA

NCPPB1630_JPHE01000005/KER88775 CCCCACTTCGCGATGCAGGTCGC-AGAAGTGGTCAGCGTGGCGCATTGTTGCCGCCCGTA

CFBP8147_MIGP01000013/PPT47098 CCCCACTTCGCGATGCAGGTCGC-AGAAGTGGTCAGCGTGGCGCATTGTTGCCGCCCGTA

3272_JAATIV010000002/NJC03069 CCCCACTTCGCGATGCAGGTCGC-AGAAGTGGTCAGCGTGGCGCATTGTTGCCGCCCGTA

CITA14_LXIB01000070/OBR72431 CCCCACTTCGCGATGCAGGTCGC-AGAAGTGGTCAGCGTGGCGCATTGTTGCCGCCCGTA

NCPPB1832_JPHC01000067/KER79886 CCCCACTTCGCGATGCAGGTCGC-AGAAGTGGTCAGCGTGGCGCATTGTTGCCGCCCGTA

1314c_HG992337/CAE6703782 CCCCACTTCGCGATGCAGGTCGC-AGAAGTGGTCAGCGTGGCGCATTGTTGCCGCCCGTA

WHRI7004_JBEGFG010000005/MEQ7893 CCCCACTTCGCGATGCAGGTCGC-AGAAGTGGTCAGCGTGGCGCATTGTTGCCGCCCGTA

WHRI5708_JBEGEU010000001/MEQ7970 CCCCACTTCGCGATGCAGGTCGC-AGAAGTGGTCAGCGTGGCGCATTGTTGCCGCCCGTA

CFBP8149_JACHNN010000001/MBB6572 CCCCACTTCGCGATGCAGGTCGC-AGAAGTGGTCAGCGTGGCGCATTGTTGCCGCCCGTA

CFBP8149_MIGQ01000003/PPT88030 CCCCACTTCGCGATGCAGGTCGC-AGAAGTGGTCAGCGTGGCGCATTGTTGCCGCCCGTA

1311a_HG992336/CAE6704102 CCCCACTTCGCGATGCAGGTCGC-AGAAGTGGTCAGCGTGGCGCATTGTTGCCGCCCGTA

3140_JAASRH010000001/NIK31525 CCCCACTTCGCGATGCAGGTCGC-AGAAGTGGTCAGCGTGGCGCATTGTTGCCGCCCGTA

WHRI7064_JBEGFI010000004/MEQ7937 CCCCACTTCGCGATGCAGGTCGC-AGAAGTGGTCAGCGTGGCGCATTGTTGCCGCCCGTA

CFBP8140_JACIIT010000002/MBB6257 CCCCACTTCGCGATGCAGGTCGC-AGAAGTGGTCAGCGTGGCGCATTGTTGCCGCCCGTA

CFBP1022_MDRU01000008/PPU10530 CCCCACTTCGCGATGCAGGTCGC-AGAAGTGGTCAGCGTGGCGCATTGTTGCCGCCCGTA

CFBP7697_MDDZ01000001/PPU47721 CCCCACTTCGCGATGCAGGTCGC-AGAAGTGGTCAGCGTGGCGCATTGTTGCCGCCCGTA

CFBP8142_MIGO01000012/PPT66874 CCCCACTTCGCGATGCAGGTCGC-AGAATTGGTCAGCGTGGCGCATTGTTGCCGCCCGTA

CFBP6762_MDRW01000018/PPU23895 CCCCACTTCGCGATGCAGGTCGC-AGAAGTGGTCAGCGTGGCGCATTGTTGCCGCCCGTA

CFBP6762_OEQE01000006/SOT93765 CCCCACTTCGCGATGCAGGTCGC-AGAAGTGGTCAGCGTGGCGCATTGTTGCCGCCCGTA

CPBF1586_LR962897/CAD7376296 CCCCACTTCGCGATGCAGGTCGC-AGAAGTGGTCAGCGTGGCGCATTGTTGCCGCCCGTA

NBC5720_JAWIII010000009/MDV24505 CCAGACTTTGCGATGCAGGTCGCAAAAAGTGGTCAGCGTGCTGCATTGTTGCCGCCCATA

NBC5721_JBEOLS010000004/MER30529 CCAGACTTTGCGATGCAGGTCGCAAAAAGTGGTCAGCGTGCTGCATTGTTGCCGCCCATA

NCPPB2970_JAJFNJ020000003/MEC388 CTCGACTTCGCGATGCAGGTCGCAAAAATGGGTCAGCGTGCTGCATTGTTTCCGTACATA

NCPPB4692_CP166090/XDZ21896 CCCGACTTCGCGATGCAGGTCGC-AAAAATGGTCAGCGTGCTGCATTGTTGCCGCCCATA

CFBP7622_JAASRB010000002/NIJ9363 CCCGACTTCGCGATGCAGGTCGCAAAAAATGGTCAGCGTGCTGCATTGTTGCCGCCCATA

CPBF766_HG999364/CAG2083774 CCCGACTTCGCGATGCAGGTCGCAAAAAACGGTCAGCGTGCTGCATTGTTGCGGCCCATA

CPBF424_LR994544/CAE1133144 CCCGACTTCGCGATGCAGGTCGCAAAAAATGGCCAGCGTGCTGCATTGTTGCGGCCCATA

CPBF424_UIHB01000001/SUZ26707 CCCGACTTCGCGATGCAGGTCGCAAAAAATGGCCAGCGTGCTGCATTGTTGCGGCCCATA

* **** ************** * ** ** ******* ******** * * * **

CFBP8132_MIGM01000012/PPT37905 GCGGCCA--CCAAAAC-TCAAACGTGGATTCGCTATCGCGCGTTGTGCTTCACACCTATT

LMG19146_OEQG01000033/SOU04629 GCGGCCA--CCAAAAC-TCAAACGTGGATTCGCTATCGCGCGTTGTGCTTCACACCTATT

NCPPB1630_JPHE01000005/KER88775 GCGGCCA--CCAAAAC-TCAAACGTGGATTCGCTATCGCGCGTTGTGCTTCACACCTATT

CFBP8147_MIGP01000013/PPT47098 GCGGCCA--CCAAAAC-TCAAACGTGGATTCGCTATCGCGCGTTGTGCTTCACACCTATT

3272_JAATIV010000002/NJC03069 GCGGCCA--CCAAAAC-TCAAACGTGGATTCGCTATCGCGCGTTGTGCTTCACACCTATT

CITA14_LXIB01000070/OBR72431 GCGGCCA--CCAAAAC-TCAAACGTGGATTCGCTATCGCGCGTTGTGCTTCACACCCATT

NCPPB1832_JPHC01000067/KER79886 GCGGCCA--CCAAAAC-TCAAACGTGGATTCGCTATCGCGCGTTGTGCTTCACACCTATT

1314c_HG992337/CAE6703782 GCGGCCA--CCAAAAC-TCAAACGTGGATTCGCTATCGCGCGTTGTGCTTCACACCTATT

WHRI7004_JBEGFG010000005/MEQ7893 GCGGCCA--CCAAAAC-TCAAACGTGGATTCGCTATCGCGCGTTGTGCTTCACACCTATT

WHRI5708_JBEGEU010000001/MEQ7970 GCGGCCA--CCAAAAC-TCAAACGTGGATTCGCTATCGCGCGTTGTGCTTCACACCTATT

CFBP8149_JACHNN010000001/MBB6572 GCGGCCA--CCAAAAC-TCAAACGTGGATTCGCTATCGCGCGTTGTGCTTCACACCTATT

CFBP8149_MIGQ01000003/PPT88030 GCGGCCA--CCAAAAC-TCAAACGTGGATTCGCTATCGCGCGTTGTGCTTCACACCTATT

1311a_HG992336/CAE6704102 GCGGCCA--CCAAAAC-TCAAACGTGGATTCGCTATCGCGCGTTGTGCTTCACACCTATT

3140_JAASRH010000001/NIK31525 GCGGCCA--CCAAAAC-TCAAACGTGGATTCGCTATCGCGCGTTGTGCTTCACACCTATT

WHRI7064_JBEGFI010000004/MEQ7937 GCGGCCA--CCAAAAC-TCAAACGTGGATTCGCTATCGCGCGTTGTGCTTCACACCTATT

CFBP8140_JACIIT010000002/MBB6257 GCGGCCA--CCAAAAC-TCAAACGTGGATTCGCTATCGCGCGTTGTGCTTCACACCTATT

CFBP1022_MDRU01000008/PPU10530 GCGGCCA--CCAAAAC-TCAAACGTGGATTCGCTATCGCGCGTTGTGCTTCACACCTATT

CFBP7697_MDDZ01000001/PPU47721 GCGGCCA--CCAAAAC-TCAAACGTGGATTCGCTATCGCGCGTTGTGCTTCACACCTATT

CFBP8142_MIGO01000012/PPT66874 GCGGCCA--CCAAAAC-TCAAACGTGGATTCGCTATCGCGCGTTGTGCTTCACACCTATT

CFBP6762_MDRW01000018/PPU23895 GCGGCCA--CCAAAAC-TCAAACGTGGATTCGCTATCGCGCGTTGTGCTTCACACCTATT

CFBP6762_OEQE01000006/SOT93765 GCGGCCA--CCAAAAC-TCAAACGTGGATTCGCTATCGCGCGTTGTGCTTCACACCTATT

CPBF1586_LR962897/CAD7376296 GCGGCCA--CCAAAAC-TCAAACGTGGATTCGCTATCGCGCGTTGTGCTTCACACCTATT

NBC5720_JAWIII010000009/MDV24505 GCGGCCA-CCCGAGACATCACACGTGGATTCGCTATCGAGCGTTGCGCTTCAC-CTTATC

NBC5721_JBEOLS010000004/MER30529 GCGGCCA-CCCGAGACATCACACGTGGATTCGCTATCGAGCGTTGCGCTTCAC-CTTATC

NCPPB2970_JAJFNJ020000003/MEC388 GCGGCCG-CCCGAAACATCAGACGTGGATTCGCTATCGAGAGTTGCGCTTCAC-CCTATT

NCPPB4692_CP166090/XDZ21896 GCGGCCA-CCCGAAACATCAGACGTGGATTCGCTATCGCGCGTTGCGTTTCAC-CCTATT

CFBP7622_JAASRB010000002/NIJ9363 GCGGCCA-CCCCAAACATCAGACGTGGATTCGCTATCGCGCGTTGCGTTTCAC-CCTATT

CPBF766_HG999364/CAG2083774 GCGGCCA-CCCCAAACACAAGACGTGGATTCGCTATCGCGCGTTGCGTTTCAC-CCTATT

CPBF424_LR994544/CAE1133144 GCGGCCACCCCCAAGCACCAGACGTGGATTCGCTATCGCGCGTTGCGTTTCAC-CCTATT

CPBF424_UIHB01000001/SUZ26707 GCGGCCACCCCCAAGCACCAGACGTGGATTCGCTATCGCGCGTTGCGTTTCAC-CCTATT

****** ** * * * ***************** * **** * ***** * **

CFBP8132_MIGM01000012/PPT37905 TCATCAATACAAGCGAGTGGTATTCATGAAGGTTTCTTCCGCACCCCAGACAGCACGTTC

LMG19146_OEQG01000033/SOU04629 TCATCAATACAAGCGAGTGGTATTCATGAAGGTTTCTTCCGCACCCCAGACAGCACGTTC

NCPPB1630_JPHE01000005/KER88775 TCATCAATACAAGCGAGTGGTATTCATGAAGGTTTCTTCCGCACCCCAGACAGCACGTTC

CFBP8147_MIGP01000013/PPT47098 TCATCAATACAAGCGAGTGGTATTCATGAAGGTTTCTTCCGCACCCCAGACAGCACGTTC

3272_JAATIV010000002/NJC03069 TCATCAATACAAGCGAGTGGTATTCATGAAGGTTTCTTCCGCACCCCAGACAGCACGTTC

CITA14_LXIB01000070/OBR72431 TCATCAATACAAGCGAGTGGTATTCATGAAGGTTTCTTCCGCACCCCAGACAGCACGTTC

NCPPB1832_JPHC01000067/KER79886 TCATCAATACAAGCGAGTGGTATTCATGAAGGTTTCTTCCGCACCCCAGACAGCACGTTC

1314c_HG992337/CAE6703782 TCATCAATACAAGCGAGTGGTATTCATGAAGGTTTCTTCCGCACCCCAGACAGCACGTTC

WHRI7004_JBEGFG010000005/MEQ7893 TCATCAATACAAGCGAGTGGTATTCATGAAGGTTTCTTCCGCACCCCAGACAGCACGTTC

WHRI5708_JBEGEU010000001/MEQ7970 TCATCAATACAAGCGAGTGGTATTCATGAAGGTTTCTTCCGCACCCCAGACAGCACGTTC

CFBP8149_JACHNN010000001/MBB6572 TCATCAATACAAGCGAGTGGTATTCATGAAGGTTTCTTCCGCACCCCAGACAGCACGTTC

CFBP8149_MIGQ01000003/PPT88030 TCATCAATACAAGCGAGTGGTATTCATGAAGGTTTCTTCCGCACCCCAGACAGCACGTTC

1311a_HG992336/CAE6704102 TCATCAATACAAGCGAGTGGTATTCATGAAGGTTTCTTCCGCACCCCAGACAGCACGTTC

3140_JAASRH010000001/NIK31525 TCATCAATACAAGCGAGTGGTATTCATGAAGGTTTCTTCCGCACCCCAGACAGCACGTTC

WHRI7064_JBEGFI010000004/MEQ7937 TCATCAATACAAGCGAGTGGTATTCATGAAGGTTTCTTCCGCACCCCAGACAGCACGTTC

CFBP8140_JACIIT010000002/MBB6257 TCATCAATACAAGCGAGTGGTATTCATGAAGGTTTCTTCCGCACCCCAGACAGCACGTTC

CFBP1022_MDRU01000008/PPU10530 TCATCAATACAAGCGAGTGGTATTCATGAAGGTTTCTTCCGCACCCCAGACAGCACGTTC

CFBP7697_MDDZ01000001/PPU47721 TCATCAATACAAGCGAGTGGTATTCATGAAGGTTTCTTCCGCACCCCAGACAGCACGTTC

CFBP8142_MIGO01000012/PPT66874 TCATCAATACAAGCGAGTGGTATTCATGAAGGTTTCTTCCGCACCCCAGACAGCACGTTC

CFBP6762_MDRW01000018/PPU23895 TCATCAATACAAGCGAGTGGTATTCATGAAGGTTTCTTCCGCACCCCAGACAGCACGTTC

CFBP6762_OEQE01000006/SOT93765 TCATCAATACAAGCGAGTGGTATTCATGAAGGTTTCTTCCGCACCCCAGACAGCACGTTC

CPBF1586_LR962897/CAD7376296 TCATCAATACAAGCGAGTGGTATTCATGAAGGTTTCTTCCGCACCCCAGACAGCACGTTC

NBC5720_JAWIII010000009/MDV24505 TCATCAATACAAGCGAGTGGTATTCATGAAGGTTTCTTCCACACCCCAGGCAGCACACTC

NBC5721_JBEOLS010000004/MER30529 TCATCAATACAAGCGAGTGGTATTCATGAAGGTTTCTTCCACACCCCAGGCAGCACACTC

NCPPB2970_JAJFNJ020000003/MEC388 TCATCAATACAAGCGAGTGGTATTTATGAAGGTTTCTTCCGCACCCCAGACAGCACGTTC

NCPPB4692_CP166090/XDZ21896 TCATCAATACAAGCGAGTGGTATTCATGAAGGTTTCTTCCGCACCCCAGACAGCACAGTC

CFBP7622_JAASRB010000002/NIJ9363 TCATCAATACAAGCGAGTGGTATTCATGAAGGTTTCTTCCGCACCCCAGACAGCACATTC

CPBF766_HG999364/CAG2083774 TCATCAATACAAGCGAGTGGTATTCATGAAGGTTTCTTCCGCACCCCAGGCAGCACATTC

CPBF424_LR994544/CAE1133144 TCATCAATACAAGCGAGTGGTATTCATGAAGGTTTCTTCCGCACCCCAGGCAGCACATTC

CPBF424_UIHB01000001/SUZ26707 TCATCAATACAAGCGAGTGGTATTCATGAAGGTTTCTTCCGCACCCCAGGCAGCACATTC

************************ *************** ******** ****** **

CFBP8132_MIGM01000012/PPT37905 CTACGATAACCATGTGACCAATTCACCGAATGCCGAAAGGCAGAGTAGGCCGACCACAAG

LMG19146_OEQG01000033/SOU04629 CTACGATAACCATGTGACCAATTCACCGAATGCCGAAAGGCAGAGTAGGCCGACCACAAG

NCPPB1630_JPHE01000005/KER88775 CTACGATAACCATGTGACCAATTCACCGAATGCCGAAAGGCAGAGTAGGCCGACCACAAG

CFBP8147_MIGP01000013/PPT47098 CTACGATAACCATGTGACCAATTCACCGAATGCCGAAAGGCAGAGTAGGCCGACCACAAG

3272_JAATIV010000002/NJC03069 CTACGATAACCATGTGGCCAATTCACCGAATGCCGAAAGGCAGAGTAGGCCGACCACAAG

CITA14_LXIB01000070/OBR72431 CTACGATAACCATGTGGCCAATTCACCGAATGCCGAAAGGCAGAGTAGGCCGACCACAAG

NCPPB1832_JPHC01000067/KER79886 CTACGATAACCATGTGGCGAATTCACCGAATGCCGAAAGGCAGAGTAGGCCGACCACAAG

1314c_HG992337/CAE6703782 CTACGATAACCATGTGGCGAATTCACCGAATGCCGAAAGGCAGAGTAGGCCGACCACAAG

WHRI7004_JBEGFG010000005/MEQ7893 CTACGATAACCATGTGGCGAATTCACCGAATGCCGAAAGGCAGAGTAGGCCGACCACAAG

WHRI5708_JBEGEU010000001/MEQ7970 CTACGATAACCATGTGGCGAATTCACCGAATGCCGAAAGGCAGAGTAGGCCGACCACAAG

CFBP8149_JACHNN010000001/MBB6572 CTACGATAACCATGTGGCGAATTCACCGAATGCCGAAAGGCAGAGTAGGCCGACCACAAG

CFBP8149_MIGQ01000003/PPT88030 CTACGATAACCATGTGGCGAATTCACCGAATGCCGAAAGGCAGAGTAGGCCGACCACAAG

1311a_HG992336/CAE6704102 CTACGATAACCATGTGGCGAATTCACCGAATGCCGAAAGGCAGAGTAGGCCGACCACAAG

3140_JAASRH010000001/NIK31525 CTACGATAACCATGTGGCGAATTCACCGAATGCCGAAAGGCAGAGTAGGCCGACCACAAG

WHRI7064_JBEGFI010000004/MEQ7937 CTACGATAACCATGTGGCGAATTCACCGAATGCCGAAAGGCAGAGTAGGCCGACCACAAG

CFBP8140_JACIIT010000002/MBB6257 CTACGATAACCATGTGGCGAATTCACCGAATGCCGAAAGGCAGAGTAGGCCGACCACAAG

CFBP1022_MDRU01000008/PPU10530 CTACGATAACCATGTGGCCAATTCACCGAATGCCGAAAGGCAGAGTAGGCCGACCACAAG

CFBP7697_MDDZ01000001/PPU47721 CTACGATAACCATGTGGCCAATGCACCGAATGCCGAAAGGCAGAGTAGGCCGACCACAAG

CFBP8142_MIGO01000012/PPT66874 CTACGATAACCATGTGGCCAATTCACCGAATACCGAAAGGCAGAGTAGGCCGACCACAAG

CFBP6762_MDRW01000018/PPU23895 CTACGATAACCATGTGGCCAATTCACCGAATGCCGAAAGGCAGAGTAGGCCGACCACAAG

CFBP6762_OEQE01000006/SOT93765 CTACGATAACCATGTGGCCAATTCACCGAATGCCGAAAGGCAGAGTAGGCCGACCACAAG

CPBF1586_LR962897/CAD7376296 CTACGATAACCATGTGGCCAATTCACCGAATGCCGAAAGGCAGAGTAGGCCGACCACAAG

NBC5720_JAWIII010000009/MDV24505 CTACAATCACCATGTGGTGAATTCACCGAGCGCCGAAAAGCAAAGCAGGCCGCCCACAAA

NBC5721_JBEOLS010000004/MER30529 CTACAATCACCATGTGGTGAATTCACCGAGCGCCGAAAAGCAAAGCAGGCCGCCCACAAA

NCPPB2970_JAJFNJ020000003/MEC388 CTACAATAACCATGTAGTGAATTAACCGAGCGACGAGAGGCAAAGCAGGCCGACCACAAA

NCPPB4692_CP166090/XDZ21896 CTACAATAACCACGTAGTGAATTCACCGAGCGCCGAAAGGCAAAGCAGGCCGACCACAAA

CFBP7622_JAASRB010000002/NIJ9363 CTACACTAACCCCGTAGTGAATTCACCCAGCGCCGAAAGGCAAAGCAGGCCGACCACAAA

CPBF766_HG999364/CAG2083774 CTACACTAACCCCGTAGTGAATTCACCGAGCGCCGAAAGGCAAAGCAGGCCGACCACAAA

CPBF424_LR994544/CAE1133144 CTACACTAACCCCGTAGTGAATTCACCGAGCGCCGAAAGGCAAAGCAGGCCGACCACAAA

CPBF424_UIHB01000001/SUZ26707 CTACACTAACCCCGTAGTGAATTCACCGAGCGCCGAAAGGCAAAGCAGGCCGACCACAAA

**** * *** ** *** *** * *** * *** ** ****** ******

CFBP8132_MIGM01000012/PPT37905 AACCGACGGAGCGCTTGCACAACTTCCGGGACGTCCCGCCCCGAAAAGATATCGCACCGC

LMG19146_OEQG01000033/SOU04629 AACCGACGGAGCGCTTGCACAACTTCCGGGACGTCCCGCCCCGAAAAGATATCGCACCGC

NCPPB1630_JPHE01000005/KER88775 AACCGACGGAGCGCTTGCACAACTTCCGGGACGTCCCGCCCCGAAAAGATATCGCACCGC

CFBP8147_MIGP01000013/PPT47098 AACCGACGGAGCGCTTGCACAACTTCCGGGACGTCCCGCCCCGAAAAGATATCGCACCGC

3272_JAATIV010000002/NJC03069 AACCGACGGAGCGCTTGCACAACTTCCGGGACGTCCCGCCCCGAAAAGATATCGCACCGC

CITA14_LXIB01000070/OBR72431 AACCGACGGAGCGCTTGCACAACTTCCGGGACGTCCCGCCCCGAAAAGATATCGCACCGC

NCPPB1832_JPHC01000067/KER79886 AACCGACGGAGCGCTTGCACAACTTCCGGGACGTCCCGCCCCGAAAAGATATCGCACCGC

1314c_HG992337/CAE6703782 AACCGACGGAGCGCTTGCACAACTTCCGGGACGTCCCGCCCCGAAAAGATATCGCACCGC

WHRI7004_JBEGFG010000005/MEQ7893 AACCGACGGAGCGCTTGCACAACTTCCGGGACGTCCCGCCCCGAAAAGATATCGCACCGC

WHRI5708_JBEGEU010000001/MEQ7970 AACCGACGGAGCGCTTGCACAACTTCCGGGACGTCCCGCCCCGAAAAGATATCGCACCGC

CFBP8149_JACHNN010000001/MBB6572 AACCGACGGAGCGCTTGCACAACTTCCGGGACGTCCCGCCCCGAAAAGATATCGCACCGC

CFBP8149_MIGQ01000003/PPT88030 AACCGACGGAGCGCTTGCACAACTTCCGGGACGTCCCGCCCCGAAAAGATATCGCACCGC

1311a_HG992336/CAE6704102 AACCGACGGAGCGCTTGCACAACTTCCGGGACGTCCCGCCCCGAAAAGATATCGCACCGC

3140_JAASRH010000001/NIK31525 AACCGACGGAGCGCTTGCACAACTTCCGGGACGTCCCGCCCCGAAAAGATATCGCACCGC

WHRI7064_JBEGFI010000004/MEQ7937 AACCGACGGAGCGCTTGCACAACTTCCGGGACGTCCCGCCCCGAAAAGATATCGCACCGC

CFBP8140_JACIIT010000002/MBB6257 AACCGACGGAGCGCTTGCACAACTTCCGGGACGTCCCGCCCCGAAAAGATATCGCACCGC

CFBP1022_MDRU01000008/PPU10530 AACCGACGGAGCGCTTGCACAACTTCCGGGACGTCCCGCCCCGAAAAGATATCGCACCGC

CFBP7697_MDDZ01000001/PPU47721 AACCGACGGAGCGCTTGCACAACTTCCGGGACGTCCCGCCCCGAAAAGATATCGCACCGC

CFBP8142_MIGO01000012/PPT66874 AACCGACGGAGCGCTTGCACAACTTCCGGGACGTCCCGCCCCGAAAAGATATCGCACCGC

CFBP6762_MDRW01000018/PPU23895 AACCGACGGAGCGCTTGCACAACTTCCGGGACGTCCCGCCCCGAAAAGATATCGCACCGC

CFBP6762_OEQE01000006/SOT93765 AACCGACGGAGCGCTTGCACAACTTCCGGGACGTCCCGCCCCGAAAAGATATCGCACCGC

CPBF1586_LR962897/CAD7376296 AACCGACGGAGCGCTTGCACAACTTCCGGGACGTCCCGCCCCGAAAAGATATCGCACCGC

NBC5720_JAWIII010000009/MDV24505 AACCGATGGGCCGCTTGCACAGCTCCAGGGACGTTCCGCTCCGAAAAGATATCGCACCGC

NBC5721_JBEOLS010000004/MER30529 AACCGATGGGCCGCTTGCACAGCTCCAGGGACGTTCCGCTCCGAAAAGATATCGCACCGC

NCPPB2970_JAJFNJ020000003/MEC388 AACCGATGGGCCGCTTGCACAACTCCAGGGACGTCCCGCTCCGAAAAGATATCGCACCGC

NCPPB4692_CP166090/XDZ21896 AAGCGATGGGCCGCTTGCACAACTCCAGGGACGTTCCGCTCCGAAAAGATATCGCACCGC

CFBP7622_JAASRB010000002/NIJ9363 AAGCGATGGGCCGCTTGCACAGCTCCAGGGACGTTCCGCTCCGAAAAGATATCGCACCGC

CPBF766_HG999364/CAG2083774 AAGCGATGGGCCGCTTGCACAGCTCCAGGGACGTTCCGCTCCGAAAAGATATCGCACCGC

CPBF424_LR994544/CAE1133144 AAGCGATGGGCCGCTTGCACAGCTCCAGGGACGTTCCGCTCCGAAAAGATATCGCACCGC

CPBF424_UIHB01000001/SUZ26707 AAGCGATGGGCCGCTTGCACAGCTCCAGGGACGTTCCGCTCCGAAAAGATATCGCACCGC

** *** ** ********** ** * ******* **** ********************

CFBP8132_MIGM01000012/PPT37905 TTCCAAGCCCGCTCTGTCGACCGCGCAGGCACAGCAGATGCGACCACTTGGCATGCCGCC

LMG19146_OEQG01000033/SOU04629 TTCCAAGCCCGCTCTGTCGACCGCGCAGGCACAGCAGATGCGACCACTTGGCATGCCGCC

NCPPB1630_JPHE01000005/KER88775 TTCCAAGCCCGCTCTGTCGACCGCGCAGGCACAGCAGATGCGACCACTTGGCATGCCGCC

CFBP8147_MIGP01000013/PPT47098 TTCCAAGCCCGCTCTGTCGACCGCGCAGGCACAGCAGATGCGACCACTTGGCATGCCGCC

3272_JAATIV010000002/NJC03069 TTCCAAGCCCGCTCTGTCGACCGCGCAGGCACAGCAGATGCGGCCACTTGGCATGCCGCC

CITA14_LXIB01000070/OBR72431 TTCCAAGCCCGCTCTGTCGACCGCGCAGGCACAGCAGATGCGGCCACTTGGCATGCCGCC

NCPPB1832_JPHC01000067/KER79886 TTCCAAGCCCGCTCTGTCGACCGCGCAGGCACAGCAGATGCGGCCACTTGGTATGCCGCC

1314c_HG992337/CAE6703782 TTCCAAGCCCGCTCTGTCGACCGCGCAGGCACAGCAGATGCGGCCACTTGGCATGCCGCC

WHRI7004_JBEGFG010000005/MEQ7893 TTCCAAGCCCGCTCTGTCGACCGCGCAGGCACAGCAGATGCGGCCACTTGGCATGCCGCC

WHRI5708_JBEGEU010000001/MEQ7970 TTCCAAGCCCGCTCTGTCGACCGCGCAGGCACAGCAGATGCGGCCACTTGGCATGCCGCC

CFBP8149_JACHNN010000001/MBB6572 TTCCAAGCCCGCTCTGTCGACCGCGCAGGCACAGCAGATGCGGCCACTTGGCATGCCGCC

CFBP8149_MIGQ01000003/PPT88030 TTCCAAGCCCGCTCTGTCGACCGCGCAGGCACAGCAGATGCGGCCACTTGGCATGCCGCC

1311a_HG992336/CAE6704102 TTCCAAGCCCGCTCTATCGACCGCGCAGGCACAGCAGATGCGGCCACTTGGCATGCCGCC

3140_JAASRH010000001/NIK31525 TTCCAAGCCCGCTCTATCGACCGCGCAGGCACAGCAGATGCGGCCACTTGGCATGCCGCC

WHRI7064_JBEGFI010000004/MEQ7937 TTCCAAGCCCGCTCTGTCGACCGCGCAGGCACAGCAGATGCGGCCACTTGGCATGCCGCC

CFBP8140_JACIIT010000002/MBB6257 TTCCAAGCCCGCTCTGTCGACCGCGCAGGCACAGCAGATGCGGCCACTTGGCATGCCGCC

CFBP1022_MDRU01000008/PPU10530 TTCCAAGCCCGCTCTGTCGACCGCGCAGGCACAGCAGATGCGGCCACTTGGCATGCCGCC

CFBP7697_MDDZ01000001/PPU47721 TTCCAAGCCCGCTCTGTCGACCGCGCAGGCACAGCAGATGCGGCCACTTGGCATGCCGCC

CFBP8142_MIGO01000012/PPT66874 TTCCAAGCCCGCTCTGTCGACCGCGCAGGCACAGCAGATGCGGCCACTTGGCATGCCGCC

CFBP6762_MDRW01000018/PPU23895 TTCCAAGCCCGCTCTGTCGACCGCGCAGGCACAGCAGATGCGGCCACTTGGCATGCCGCC

CFBP6762_OEQE01000006/SOT93765 TTCCAAGCCCGCTCTGTCGACCGCGCAGGCACAGCAGATGCGGCCACTTGGCATGCCGCC

CPBF1586_LR962897/CAD7376296 TTCCAAGCCCGCTCTGTCGACCGCGCAGGCACAGCAGATGCGGCCACTTGGCATGCCGCC

NBC5720_JAWIII010000009/MDV24505 CCCCAAGCCCGTTCAGTCGACCGCACAGGCTCAGCAGATGCGACCACTTGGCATGCCGCC

NBC5721_JBEOLS010000004/MER30529 CCCCAAGCCCGTTCAGTCGACCGCACAGGCTCAGCAGATGCGACCACTTGGCATGCCGCC

NCPPB2970_JAJFNJ020000003/MEC388 TCCCAAGCTCGCTCAGTCGACCGCACAGGCTCAGCAGACGCGACCACTTGGCATGCCGCC

NCPPB4692_CP166090/XDZ21896 CCCCAAGCCCGCTTAGTCGACCGCACAGACTCAGCAGATGCGCCCACTTGGCATGCCGCC

CFBP7622_JAASRB010000002/NIJ9363 CCCCAAGCCTGCTCAGTCGAACGCACAGGCTCAGCAGATGCGGCCACTTGGCATGCCGCC

CPBF766_HG999364/CAG2083774 CCCCAAGCCTGCTCAGTCGAATGCACAGGCTCAGCAGATGCGGCCACTTGGCATGCCGCC

CPBF424_LR994544/CAE1133144 CCCCAAGCCTGCTCAGTCGAACGCACAGGCTCAGCAGATGCGGCCACTTGGCATGCCGCC

CPBF424_UIHB01000001/SUZ26707 CCCCAAGCCTGCTCAGTCGAACGCACAGGCTCAGCAGATGCGGCCACTTGGCATGCCGCC

****** * * **** ** *** * ******* *** ******** ********

CFBP8132_MIGM01000012/PPT37905 ATTCCACGCAGGCACCAGCAATGATTGCGGTCTTCATACGATTGCGGCACTGACCGGGCT

LMG19146_OEQG01000033/SOU04629 ATTCCACGCAGGCACCAGCAATGATTGCGGTCTTCATACGATTGCGGCACTGACCGGGCT

NCPPB1630_JPHE01000005/KER88775 ATTCCACGCAGGCACCAGCAATGATTGCGGTCTTCATACGATTGCGGCACTGACCGGGCT

CFBP8147_MIGP01000013/PPT47098 ATTCTACGCAGGCACCAGCAATGATTGCGGTCTTCATACGATTGCGGCACTGACTGGGCT

3272_JAATIV010000002/NJC03069 ATTCCACGCAGGCACCAGTAATGATTGCGGTCTTCATACGATTGCGGCACTGACCGGGCT

CITA14_LXIB01000070/OBR72431 ATTCCACGCAGGCACCAGTAATGATTGCGGTCTTCATACGATTGCGGCACTGACCGGGCT

NCPPB1832_JPHC01000067/KER79886 ATTCCACGCAGGCACCAGCAATGATTGCGGTCTTCATACGATTGCGGCACTGACCGGGCT

1314c_HG992337/CAE6703782 ATTCCACGCAGGCACCAGCAATGATTGCGGTCTTCATACGATTGCGGCACTGACCGGGCT

WHRI7004_JBEGFG010000005/MEQ7893 ATTCCACGCAGGCACCAGCAATGATTGCGGTCTTCATACGATTGCGGCACTGACCGGGCT

WHRI5708_JBEGEU010000001/MEQ7970 ATTCCACGCAGGCACCAGCAATGATTGCGGTCTTCATACGATTGCGGCACTGACCGGGCT

CFBP8149_JACHNN010000001/MBB6572 ATTCCACGCAGGCACCAGCAATGATTGCGGTCTTCATACGATTGCGGCACTGACCGGGCT

CFBP8149_MIGQ01000003/PPT88030 ATTCCACGCAGGCACCAGCAATGATTGCGGTCTTCATACGATTGCGGCACTGACCGGGCT

1311a_HG992336/CAE6704102 ATTCCACGCAGGCACCAGCAATGATTGCGGTCTTCATACGATTGCGGCACTGACCGGGCT

3140_JAASRH010000001/NIK31525 ATTCCACGCAGGCACCAGCAATGATTGCGGTCTTCATACGATTGCGGCACTGACCGGGCT

WHRI7064_JBEGFI010000004/MEQ7937 ATTCCACGCAGGCACCAGCAATGATTGCGGTCTTCATACGATTGCGGCACTGACCGGGCT

CFBP8140_JACIIT010000002/MBB6257 ATTCCACGCAGGCACCAGCAATGATTGCGGTCTTCATACGATTGCGGCACTGACCGGGCT

CFBP1022_MDRU01000008/PPU10530 ATTTCACGCAGGCACCAGCAACGATTGCGGTCTTCATACGATTGCGGCACTGACCGGGCT

CFBP7697_MDDZ01000001/PPU47721 ATTTCACGCAGGCACCAGCAACGATTGCGGTCTTCATACGATTGCGGCACTGACCGGGCT

CFBP8142_MIGO01000012/PPT66874 ATTTCACGCAGGCACCAGCAACGATTGCGGTCTTCATACGATTGCGGCACTGACCGGGCT

CFBP6762_MDRW01000018/PPU23895 ATTTCACGCAGGCACCAGCAACGATTGCGGTCTTCATACAATTGCGGCACTGACCGGGCT

CFBP6762_OEQE01000006/SOT93765 ATTTCACGCAGGCACCAGCAACGATTGCGGTCTTCATACAATTGCGGCACTGACCGGGCT

CPBF1586_LR962897/CAD7376296 ATTTCACGCAGGCACCAGCAACGATTGCGGTCTTCATACAATTGCGGCACTGACCGGGCT

NBC5720_JAWIII010000009/MDV24505 ATTCCACGCAGGCACCAGCAACGATTGCGGTCTTCATACGATTGCGGCGCTGACCGGGCT

NBC5721_JBEOLS010000004/MER30529 ATTCCACGCAGGCACCAGCAACGATTGCGGTCTTCATACGATTGCGGCGCTGACCGGGCT

NCPPB2970_JAJFNJ020000003/MEC388 ATTCCACGCCGGCACCAGCAACGATTGCGGTCTCCATACGATTGCGGCGCTGACCGGGCT

NCPPB4692_CP166090/XDZ21896 ATTCCACGCAGGCACCAGCAACGATTGCGGTCTTCATACGATTGCGGCACTGACCGGGCT

CFBP7622_JAASRB010000002/NIJ9363 ATTCCATGCAGGCACCAGTAACGACTGCGGTCTTCATACGATTGCGGCACTGACCGGGCT

CPBF766_HG999364/CAG2083774 ATTCCATGCAGGCACCAGTAACGATTGCGGTCTTCATACGATTGCGGCACTGACCGGGCT

CPBF424_LR994544/CAE1133144 ATTCCATGCAGGCACCAGTAACGATTGCGGTCTTCATACGATTGCGGCACTGACCGGGCT

CPBF424_UIHB01000001/SUZ26707 ATTCCATGCAGGCACCAGTAACGATTGCGGTCTTCATACGATTGCGGCACTGACCGGGCT

*** * ** ******** ** ** ******** ***** ******** ***** *****

CFBP8132_MIGM01000012/PPT37905 CGCCGAGGCGGACGTGGTCAACGGACTAGGCCTGACGCCAGACAACATTCAGTACATCAG

LMG19146_OEQG01000033/SOU04629 CGCCGAGGCGGACGTGGTCAACGGACTAGGCCTGACGCCAGACAACATTCAGTACATCAG

NCPPB1630_JPHE01000005/KER88775 CGCCGAGGCGGACGTGGTCAACGGACTAGGCCTGACGCCAGACAACATTCAGTACATCAG

CFBP8147_MIGP01000013/PPT47098 CGCCGAGGCGGACGTGGTCAACGGACTAGGCCTGACGCCAGACAACATTCAGTACATCAG

3272_JAATIV010000002/NJC03069 CGCCGAGGCGGACGTGGTCAACGGGCTTGGCCTGACGCCAGACAACATTCAGTACATCAG

CITA14_LXIB01000070/OBR72431 CGCCGAGGCGGACGTGGTCAACGGGCTTGGCCTGACGCCAGACAACATTCAGTACATCAG

NCPPB1832_JPHC01000067/KER79886 CGCCGAGGCGGACGTGGTCAACGGACTGGGCCTGACGCCAGACAACATTCAGTACATCAG

1314c_HG992337/CAE6703782 CGCCGAGGCGGACGTGGTCAACGGACTGGGCCTGACGCCAGACAACATTCAGTACATCAG

WHRI7004_JBEGFG010000005/MEQ7893 CGCCGAGGCGGACGTGGTCAACGGACTGGGCCTGACGCCAGACAACATTCAGTACATCAG

WHRI5708_JBEGEU010000001/MEQ7970 CGCCGAGGCGGACGTGGTCAACGGACTGGGCCTGACGCCAGACAACATTCAGTACATCAG

CFBP8149_JACHNN010000001/MBB6572 CGCCGAGGCGGACGTGGTCAACGGACTGGGCCTGACGCCAGACAATATTCAGTACATCAG

CFBP8149_MIGQ01000003/PPT88030 CGCCGAGGCGGACGTGGTCAACGGACTGGGCCTGACGCCAGACAATATTCAGTACATCAG

1311a_HG992336/CAE6704102 CGCCGAGGCGGACGTGGTCAACGGACTGGGCCTGACGCCAGACAACATTCAGTACATCAG

3140_JAASRH010000001/NIK31525 CGCCGAGGCGGACGTGGTCAACGGACTGGGCCTGACGCCAGACAACATTCAGTACATCAG

WHRI7064_JBEGFI010000004/MEQ7937 CGCCGAGGCGGACGTGGTCAACGGACTGGGCCTGACGCCAGACAACATTCAGTACATCAG

CFBP8140_JACIIT010000002/MBB6257 CGCCGAGGCGGACGTGGTCAACGGACTGGGCCTGACGCCAGACAACATTCAGTACATCAG

CFBP1022_MDRU01000008/PPU10530 CGCCGAGGCGGACGTGGTCAACGGGCTGGGCCTGACGCCAGACAACATTCAGTACATCAG

CFBP7697_MDDZ01000001/PPU47721 CGCCGAGGCGGACGTGGTCAACGGGCTGGGCCTGACGCCAGACAACATTCAGTACATCAG

CFBP8142_MIGO01000012/PPT66874 CGCCGAGGCGGACGTGGTCAACGGGCTGGGCCTGACGCCAGACAACATTCAGTACATCAG

CFBP6762_MDRW01000018/PPU23895 CGCCGAGGCGGACGTGGTCAACGGGCTGGGCCTGACGCCAGACAACATTCAGTACATCAG

CFBP6762_OEQE01000006/SOT93765 CGCCGAGGCGGACGTGGTCAACGGGCTGGGCCTGACGCCAGACAACATTCAGTACATCAG

CPBF1586_LR962897/CAD7376296 CGCCGAGGCGGACGTGGTCAACGGGCTGGGCCTGACGCCAGACAACATTCAGTACATCAG

NBC5720_JAWIII010000009/MDV24505 TCCTGAGGCGCATGTGGTCAACGGGCTGGGCCTGACACCAGACAACATTCAATACATTAG

NBC5721_JBEOLS010000004/MER30529 TCCTGAGGCGCATGTGGTCAACGGGCTGGGCCTGACACCAGACAACATTCAATACATTAG

NCPPB2970_JAJFNJ020000003/MEC388 TCCTGAGGCGCATGTGGTCAACGGGCTGGGCCTGACACCAGACAATATCCAATACATCAG

NCPPB4692_CP166090/XDZ21896 TCCTGAGGCGCATGTGGTCAACGGGCTGGGCCTGACAGCAGACAATATTCAACACATTAG

CFBP7622_JAASRB010000002/NIJ9363 CGCCGAGGCGGATGTTGTCAACGGGCTTGGCCTGACACCAGACAACATTCAATACATCAG

CPBF766_HG999364/CAG2083774 CGCCGAGGCGGATGTTGTCAACGGGCTTGGCCTGACACCAGACAACATTCAATACATCAG

CPBF424_LR994544/CAE1133144 CGCCGAGGCGGATGTTGTCAACGGGCTTGGCCTGACACCAGACAACATTCAATACATCAG

CPBF424_UIHB01000001/SUZ26707 CGCCGAGGCGGATGTTGTCAACGGGCTTGGCCTGACACCAGACAACATTCAATACATCAG

* ****** * ** ******** ** ******** ******* ** ** **** **

CFBP8132_MIGM01000012/PPT37905 CCAGCATGGCATGACGCCCGACCAGTTCACCTGGGCGATCACTAAATTCGAGAACTCCAA

LMG19146_OEQG01000033/SOU04629 CCAGCATGGCATGACGCCCGACCAGTTCACCTGGGCGATCACTAAATTCGAGAACTCCAA

NCPPB1630_JPHE01000005/KER88775 CCAGCATGGCATGACGCCCGACCAGTTCACCTGGGCGATCACTAAATTCGAGAACTCCAA

CFBP8147_MIGP01000013/PPT47098 CCAGCATGGCATGACGCCCGACCAGTTCACCTGGGCGATCACTAAATTCGAGAACTCCAA

3272_JAATIV010000002/NJC03069 CCAGCATGGCATGACGCCCGACCAGTTCACCTGGGCGATCACCAAATTCGAGAACTCCAA

CITA14_LXIB01000070/OBR72431 CCAGCATGGCATGACGCCCGACCAGTTCACCTGGGCGATCACCAAATTCGAGAACTCCAA

NCPPB1832_JPHC01000067/KER79886 CCAGCATGGCATGACGCCCGACCAGTTCACCTGGGCGATCACTAAATTCGAGAACTCCAA

1314c_HG992337/CAE6703782 CCAGCATGGCATGACGCCCGACCAGTTCACCTGGGCGATCACCAAATTCGAGAACTCCAA

WHRI7004_JBEGFG010000005/MEQ7893 CCAGCATGGCATGACGCCCGACCAGTTCACCTGGGCGATCACCAAATTCGAGAACTCCAA

WHRI5708_JBEGEU010000001/MEQ7970 CCAGCATGGCATGACGCCCGACCAGTTCACCTGGGCGATCACCAAATTCGAGAACTCCAA

CFBP8149_JACHNN010000001/MBB6572 CCAGCATGGCATGACGCCCGACCAGTTCACCTGGGCGATCACCAAATTCGAGAACTCCAA

CFBP8149_MIGQ01000003/PPT88030 CCAGCATGGCATGACGCCCGACCAGTTCACCTGGGCGATCACCAAATTCGAGAACTCCAA

1311a_HG992336/CAE6704102 CCAGCATGGCATGACGCCCGACCAGTTCACCTGGGCGATCACCAAATTCGAGAACTCCAA

3140_JAASRH010000001/NIK31525 CCAGCATGGCATGACGCCCGACCAGTTCACCTGGGCGATCACCAAATTCGAGAACTCCAA

WHRI7064_JBEGFI010000004/MEQ7937 CCAGCATGGCATGACGCCCGACCAGTTCACCTGGGCGATCACCAAATTCGAGAACTCCAA

CFBP8140_JACIIT010000002/MBB6257 CCAGCATGGCATGACGCCCGACCAGTTCACCTGGGCGATCACCAAATTCGAGAACTCCAA

CFBP1022_MDRU01000008/PPU10530 CCAGCATGGCATGACGCCCGACCAGTTCACTTGGGCGATCACCAAATTCGAGAACTCCAA

CFBP7697_MDDZ01000001/PPU47721 CCAGCATGGCATGACGCCCGACCAGTTCACTTGGGCGATCACCAAATTCGAGAACTCCAA

CFBP8142_MIGO01000012/PPT66874 CCAGCATGGCATGACGCCCGACCAGTTCACCTGGGCGATCACCAAATTCGAGAACTCCAA

CFBP6762_MDRW01000018/PPU23895 CCAGCATGGCATGACGCCCGACCAGTTCACCTGGGCGATCACCAAATTCGAGAACTCCAA

CFBP6762_OEQE01000006/SOT93765 CCAGCATGGCATGACGCCCGACCAGTTCACCTGGGCGATCACCAAATTCGAGAACTCCAA

CPBF1586_LR962897/CAD7376296 CCAGCATGGCATGACGCCCGACCAGTTCACCTGGGCGATCACCAAATTCGAGAACTCCAA

NBC5720_JAWIII010000009/MDV24505 CCAGCATGGCATGACGCCAGACCAATTTAC-TGGGCACTCACTAAATTAGGGCCTGTTAA

NBC5721_JBEOLS010000004/MER30529 CCAGCATGGCATGACGCCAGACCAATTTAC-TGGGCACTCACTAAATTAGGGCCTGTTAA

NCPPB2970_JAJFNJ020000003/MEC388 CCAACATGGCATGACGCCCGACCAATTTACCTGGGCACTCAGTAAATTAGAAAATGGCGG

NCPPB4692_CP166090/XDZ21896 CCAGCATGGCATGACACCCGACCAATTTACCTGGGCGATCACCAAATTGGAAAATGCCAG

CFBP7622_JAASRB010000002/NIJ9363 CCAGCATGGCATGACGCCAGACCAGTTTACCTGGGCGATTAGTAAATTCGAAAATGGCAG

CPBF766_HG999364/CAG2083774 CCAGCATGGCATGACGCCAGACCAGTTTACCTGGGCGATTAGTAAATTCGAAAATGGCAG

CPBF424_LR994544/CAE1133144 CCAGCATGGCATGACGCCAGACCAGTTTACCTGGGCGATTAGTAAATTCGAAAATGGCAG

CPBF424_UIHB01000001/SUZ26707 CCAGCATGGCATGACGCCAGACCAGTTTACCTGGGCGATTAGTAAATTCGAAAATGGCAG

*** *********** ** ***** ** ** ***** * * ***** *

CFBP8132_MIGM01000012/PPT37905 CGTCAGACACCAGCGCGGATCGCCGCAAGACCTTGCCAATGCGCTGCATGAGCTTCCAAA

LMG19146_OEQG01000033/SOU04629 CGTCAGACACCAGCGCGGATCGCCGCAAGACCTTGCCAATGCGCTGCATGAGCTTCCAAA

NCPPB1630_JPHE01000005/KER88775 CGTCAGACACCAGCGCGGATCGCCGCAAGACCTTGCCAATGCGCTGCATGAGCTTCCAAA

CFBP8147_MIGP01000013/PPT47098 CGTCAGACACCAGCGCGGATCGCCGCAAGACCTTGCCAATGCGCTGCATGAGCTTCCAAA

3272_JAATIV010000002/NJC03069 TGTCAGACACCAGCGCGGATCGCCGCAAGACCTTGCCAATGCGCTGCATGAGCTTCCAAA

CITA14_LXIB01000070/OBR72431 TGTCAGACACCAGCGCGGATCGCCGCAAGACCTTGCCAATGCGCTGCATGAGCTTCCAAA

NCPPB1832_JPHC01000067/KER79886 CGTCAGACACCAGCGCGGATCGCCGCAAGACCTTGCCAATGCGCTGCATGAGCTTCCAAA

1314c_HG992337/CAE6703782 CGTCAGACACCAGCGCGGATCGCCGCAAGACCTTGCCAATGCGCTGCATGAGCTTCCAAA

WHRI7004_JBEGFG010000005/MEQ7893 CGTCAGACACCAGCGCGGATCGCCGCAAGACCTCGCCAATGCGCTGCATGAGCTTCCAAA

WHRI5708_JBEGEU010000001/MEQ7970 CGTCAGACACCAGCGCGGATCGCCGCAAGACCTTGCCAATGCGCTGCATGAGCTTCCAAA

CFBP8149_JACHNN010000001/MBB6572 CGTCAGACACCAGCGCGGATCGCCGCAAGACCTTGCCAATGCGCTGCATGAGCTTCCAAA

CFBP8149_MIGQ01000003/PPT88030 CGTCAGACACCAGCGCGGATCGCCGCAAGACCTTGCCAATGCGCTGCATGAGCTTCCAAA

1311a_HG992336/CAE6704102 CGTCAGACACCAGCGCGGATCGCCGCAAGACCTTGCCAATGCGCTGCATGAGCTTCCAAA

3140_JAASRH010000001/NIK31525 CGTCAGACACCAGCGCGGATCGCCGCAAGACCTTGCCAATGCGCTGCATGAGCTTCCAAA

WHRI7064_JBEGFI010000004/MEQ7937 CGTCAGACACCAGCGCGGATCGCCGCAAGACCTTGCCAATGCGCTGCATGAGCTTCCAAA

CFBP8140_JACIIT010000002/MBB6257 CGTCAGACACCAGCGCGGATCGCCGCAAGACCTTGCCAATGCGCTGCATGAGCTTCCAAA

CFBP1022_MDRU01000008/PPU10530 CGTCAGACACCAGCGCGGATCGCCGCAAGACCTTGCCAATGCGCTGCATGAGCTTCCAAA

CFBP7697_MDDZ01000001/PPU47721 CGTCAGACACCAGCGCGGATCGCCGCAAGACCTTGCCAATGCGCTGCATGAGCTTCCAAA

CFBP8142_MIGO01000012/PPT66874 CGTCAGACACCAGCGCGGATCGCCGCAAGACCTTGCCAATGCGCTGCATGAGCTTCCAAA

CFBP6762_MDRW01000018/PPU23895 TGTCAGACACCAGCGCGGATCGCCGCAAGACCTTGCCAATGCGCTGCATGAGCTTCCAAA

CFBP6762_OEQE01000006/SOT93765 TGTCAGACACCAGCGCGGATCGCCGCAAGACCTTGCCAATGCGCTGCATGAGCTTCCAAA

CPBF1586_LR962897/CAD7376296 TGTCAGACACCAGCGCGGATCGCCGCAAGACCTTGCCAATGCGCTGCATGAGCTTCCAAA

NBC5720_JAWIII010000009/MDV24505 C-----ACATCCG---------------AAGCCCATCAACG------------------A

NBC5721_JBEOLS010000004/MER30529 C-----ACATCCG---------------AAGCCCATCAACG------------------A

NCPPB2970_JAJFNJ020000003/MEC388 TGTCAAACACCAGCGCGGATCGCCGGAAGAACTTGCCAGTGCACTGCATCAGCTTCCAGA

NCPPB4692_CP166090/XDZ21896 TGTCAGACACCAGCGCGGATCGCCGGAAGACCTTGCCAGTTCACTGCATCATCTTCCAGA

CFBP7622_JAASRB010000002/NIJ9363 TGTCAAACACCAGCGCGGATCGCCGCAAGACCTTGCAAGTGCGCTTCATCAGCTTCCAAA

CPBF766_HG999364/CAG2083774 TGTCAAACACCAGCGCGGATCGCCGCAAGACCTTGCAAGTGCGCTTCATCAGCTTCCAAA

CPBF424_LR994544/CAE1133144 TGTCAAACACCAGCGCGGATCGCCGCAAGACCTTGCAAGTGCACTTCATCAGCTTCCAAA

CPBF424_UIHB01000001/SUZ26707 TGTCAAACACCAGCGCGGATCGCCGCAAGACCTTGCAAGTGCACTTCATCAGCTTCCAAA

*** * * * * * *

CFBP8132_MIGM01000012/PPT37905 CTACGAAAAGATTGCGATCGGCATGGAGCGTCATTCCGGCATCGGACACCTTGTCGCAGG

LMG19146_OEQG01000033/SOU04629 CTACGAAAAGATTGCGATCGGCATGGAGCGTCATTCCGGCATCGGACACCTTGTCGCAGG

NCPPB1630_JPHE01000005/KER88775 CTACGAAAAGATTGCGATCGGCATGGAGCGTCATTCCGGCATCGGACACCTTGTCGCAGG

CFBP8147_MIGP01000013/PPT47098 CTACGAAAAGATTGCGATCGGCATGGAGCGTCATTCCGGCATCGGACACCTTGTCGCAGG

3272_JAATIV010000002/NJC03069 CTACGAAAAGATTGCGATCGGCATGGAGCGTCATTCCGGCATCGGACACCTTGTTGCCGG

CITA14_LXIB01000070/OBR72431 CTACGAAAAGATTGCGATCGGCATGGAGCGTCATTCCGGCATCGGACACCTTGTTGCCGG

NCPPB1832_JPHC01000067/KER79886 CTACGAAAAGATTGCGATCGGCATGGAGCGTCATTCCGGCATCGGACACCTTGTCGCAGG

1314c_HG992337/CAE6703782 CTACGAAAAGATTGCGATCGGCATGGAGCGTCATTCCGGCATCGGACACCTTGTTGCAGG

WHRI7004_JBEGFG010000005/MEQ7893 CTACGAAAAGATTGCGATCGGCATGGAGCGTCATTCCGGCATCGGACACCTTGTTGCAGG

WHRI5708_JBEGEU010000001/MEQ7970 CTACGAAAAGATTGCGATCGGCATGGAGCGTCATTCCGGCATCGGACACCTTGTCGCAGG

CFBP8149_JACHNN010000001/MBB6572 CTACGAAAAGATTGCGATCGGCATGGAGCGTCATTCCGGCATCGGACACCTTGTCGCAGG

CFBP8149_MIGQ01000003/PPT88030 CTACGAAAAGATTGCGATCGGCATGGAGCGTCATTCCGGCATCGGACACCTTGTCGCAGG

1311a_HG992336/CAE6704102 CTACGAAAAGATTGCGATCGGCATGGAGCGTCATTCCGGCATCGGACACCTTGTCGCAGG

3140_JAASRH010000001/NIK31525 CTACGAAAAGATTGCGATCGGCATGGAGCGTCATTCCGGCATCGGACACCTTGTCGCAGG

WHRI7064_JBEGFI010000004/MEQ7937 CTACGAAAAGATTGCGATCGGCATGGAGCGTCATTCCGGCATCGGACACCTTGTCGCAGG

CFBP8140_JACIIT010000002/MBB6257 CTACGAAAAGATTGCGATCGGCATGGAGCGTCATTCCGGCATCGGACACCTTGTCGCAGG

CFBP1022_MDRU01000008/PPU10530 CTACGAAAAGATTGCGATCGGCATGGAGCGTCATTCCGGCATCGGACACCTTGTCGCAGG

CFBP7697_MDDZ01000001/PPU47721 CTACGAAAAGATTGCGATCGGCATGGAGCGTCATTCCGGCATCGGACACCTTGTCGCAGG

CFBP8142_MIGO01000012/PPT66874 CTACGAAAAGATTGCGATCGGCATGGAGCGTCATTCCGGCATCGGACACCTTGTTGCAGG

CFBP6762_MDRW01000018/PPU23895 CTACGAAAAGATTGCGATCGGCATGGAGCGTCATTCCGGCATCGGACACCTTGTTGCAGG

CFBP6762_OEQE01000006/SOT93765 CTACGAAAAGATTGCGATCGGCATGGAGCGTCATTCCGGCATCGGACACCTTGTTGCAGG

CPBF1586_LR962897/CAD7376296 CTACGAAAAGATTGCGATCGGCATGGAGCGTCATTCCGGCATCGGACACCTTGTCGCAGG

NBC5720_JAWIII010000009/MDV24505 CTAGGACGAAGCTGAGGAATCCAAGGAACAT------GACATC---CAGCTTGTCGAAGC

NBC5721_JBEOLS010000004/MER30529 CTAGGACGAAGCTGAGGAATCCAAGGAACAT------GACATC---CAGCTTGTCGAAGC

NCPPB2970_JAJFNJ020000003/MEC388 TTACGAAAAGATTGCGCTCGGCATGGAGCGTCATTCAGGCATCGGACACCTGGTTGCTGC

NCPPB4692_CP166090/XDZ21896 CTA--AAAAAATTGCGATCGGCATGGAGCGTCATTCAGGCATCGTGCACCTGGTTGCTGC

CFBP7622_JAASRB010000002/NIJ9363 CTACGAAAAGATTGCGATCGGCATGGAGCGTCATTCAGGCATCGGACACCTCGTTGCTGC

CPBF766_HG999364/CAG2083774 CTACGAAAAGATTGCGATCGGCATGGAGCGTCATTCAGGCATCGGACACCTCGTTGCTGC

CPBF424_LR994544/CAE1133144 CTACGAAAAGATTGCGATCGGCATGGAGCGTCATTCAGGCATCGGACACCTCGTTGCTGC

CPBF424_UIHB01000001/SUZ26707 CTACGAAAAGATTGCGATCGGCATGGAGCGTCATTCAGGCATCGGACACCTCGTTGCTGC

** * * ** * ** *** * * * **** ** ** ** * *

CFBP8132_MIGM01000012/PPT37905 AATGCGCCAAGGCGAAAAATTGGTGATCTGGGATCGTCAGGTCAACCATGTGACAGAAGT

LMG19146_OEQG01000033/SOU04629 AATGCGCCAAGGCGAAAAATTGGTGATCTGGGATCGTCAGGTCAACCATGTGACAGAAGT

NCPPB1630_JPHE01000005/KER88775 AATGCGCCAAGGCGAAAAATTGGTGATCTGGGATCGTCAGGTCAACCATGTGACAGAAGT

CFBP8147_MIGP01000013/PPT47098 AATGCGCCAAGGCGAAAAATTGGTGATCTGGGATCGTCAGGTCAACCATGTGACAGAAGT

3272_JAATIV010000002/NJC03069 AATGCGCCAAGGCGAAAAACTGGTGATCTGGGATCGTCAGGTCAACCATGTGACAGAAGT

CITA14_LXIB01000070/OBR72431 AATGCGCCAAGGCGAAAAACTGGTGATCTGGGATCGTCAGGTCAACCATGTGACAGAAGT

NCPPB1832_JPHC01000067/KER79886 AATGCGCCAGGGCGAAAAATTGGTGATCTGGGATCGTCAGGTCAACCATGTGACAGAAGT

1314c_HG992337/CAE6703782 AATGCGCCAAGGCGAAAAACTGGTGATCTGGGATCGTCAGGTCAACCATGTGACAGAAGT

WHRI7004_JBEGFG010000005/MEQ7893 AATGCGCCAAGGCGAAAAACTGGTGATCTGGGATCGTCAGGTCAACCATGTGACAGAAGT

WHRI5708_JBEGEU010000001/MEQ7970 AATGCGCCAGGGCGAAAAACTGGTGATCTGGGATCGTCAGGTCAACCATGTGACAGAAGT

CFBP8149_JACHNN010000001/MBB6572 AATGCGCCAGGGCGAAAAACTGGTGATCTGGGATCGTCAGGTCAACCATGTGACAGAAGT

CFBP8149_MIGQ01000003/PPT88030 AATGCGCCAGGGCGAAAAACTGGTGATCTGGGATCGTCAGGTCAACCATGTGACAGAAGT

1311a_HG992336/CAE6704102 AATGCGCCAGGGCGAAAAACTGGTGATCTGGGATCGTCAGGTCAACCATGTGACAGAAGT

3140_JAASRH010000001/NIK31525 AATGCGCCAGGGCGAAAAACTGGTGATCTGGGATCGTCAGGTCAACCATGTGACAGAAGT

WHRI7064_JBEGFI010000004/MEQ7937 AATGCGCCAGGGCGAAAAACTGGTGATCTGGGATCGTCAGGTCAACCATGTGACAGAAGT

CFBP8140_JACIIT010000002/MBB6257 AATGCGCCAGGGCGAAAAACTGGTGATCTGGGATCGTCAGGTCAACCATGTGACAGAAGT

CFBP1022_MDRU01000008/PPU10530 AATGCGCCAAGGCGAAAAATTGGTGATCTGGGATCGTCAGGTCAACCATGTGACAGAAGT

CFBP7697_MDDZ01000001/PPU47721 AATGCGCCAAGGCGAAAAATTGGTGATCTGGGATCGTCAGGTCAACCATGTGACAGAAGT

CFBP8142_MIGO01000012/PPT66874 AATGCGCCAAGGCGAAAAACTGGTGATCTGGGATCGTCAGGTCAACCATGTGACAGAAGT

CFBP6762_MDRW01000018/PPU23895 AATGCGCCAAGGCGAAAAATTGGTGATCTGGGATCGTCAGGTCAACCATGTGACAGAAGT

CFBP6762_OEQE01000006/SOT93765 AATGCGCCAAGGCGAAAAATTGGTGATCTGGGATCGTCAGGTCAACCATGTGACAGAAGT

CPBF1586_LR962897/CAD7376296 AATGCGCCAAGGCGAAAAATTGGTGATCTGGGATCGTCAGGTCAACCATGTGACAGAAGT

NBC5720_JAWIII010000009/MDV24505 -----------GCGAGAAA------ATCCGG-----------------------------

NBC5721_JBEOLS010000004/MER30529 -----------GCGAGAAA------ATCCGG-----------------------------

NCPPB2970_JAJFNJ020000003/MEC388 GATGCGCCAAGGTGAAAAGCTGGTCATCTGGGATCGCCAGGTCAGCCATGTAACAGAAGT

NCPPB4692_CP166090/XDZ21896 GATGCGCCAAGGTGAAAAACTGGTCATCTGGGATCGCCTGGTCAGCCATGTAACAGAAGT

CFBP7622_JAASRB010000002/NIJ9363 GATGCGCCAAGGTGAAAAACTGGTCATCTGGGATCGCCAGGTCAGCCATGTAACAGAAGT

CPBF766_HG999364/CAG2083774 GATGCGCCAAGGTGAAAAACTGGTCATCTGGGATCGCCAGGTCAGCCATGTAACAGAAGT

CPBF424_LR994544/CAE1133144 GATGCGCCAAGGTGAAAAACTGGTCATCTGGGATCGCCAGGTCAGCCATGTAACAGAAGT

CPBF424_UIHB01000001/SUZ26707 GATGCGCCAAGGTGAAAAACTGGTCATCTGGGATCGCCAGGTCAGCCATGTAACAGAAGT

* ** ** *** **

CFBP8132_MIGM01000012/PPT37905 GAAAACCAGAGAGGAGCTATTGGATTATTTCAATAGCCATAATGTATCTAGTGTTCAAAC

LMG19146_OEQG01000033/SOU04629 GAAAACCAGAGAGGAGCTATTGGATTATTTCAATAGCCATAATGTATCTAGTGTTCAAAC

NCPPB1630_JPHE01000005/KER88775 GAAAACCAGAGAGGAGCTATTGGATTATTTCAATAGCCATAATGTATCTAGTGTTCAAAC

CFBP8147_MIGP01000013/PPT47098 GAAAACCAGAGAGGAGCTATTGGATTATTTCAATAGCCATAATGTATCTAGTGTTCAAAC

3272_JAATIV010000002/NJC03069 GAAAACCAGAGAGGAGCTATTGGATTATTTCAATAGCCATAATGTATCTAGTGTTCAAAC

CITA14_LXIB01000070/OBR72431 GAAAACCAGAGAGGAGCTATTGGATTATTTCAATAGCCATAATGTATCTAGTGTTCAAAC

NCPPB1832_JPHC01000067/KER79886 GAAAACCAGAGAGGAGCTATTGAATTATTTCAATAGCCATAATGTATCTAGTGTTCAAAC

1314c_HG992337/CAE6703782 GAAAACCAGAGAGGAGCTATTGAATTATTTCAATAGCCATAATGTATCTAGTGTTCAAAC

WHRI7004_JBEGFG010000005/MEQ7893 GAAAACCAGAGAGGAGCTATTGAATTATTTCAATAGCCATAATGTATCTAGTGTTCAAAC

WHRI5708_JBEGEU010000001/MEQ7970 GAAAACCAGAGAGGAGCTATTGAATTATTTCAATAGCCATAATGTATCTAGTGTTCAAAC

CFBP8149_JACHNN010000001/MBB6572 GAAAACCAGAGAGGAGCTATTGAATTATTTCAATAGCCATAATGTATCTAGTGTTCAAAC

CFBP8149_MIGQ01000003/PPT88030 GAAAACCAGAGAGGAGCTATTGAATTATTTCAATAGCCATAATGTATCTAGTGTTCAAAC

1311a_HG992336/CAE6704102 GAAAACCAGAGAGGAGCTATTGAATTATTTCAATAGCCATAATGTATCTAGTGTTCAAAC

3140_JAASRH010000001/NIK31525 GAAAACCAGAGAGGAGCTATTGAATTATTTCAATAGCCATAATGTATCTAGTGTTCAAAC

WHRI7064_JBEGFI010000004/MEQ7937 GAAAACCAGAGAGGAGCTATTGAATTATTTCAATATCCATAATGTATCTAGTGTTCAAAC

CFBP8140_JACIIT010000002/MBB6257 GAAAACCAGAGAGGAGCTATTGAATTATTTCAATAGCCATAATGTATCTAGTGTTCAAAC

CFBP1022_MDRU01000008/PPU10530 GAAAACCAGAGAGGAGCTATTGGATTATTTCAATAGCCATAATGTATCTAGTGTTCAAAC

CFBP7697_MDDZ01000001/PPU47721 GAAAACCAGAGAGGAGCTATTGGATTATTTCAATAGCCATAATGTATCTAGTGTTCAAAC

CFBP8142_MIGO01000012/PPT66874 GAAAACCAGAGAGGAGCTATTGAATTATTTCAATAGCCATAATGTATCTAGTGTTCAAAC

CFBP6762_MDRW01000018/PPU23895 GAAAACCAGAGAGGAGCTATTGGATTATTTCAATAGCCATAATGTATCTAGTGTTCAAAC

CFBP6762_OEQE01000006/SOT93765 GAAAACCAGAGAGGAGCTATTGGATTATTTCAATAGCCATAATGTATCTAGTGTTCAAAC

CPBF1586_LR962897/CAD7376296 GAAAACCAGAGAGGAGCTATTGGATTATTTCAATAGCCATAATGTATCTAGTGTTCAAAC

NBC5720_JAWIII010000009/MDV24505 ------------------------------------------------------------

NBC5721_JBEOLS010000004/MER30529 ------------------------------------------------------------

NCPPB2970_JAJFNJ020000003/MEC388 GCGAACGAGAGAGGAGTTATTGAATTATTTTAATAGCAATAATGTGTCAAATGTTCAAAC

NCPPB4692_CP166090/XDZ21896 GCGAACGAGAGACGAGTTATTGAATTATTTCAATAGCAATAATGTGTCGAGTGTTCAAAC

CFBP7622_JAASRB010000002/NIJ9363 GCGAACAAGAGAGGAGTTATTGCATTATTTCAATAGCAATAATGTATCAAGTGTTCAAAC

CPBF766_HG999364/CAG2083774 GCGAACAAGAGAGGAGTTATTGCATTATTTCAATAGCAATAATGTATCAAGTGTTCAAAC

CPBF424_LR994544/CAE1133144 GCGAACGAGAGAGGAGTTATTGCATTATTTCAATAGCAATAATGTATCAAGTGTTCAAAC

CPBF424_UIHB01000001/SUZ26707 GCGAACGAGAGAGGAGTTATTGCATTATTTCAATAGCAATAATGTATCAAGTGTTCAAAC

CFBP8132_MIGM01000012/PPT37905 GTGGTCCCGTCAATGATCTCCAGTAGTCCGTAGCTATCCGCAGGCGATTGGAAAGAACCA

LMG19146_OEQG01000033/SOU04629 GTGGTCCCGTCAATGATCTCCAGTAGTCCGTAGCTATCCGCAGGCGATTGGAAAGAACCA

NCPPB1630_JPHE01000005/KER88775 GTGGTCCCGTCAATGATCTCCAGTAGTCCGTAGCTATCCGCAGGCGATTGGAAAGAACCA

CFBP8147_MIGP01000013/PPT47098 GTGGTCCCGTCAATGATCTCCAGTAGTCCGTAGCTATCCGCAGGCGATTGGAAAGAACCA

3272_JAATIV010000002/NJC03069 GTGGTCCCGTCAATGATCTCCAGTAGTCCGTAGCTATCCGCAGGCGACTGGAAAGAACCA

CITA14_LXIB01000070/OBR72431 GTGGTCCCGTCAATGATCTCCAGTAGTCCGTAGCTATCCGCAGGCGACTGGAAAGAACCA

NCPPB1832_JPHC01000067/KER79886 GTGGTCCCGTCAATGATCTCCAGTAGTCCGTAGCTATCCGCAGGCGACTGGAAAGAACCA

1314c_HG992337/CAE6703782 GTGGTCCCGTCAATGATCTCCAGTAGTCCGTAGCTATCCGCAGGCGACTGGAAATAACCA

WHRI7004_JBEGFG010000005/MEQ7893 GTGGTCCCGTCAATGATCTCCAGTAGTCCGTAGCTATCCGCAGGCGACTGGAAATAACCA

WHRI5708_JBEGEU010000001/MEQ7970 GTGGTCCCGTCAATGATCTCCAGTAGTCCGTAGCTATCCGCAGGCGACTGGAAAGAACCA

CFBP8149_JACHNN010000001/MBB6572 GTGGTCCCGTCAATGATCTCCAGTAGTCCGTAGCTATCCGCAGGCGACTGGAAAGAACCA

CFBP8149_MIGQ01000003/PPT88030 GTGGTCCCGTCAATGATCTCCAGTAGTCCGTAGCTATCCGCAGGCGACTGGAAAGAACCA

1311a_HG992336/CAE6704102 GTGGTCCCGTCAATGATCTCCAGTAGTCCGTAGCTATCCGCAGGCGACTGGAAAGAACCA

3140_JAASRH010000001/NIK31525 GTGGTCCCGTCAATGATCTCCAGTAGTCCGTAGCTATCCGCAGGCGACTGGAAAGAACCA

WHRI7064_JBEGFI010000004/MEQ7937 GTGGTCCCGTCAATGATCTCCAGTAGTCCGTAGCTATCCGCAGGCGACTGGAAAGAACCA

CFBP8140_JACIIT010000002/MBB6257 GTGGTCCCGTCAATGATCTCCAGTAGTCCGTAGCTATCCGCAGGCGACTGGAAAGAACCA

CFBP1022_MDRU01000008/PPU10530 GTGGTCCCGTCAATGATCTCCAGTAGTCCGTAGCTATCCGCAGGCGACTGGAAATAACCA

CFBP7697_MDDZ01000001/PPU47721 GTGGTCCCGTCAATGATCTCCAGTAGTCCGTAGCTATCCGCAGGCGACTGGAAATAACCA

CFBP8142_MIGO01000012/PPT66874 GTGGTCCCGTCAATGATCTCCAGTAGTCCGTAGCTATCCGCAGGCGACTGGAAAGAACCA

CFBP6762_MDRW01000018/PPU23895 GTGGTCCCGTCAATGATCTCCAGTAGTCCGTAGCTATCCGCAGGCGACTGGAAAGAACCA

CFBP6762_OEQE01000006/SOT93765 GTGGTCCCGTCAATGATCTCCAGTAGTCCGTAGCTATCCGCAGGCGACTGGAAAGAACCA

CPBF1586_LR962897/CAD7376296 GTGGTCCCGTCAATGATCTCCAGTAGTCCGTAGCTATCCGCAGGCGACTGGAAAGAACCA

NBC5720_JAWIII010000009/MDV24505 --------------------CGGTAGCCTTT---------CAAGCGACGGAACAGT----

NBC5721_JBEOLS010000004/MER30529 --------------------CGGTAGCCTTT---------CAAGCGACGGAACAGT----

NCPPB2970_JAJFNJ020000003/MEC388 GTGGTCCCGTCAGTGATCCCCAGTAGTCCGTAGCTATTCGCAGGCAACTGGTGAGAATCC

NCPPB4692_CP166090/XDZ21896 GTGGTCCCGTCAGTGATCCCCGGTAGCCCGTAGCTATCCGCAGGCAACCGGAAA-AACCC

CFBP7622_JAASRB010000002/NIJ9363 GTGGTCCCGTCAGTGATCCCCGGTAGCCCGCAGCTATCCGCAGGCAACTGAAAAAAACC-

CPBF766_HG999364/CAG2083774 GTGGTCCCGTCAGTGATCCCCGGTAGCCCGCAGCTATCCGCAGGCAACTGAAAAAAACC-

CPBF424_LR994544/CAE1133144 GTGGTCCCGTCAGTGATCCCCGGTAGCCCGCAGCTATCCGCAGGAAACTGAAAAAAACC-

CPBF424_UIHB01000001/SUZ26707 GTGGTCCCGTCAGTGATCCCCGGTAGCCCGCAGCTATCCGCAGGAAACTGAAAAAAACC-

* **** * ** * * * *

CFBP8132_MIGM01000012/PPT37905 TCTGT---

LMG19146_OEQG01000033/SOU04629 TCTGT---

NCPPB1630_JPHE01000005/KER88775 TCTGT---

CFBP8147_MIGP01000013/PPT47098 TCTGT---

3272_JAATIV010000002/NJC03069 TCTGT---

CITA14_LXIB01000070/OBR72431 TCTGT---

NCPPB1832_JPHC01000067/KER79886 TCTGT---

1314c_HG992337/CAE6703782 TCTGT---

WHRI7004_JBEGFG010000005/MEQ7893 TCTGT---

WHRI5708_JBEGEU010000001/MEQ7970 TCTGT---

CFBP8149_JACHNN010000001/MBB6572 TCTGT---

CFBP8149_MIGQ01000003/PPT88030 TCTGT---

1311a_HG992336/CAE6704102 TCTGT---

3140_JAASRH010000001/NIK31525 TCTGT---

WHRI7064_JBEGFI010000004/MEQ7937 TCTGT---

CFBP8140_JACIIT010000002/MBB6257 TCTGT---

CFBP1022_MDRU01000008/PPU10530 TCTGT---

CFBP7697_MDDZ01000001/PPU47721 TCTGT---

CFBP8142_MIGO01000012/PPT66874 TCTGT---

CFBP6762_MDRW01000018/PPU23895 TCTGT---

CFBP6762_OEQE01000006/SOT93765 TCTGT---

CPBF1586_LR962897/CAD7376296 TCTGT---

NBC5720_JAWIII010000009/MDV24505 --------

NBC5721_JBEOLS010000004/MER30529 --------

NCPPB2970_JAJFNJ020000003/MEC388 TCTAC---

NCPPB4692_CP166090/XDZ21896 TCTACAAC

CFBP7622_JAASRB010000002/NIJ9363 TCTGCA--

CPBF766_HG999364/CAG2083774 TCTGCA--

CPBF424_LR994544/CAE1133144 TCTGCA--

CPBF424_UIHB01000001/SUZ26707 TCTGCA--

References

Bailey,T.L. *et al.* (2006) MEME: discovering and analyzing DNA and protein sequence motifs. *Nucleic Acids Res.*, **34**, W369–W373.

Bongrand,C. *et al.* (2012) Characterization of the promoter, MxiE box and 5′ UTR of genes controlled by the activity of the type III secretion apparatus in Shigella flexneri. *PLoS One*, **7**, e32862.

Brandes,N. *et al.* (2022) ProteinBERT: a universal deep-learning model of protein sequence and function. *Bioinformatics*, **38**, 2102–2110.

Brutinel,E.D. *et al.* (2009) Functional domains of ExsA, the transcriptional activator of the *Pseudomonas aeruginosa* type III secretion system. *J. Bacteriol.*, **191**, 3811–3821.

Burstein,D. *et al.* (2015) Novel type III effectors in *Pseudomonas aeruginosa*. *MBio*, **6**, e00161-15.

Elnaggar,A. *et al.* (2022) ProtTrans: Toward understanding the language of life through self-supervised learning. *IEEE Trans. Pattern Anal. Mach. Intell.*, **44**, 7112–7127.

Gazi,A.D. *et al.* (2012) Phylogenetic analysis of a gene cluster encoding an additional, rhizobial-like type III secretion system that is narrowly distributed among *Pseudomonas syringae* strains. *BMC Microbiol.*, **12**, 1–15.

Hobbs,C.K. *et al.* (2016) Computational approach to predict species-specific type III secretion system (T3SS) effectors using single and multiple genomes. *BMC Genomics*, **17**, 1048.

Kawashima,S. *et al.* (2008) AAindex: Amino acid index database, progress report 2008. *Nucleic Acids Res.*, **36**, D202–D205.

Koebnik,R. *et al.* (2006) Specific binding of the *Xanthomonas campestris* pv. vesicatoria AraC-type transcriptional activator HrpX to plant-inducible promoter boxes. *J. Bacteriol.*, **188**, 7652–7660.

Krause,A. *et al.* (2002) Mutational and transcriptional analysis of the type III secretion system of *Bradyrhizobium japonicum*. *Mol. Plant. Microbe. Interact.*, **15**, 1228–1235.

Lin,Z. *et al.* (2023) Evolutionary-scale prediction of atomic-level protein structure with a language model. *Science (80-. ).*, **379**, 1123–1130.

Mavris,M. *et al.* (2002) Identification of the cis-acting site involved in activation of promoters regulated by activity of the type III secretion apparatus in *Shigella flexneri*. *J. Bacteriol.*, **184**, 6751–6759.

McCann,H.C. and Guttman,D.S. (2008) Evolution of the type III secretion system and its effectors in plant-microbe interactions. *New Phytol.*, **177**, 33–47.

Mukaihara,T. *et al.* (2004) Genetic screening of Hrp type III-related pathogenicity genes controlled by the HrpB transcriptional activator in *Ralstonia solanacearum*. *Mol. Microbiol.*, **54**, 863–875.

Orfei,B. *et al.* (2023) Race-specific genotypes of *Pseudomonas syringae* pv. tomato are defined by the presence of mobile DNA elements within the genome. *Front. Plant Sci.*, **14**, 1197706.

Steinegger,M. and Söding,J. (2017) MMseqs2 enables sensitive protein sequence searching for the analysis of massive data sets. *Nat. Biotechnol. 2017 3511*, **35**, 1026–1028.

Wagner,N. *et al.* (2023) Complete genome sequence of an Israeli isolate of *Xanthomonas hortorum* pv. pelargonii strain 305 and novel type III effectors identified in *Xanthomonas*. *Front. Plant Sci.*, **14**, 1817.

Wagner,N. *et al.* (2022) Natural language processing approach to model the secretion signal of type III effectors. *Front. Plant Sci.*, **13**, 4339.

Zwiesler-Vollick,J. *et al.* (2002) Identification of novel hrp-regulated genes through functional genomic analysis of the *Pseudomonas syringae* pv. tomato DC3000 genome. *Mol. Microbiol.*, **45**, 1207–1218.
